# Body Mechanics, Optimality, and Sensory Feedback in the Human Control of Complex Objects

**DOI:** 10.1162/neco_a_01576

**Published:** 2023-04-18

**Authors:** Reza Sharif Razavian, Mohsen Sadeghi, Salah Bazzi, Rashida Nayeem, Dagmar Sternad

**Affiliations:** Department of Mechanical Engineering, Northern Arizona University, Flagstaff, AZ 86011, U.S.A.; Department of Biology and Department of Electrical and Computer Engineering, Northeastern University, Boston, MA 02115, U.S.A.; Department of Electrical and Computer Engineering and Institute for Experiential Robotics, Northeastern University, Boston, MA 02115, U.S.A.; Department of Electrical and Computer Engineering, Northeastern University, Boston, MA 02115, U.S.A.; Departments of Biology, Electrical and Computer Engineering, and Physics, Institute for Experiential Robotics, Northeastern University, Boston, MA 02115, U.S.A.

## Abstract

Humans are adept at a wide variety of motor skills, including the handling of complex objects and using tools. Advances to understand the control of voluntary goal-directed movements have focused on simple behaviors such as reaching, uncoupled to any additional object dynamics. Under these simplified conditions, basic elements of motor control, such as the roles of body mechanics, objective functions, and sensory feedback, have been characterized. However, these elements have mostly been examined in isolation, and the interactions between these elements have received less attention. This study examined a task with internal dynamics, inspired by the daily skill of transporting a cup of coffee, with additional expected or unexpected perturbations to probe the structure of the controller. Using optimal feedback control (OFC) as the basis, it proved necessary to endow the model of the body with mechanical impedance to generate the kinematic features observed in the human experimental data. The addition of mechanical impedance revealed that simulated movements were no longer sensitively dependent on the objective function, a highly debated cornerstone of optimal control. Further, feedforward replay of the control inputs was similarly successful in coping with perturbations as when feedback, or sensory information, was included. These findings suggest that when the control model incorporates a representation of the mechanical properties of the limb, that is, embodies its dynamics, the specific objective function and sensory feedback become less critical, and complex interactions with dynamic objects can be successfully managed.

## 1. Introduction

Humans show remarkable dexterity in a wide range of skills, from juggling balls to a host of seemingly mundane actions in everyday life. For example, reaching for a glass of wine, swirling it, and leading it to one’s mouth to drink involves interacting with–and controlling–the complex fluid dynamics acting on the hand. Handling such complex and potentially even chaotic dynamics is a feat that robots have failed to accomplish so far. Also in human motor neuroscience, most research on human movement control to date has only addressed relatively simple movements, such as moving one’s hand from point to point, void of any dynamics arising from objects and interactions with the environment. Consequently, models of human motor control may have remained relatively simple as they did not have to deal with the complex and sometimes unpredictable interaction dynamics. Human manipulation of objects–the essence of tool use–has remained largely beyond neuroscientists’ reach. To better understand how the brain controls and coordinates movements, we must look beyond unconstrained reaching movements and study richer and more naturalistic behaviors to reveal the underlying control principles.

Characterization of a dynamical system and its control requires exciting inputs that perturb the dynamical modes (Åström & Torsten, 1965; Åström & Eykhoff, 1971). Hence, the application of external perturbations has been the widely used method to interrogate the human motor system (Shadmehr & Mussa-Ivaldi, 1994; Krakauer et al., 2000; Burdet et al., 2001). Beyond controlled external perturbations, another way to excite the motor system and reveal its dynamical modes is to expose the motor system to interactions with the world as they are naturally inherent in daily activities. Only few previous studies examined more naturalistic tasks, such as playing pool billiards (Haar et al., 2020; Haar & Faisal, 2020), bouncing a ball on a paddle (Sternad et al., 2001), and interacting with dynamically complex objects, such as carrying a cup of coffee (Nasseroleslami et al., 2014; Maurice et al., 2018) or striking a target with a whip (Krotov et al., 2022; Nah et al., 2020). The study of such naturalistic interactions suggested that most of the conventional control objectives, such as maximizing smoothness or minimizing effort, are not sufficient to account for human behavior (Maurice et al., 2018; Nayeem et al., 2021). When investigating a task such as transporting a cup of coffee, more nuanced and object-centric objectives seem to be at play that are concerned with the stability and predictability of the interactions (Bazzi et al., 2018; Bazzi & Sternad, 2020b), and with minimizing the transient behavior of the object that is being controlled (Nayeem et al., 2021). Expanding on these insights, this study aims to understand complex interactions, with a focus on how embodiment affects higher-level control objectives, as well as the role of sensory feedback in the control process.

In interactions with the external world, physical properties of the body, especially the mechanical impedance of the limbs, have been identified as critical (Hogan, 1984, 2022). Due to its ability to store and dissipate mechanical energy, impedance affords stability in the presence of sensorimotor delays (Rancourt & Hogan, 2001) or when facing perturbations (Burdet et al., 2001). Impedance has been proposed as a dynamic primitive that interfaces the body with the external world and shapes basic patterns of movements (Hogan & Sternad, 2012, 2013). The controller determines the zero-force trajectories in interactions with the environment, and the observable movements arise as a consequence of this interaction (Hermus et al., 2020). From a physiological perspective, the central nervous system sets virtual or equilibrium-point trajectories via tuning of the phasic and tonic stretch reflexes (Feldman, 1986; Latash, 1992; Gomi & Kawato, 1997). However, to create rich behavior, the computational principles that generate more complex reference trajectories have remained elusive. This work sought to uncover how the inherent mechanics of the body–the “embodied intelligence” – and the characteristics of the neural controller influence one another in both the choice of control objectives and the use of sensory feedback.

In computational motor neuroscience, several lines of research have pursued the framework of optimal control as a model for the human motor controller (Todorov, 2004; Scott, 2004). The fundamental assumption in the optimal control framework is that the brain selects movements to optimize a certain objective or cost function. The nature of this objective function has been the focus of many studies, and several candidates have been proposed that are either based on kinematics (Flash & Hogan, 1985; Dingwell et al., 2004; Leib & Karniel, 2012; Svinin et al., 2019; Ronsse et al., 2010; Harris & Wolpert, 1998), kinetics (Todorov & Jordan, 2002b; Uno et al., 1989), or energetics (Anderson & Pandy, 2001; Wong et al., 2021). All of these objectives described human movements well in their respective experimental contexts. However, these costs may not be completely independent and free to choose by the brain. Wong et al. (2021) showed that kinematic smoothness arose as a by-product of physiological energy minimization, without any explicit kinematics-related cost function. This finding suggests that including more bio-fidelic features in the control model may achieve desired features in human behavior in a less top-down-dictated fashion. In this vein, mechanical impedance of the body, with its ability to store and dissipate energy, plays a critical role in defining the energetic landscape of the movements. Therefore, it is expected that the energy buffering of impedance affects properties of the controller, specifically the objective function.

The neural controller possesses feedback and feedforward control pathways that were recognized as early as 1899 (Woodworth, 1899). However, the exact interplay between feedforward and feedback control mechanisms and their dependence on different task scenarios is still under debate (Crevecoeur & Scott, 2014; Yeo et al., 2016). While an essential element for many accuracy-requiring tasks, for rapidly evolving physical interactions, such as carrying a cup filled with sloshing coffee, the sensorimotor information transmission is too slow to ensure successful feedbackbased corrections. A more likely mechanism for instantaneous interaction dynamics is mechanical impedance as it affords rapid corrective responses without relying on neural signal transmission (Rancourt & Hogan, 2001; Abbs & Gracco, 1984). Indeed, previous results on interaction with the cup-and-ball system already highlighted that simple feedforward models that included impedance reproduced behavior well (Maurice et al., 2018; Nayeem et al., 2021). However, it remains unclear how feedback and feedforward processes interact with mechanical impedance in situations that require unplanned and rapid, yet complex, responses. This study applies expected and unexpected perturbations to examine the interplay of feedforward and feedback processes in interaction with impedance.

In overview, this study used the dynamically complex experimental task of transporting a cup of coffee to examine how interfacing the controller with the body’s mechanical impedance affects the resulting behavior. To generate rich and informative data, the experiment included predictable and unpredictable perturbations in addition to the complex interaction forces generated through the object’s internal dynamics. The optimal feedback control (OFC) framework was used as the model for the neural controller (Todorov & Jordan, 2002a). Simulations scrutinized how the inclusion of impedance in the model changed its behavior under different objective functions and involvement of sensory feedback. We hypothesized that mechanical impedance was a critical component to accurately generate interactions with the complex object (hypothesis 1), that the specifics of the objective function become less prominent due to the energy buffering of impedance (hypothesis 2), and that, for the same reasons, sensory feedback plays a subordinate role in this dynamically rich behavior (hypothesis 3).

## 2 Methods

### 2.1. Experiments.

#### 2.1.1 Participants.

Eleven healthy right-handed individuals participated in the experiment (19 to 25 years old, 8 females). None of the participants had any history of neurological disorders or biomechanical injuries in their upper limbs. Each subject provided written consent to the experimental procedures prior to participation. All subjects were given monetary compensation for their participation. The study was approved by the Institutional Review Board of Northeastern University.

#### 2.1.2 Moving a Complex Object: A Cup with a Ball Rolling Inside.

**Figure 1:**
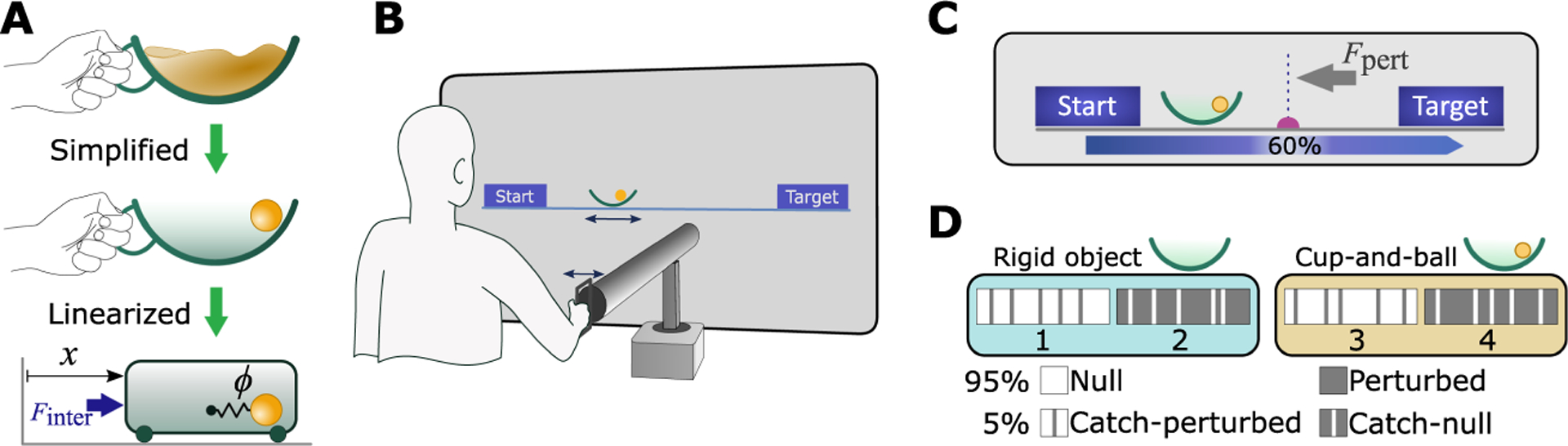
Experimental apparatus and task design. (A) Inspired by the daily activity of carrying a cup of coffee, the simplified model of a cup with a ball inside mimicked the internal dynamics of this complex object. The dynamics of this simplified cup-and-ball system was generated by linearizing the equations of motion of an equivalent cart-and-pendulum system, where the cart was directly driven by the interaction force, $F_{\text{inter}}$, applied by the hand to the cart. (B) The linearized cup-and-ball system was implemented in a virtual environment, although still displayed as a 2D semicircular cup with a ball (bob of the pendulum) sliding inside. Participants could directly control the displayed cup via a robotic manipulandum; they received online haptic feedback about the dynamics of the system. The task involved moving the cup on a horizontal line from the start to the target box (32.5 cm distance) without losing the ball. (C) To enhance the control demands, a perturbation was applied to the cup at 60% of the total path length. The perturbation was an impulse-like force of 20ms duration and 20N magnitude, applied in the opposite direction of the cup movement. The location of the perturbation was visually displayed as a “speed bump,” but did not propel the cup in the vertical direction. (D) Experimental design: Four experimental conditions were tested in four blocks, each with 100 trials: in blocks 1 and 2, subjects interacted with the rigid object (ball fixed to the cup); in blocks 3 and 4 , the full dynamics of the cup-and-ball system was presented. Blocks 1 and 3 contained 95% unperturbed null trials and 5% randomly interspersed perturbation trials, acting as catch trials. Blocks 2 and 4 presented 95% perturbation trials, with 5% unperturbed trials as random catch trials. All catch trials were visually indistinguishable from the rest of the trials in the same block.

The experimental task was inspired by the daily activity of carrying a cup of coffee. Unlike transporting a rigid object, carrying an object with internal degrees of freedom creates nonlinear and potentially even chaotic interaction forces onto the hand (Mayer & Krechetnikov, 2012; Nasseroleslami et al., 2014; Han, 2016; Bazzi & Sternad, 2020b). To examine such interactions, in previous work we simplified the cup of coffee to a 2D semicircular cup with a ball sliding inside and moving on a horizontal line. Despite its simplicity, this model maintained the key elements of underactuation while providing mathematical tractability and virtual implementation of the task (see Figure 1A). This original cup-and-ball system was equivalent to a 2D cart-and-pendulum system, where the bob of the suspended pendulum corresponded to the ball and the 2D arc of the cup was the circular path of the pendular bob.

This study employed optimal feedback control (OFC; Todorov, 2005; see section 2.2.1) as a framework for the controller to explore the contributions of impedance, optimality criteria, and sensory feedback. As OFC was developed for linear systems, the equations of motion of the cup-and-ball system were linearized around the ball’s rest position at the bottom of the cup. This linearization led to the following equations of motion:

(2.1) $$(M+m)\ddot{x}\;=-ml\ddot{\phi }+F_{\text{inter }}+F_{\text{pert }}$$

(2.2) $$l\ddot{\phi }=-g\phi -G\ddot{x}$$

where x and ϕ are the cup position and ball angle, respectively. $F_{\text{inter}}$ is the force of the hand interacting with the cup, and $F_{\text{pert}}$ is an external perturbation force. The system parameters M=3kg and m=0.3kg are the cup and ball masses, respectively. The pendulum length l=0.5m corresponds to the radius of the cup; $g=9.81\;m/s^{2}$ is the gravitational acceleration. G=5 is the coupling term between the cup and the ball dynamics. Since the physical limitations of the robot did not allow simulating arbitrary object parameters, the value of G was set to be greater than 1 to make the ball more agile and responsive to cup accelerations while satisfying the constraints of the robot.

This linearized model was used in both the virtual implementation for the human experiments, as well as in the simulations of different control models. While the linearized model no longer exhibited chaotic behavior, it still presented considerable challenges due to the underactuated degrees of freedom. A previous study demonstrated that the linearized version of the cup-and-ball system demanded slightly higher interaction forces as well as contained a more prohibitive antiresonance frequency, while other control challenges were similar to the nonlinear system (Sohn et al., 2020). Hence, the linearization was not expected to affect the purpose of this study.

Despite linearization, the virtual environment displayed the cup and ball as in previous studies; the angle of the ball in the cup corresponded to the linear state ϕ in equation 2.2. As a control condition for the cup-and-ball system, the internal degree of freedom of the system (i.e., the ball) was constrained by fixing it to the bottom of the cup. This rigid-object condition essentially reduced the behavior to an unconstrained reaching movement with an increased hand mass. The inertia of this rigid object was matched to equate the sum of the cup and ball masses in the non-rigid system. The rigid object was displayed as an empty cup for visual consistency; however, its inertia matched the total mass of the cup and ball.

#### 2.1.3 Experimental Apparatus.

For both object types—rigid object and cup-and-ball—the dynamics were simulated and rendered in a virtual environment, where participants interacted with the object via a haptic robotic manipulandum (HapticMaster, FCS Control Systems, The Netherlands; Van der Linde & Lammertse, 2003). Subjects received haptic feedback about the object dynamics and interaction forces via the robotic handle (see Figure 1B). The robot was admittance controlled: the measured interaction force applied by the subjects on the robot handle determined its acceleration by rearranging the cup’s equation of motion, equation 2.1, as

(2.3) $$a_{\text{robot}\;}=\frac{1}{M+m}\left(F_{\text{inter}\;}+F_{\text{ball }}+F_{\text{pert}\;}\right)$$

with $F_{\text{ball}}=-ml\ddot{\phi }$. The desired acceleration of the robot in equation 2.3 was enforced by its internal controller running at $4\;kHz.\;F_{\text{ball}}$ was updated at a median sampling rate of 641 Hz (interquartile range 500 – 709 Hz) using the actual acceleration of the robot and the ball’s equation of motion, equation 2.2. The measured interaction force as well as the kinematics of the movements were read out from the robot at the same rate of 641 Hz.

The visual information about the object movement and the virtual environment—the target boxes and the added perturbation bump (see details below)—were displayed on a vertical projection screen with a frame rate of 60 Hz. The total latency of the robotic interface and the visual projection, defined as the time difference between the moment the robot’s handle moves until the time the movement is displayed on the screen, was 33ms±17ms. To measure this latency, a single video camera was used to simultaneously capture the robot’s handle and the displayed cup position. This video was processed offline to digitize the positions of the robot and the cup, and the time lags between the two position signals were calculated by cross-correlation.

#### 2.1.4 Experimental Design and Procedure.

Participants stood in front of the projection screen and grasped the handle of the robotic manipulandum by which they could control the displayed object on the screen (see Figure 1B). They were instructed to move the object via the robot’s handle along a horizontal line from a starting box (left) to a target box (right) displayed on the screen. The physical distance between the centers of start and target box was 32.5 cm. Each cup-and-ball trial started with the ball resting at the bottom of the cup. If the ball angle surpassed the rim of the cup during the movement, $\phi >\pm 45^{\circ }$, the ball would “escape,” and the trial would be terminated as a failed attempt. The failed trials were not repeated. A successful trial ended when the cup was fully inside the target box and its speed fell below 5mm/s. To avoid lengthy adjustments to home into the target, the start and target boxes were 30% wider than the width of the cup, allowing ±5.3mm tolerance. For the rigid-object condition, there were no failures; all trials were considered successful.

To promote consistency across subjects, participants were instructed via feedback to finish each trial within 1.4s(±20%) for the rigid object and 1.8s(±20%) for the cup-and-ball system. These movement times were chosen from preferred durations measured in pilot experiments. The movement durations were displayed at the end of each trial with the text color indicating whether the duration was within the acceptable bound (green), too fast (yellow), or too slow (red). This feedback was merely informative, and violation of the desired movement time did not lead to any explicit penalties or exclusion of the trial from analysis. Overall, subjects very quickly learned and adhered to the suggested movement time (see section 3.1).

In many trials, an external perturbation was applied that disturbed the movements (see Figure 1C). The perturbation was an impulsive force of magnitude $F_{\text{pert}}=20\;N$, applied to the cup for 20ms in the opposite direction of the movement, without any effect in the vertical direction. This perturbation was applied at a fixed location 60% into the movement distance toward the target. With the exception of the catch trials (see below), the existence of the perturbation in a trial was cued using a visual speed bump (see Figure 1C). Therefore, the participants were aware of its presence or absence before the trial began.

Each participant performed the task with both the rigid object and the cup-and-ball system in a total of 4 blocks of 100 trials each (see Figure 1D). The first 2 blocks involved performing the task with the rigid object, and blocks 3 and 4 presented the cup-and-ball system. Block 1 consisted of 95% unperturbed (null) trials and 5% perturbation trials (catch-perturbed), randomly interspersed across the block. Subjects were unaware of the perturbation prior to the trial onset, and there was no visual cue signaling the perturbation. Block 2 consisted of 95% perturbed trials (speed bump shown) and 5% null trials (catch-null), randomly interspersed across the block. Unlike the catch-perturbed trials in block 1, the catch-null trials in block 2 were also cued with a visually displayed speed bump on the way to the target like the remaining 95% of the trials in that block. Hence, subjects expected a perturbation, but no force was applied. Blocks 3 and 4 were the same as blocks 1 and 2, respectively, except that subjects interacted with the cup-and-ball system. The order of the blocks was the same for all participants. Overall, each participant performed 400 trials. Each block lasted approximately 10 to 12 minutes, with sufficient rest time given between the blocks. The experiment lasted approximately 1 hour from beginning to end.

#### 2.1.5 Dependent Measures and Statistical Analysis of the Experimental Data.

A first analysis aimed to assess whether there were any improvements during each of the four blocks. This was particularly relevant in the cup-and-ball condition that posed more coordination challenges. Using movement time as an indicator, this was determined as follows: trial onset was defined as the time when the cup velocity exceeded 5mm/s in direction of the target; the trial ended when the cup was fully inside the target box and its speed fell below 5mm/s To evaluate whether movement time changed over the course of practice, a Wilcoxon signed-rank test (within-subject, N=11) compared the median trial duration of the first 25 and last 25 trials of each block. This nonparametric test was appropriate as the distributions of this variable did not meet the normality criterion.

To evaluate how subjects coped with the expected perturbations in the perturbed trials, the interaction forces were examined. Inspection of the trials in both the rigid-object and cup-and-ball trials showed that the force exhibited a characteristic discontinuity immediately following the perturbations (see section 3). To quantify this observed phenomenon in the different conditions, the force value at the onset of the perturbation was compared against its value immediately after the 20ms interval of the perturbation. For each participant, the difference values of $F_{\text{pre}}-F_{\text{post}}$ were tested against zero using unpaired t-tests. These tests were conducted separately for the rigid-object and the cup-and-ball conditions.

The cup velocity in the cup-and-ball condition rebounded after the perturbation. In the data, this rebound started immediately after the end of the perturbation. To quantify this feature, the cup’s peak acceleration following the perturbation was identified in each trial. The peak values and the time of the peak relative to the end of the perturbation were analyzed.

### 2.2 Control Models.

**Figure 2:**
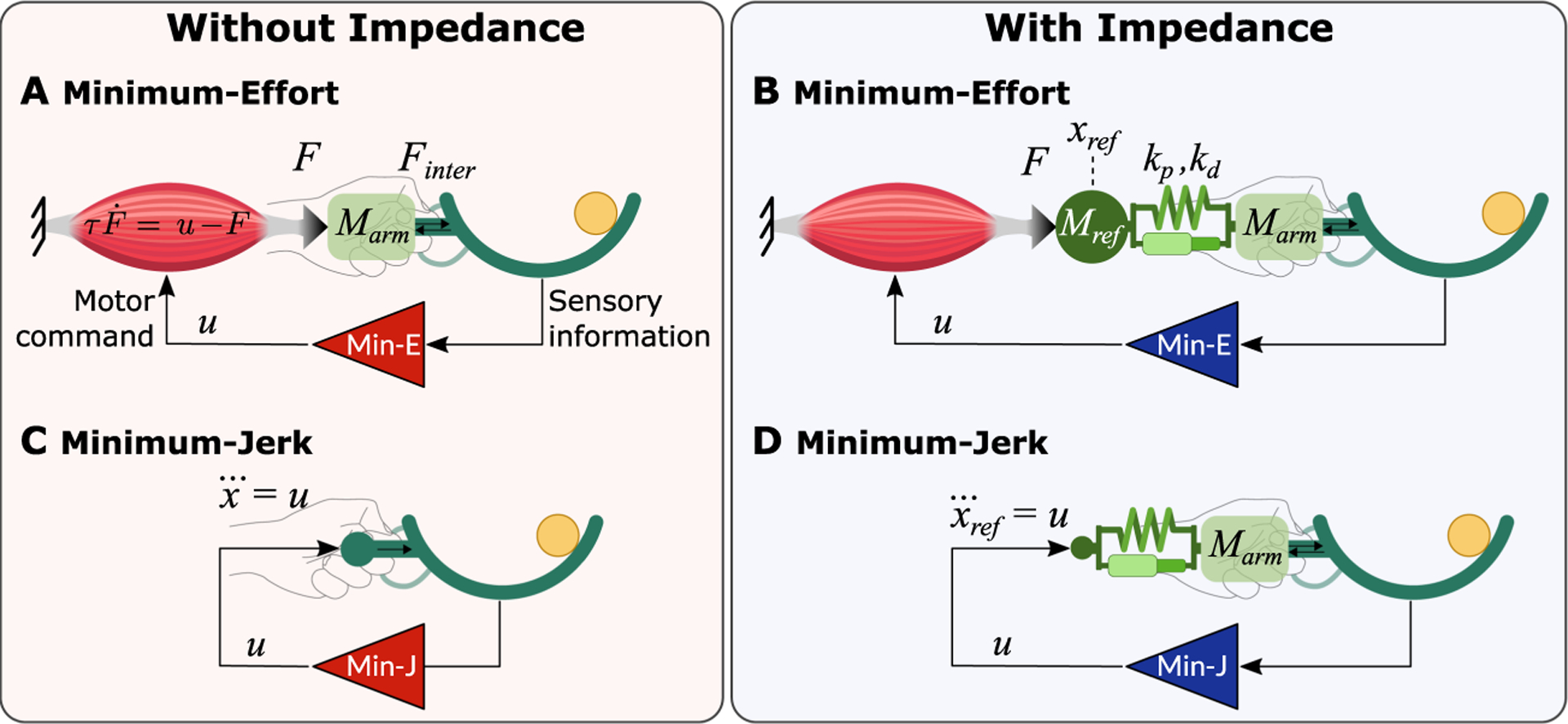
(A) The basic OFC model included a simplified muscle model. This muscle model produced force F according to the motor command u; minimizing u was a proxy for minimizing neuromuscular effort. The interaction force was measured at the interface between the hand mass $M_{\text{arm}}$ and the object. (B) The second model included an impedance element (stiffness and damping $k_{p},k_{d}$) and an inertia, $M_{ref}$, that was placed between the muscle model and the hand/object. The inertial dynamics of $M_{ref}$ produced the reference trajectory $x_{\text{ref}}$ under the applied forces. (C) The minimum-jerk variant of the OFC model did not include a muscle model; instead, the controller output the jerk of the cup trajectory. Thus, minimizing u resulted in a minimum-jerk trajectory that took into account the dynamics of the object. (D) With impedance in the model, the minimum-jerk variant of the OFC model prescribed the kinematics of the reference trajectory for the impedance elements.

To identify the necessary elements in a control model for this task, several models were developed and compared with the experimental behavior. All models were based on the optimal feedback control framework as presented by Todorov (2005). To simulate the experimental data, the control models needed to successfully transport the cup-and-ball to the target without losing the ball. Two basic control models were developed (see Figure 2): The first model included the dynamics of the object, cup-and-ball and also the rigid object, together with a simplified inertial and muscular dynamics of the arm (see Figure 2A, section 2.2.2). This basic OFC model was contrasted against a second model that further included a simplified mechanical impedance element (see Figure 2B, section 2.2.3). This constant impedance stood as proxy for the compliant dynamics.of the arm. These two models served as the basis to study the interplay of impedance, cost functions, and corrections via sensory feedback.

The starting hypothesis was that arm impedance critically affected the model behavior, especially when additional external perturbations occurred. The two basic model structures compared the simulated behavior with both the rigid and the cup-and-ball object. These models included the most frequently used cost function that minimized effort (Todorov & Jordan, 2002b). The second point of interest was the effect of the cost function on behavior once impedance was included. To address this question, the two OFC models compared the behavior using the cost functions’ minimum effort with minimum jerk (see Figures 2C and 2D, sections 2.2.4 and 2.2.5). The third analysis evaluated the role of sensory feedback, especially when facing unexpected external perturbations. This analysis focused on the catch trials and compared the basic feedback control model with a variant that eliminated feedback.

#### 2.2.1 Basics of Stochastic Optimal Feedback Control.

The optimal feedback control (OFC) model that had been frequently used in movement neuroscience represented the neural controller as a linear quadratic gaussian controller that dealt with additive and multiplicative sensory and motor noise (Todorov, 2005). This optimal controller determined the control command u that minimized the quadratic cost function

(2.4) $$J=\sum_{t=0}^{N-1}\;\left(x_{t}^{T}Q_{t}x_{t}+u_{t}^{T}R_{t}u_{t}\right)+x_{N}^{T}Q_{N}x_{N}$$

subject to

(2.5) $$\text{ Dynamics: }x_{t+1}=Ax_{t}+B\left(I+\varepsilon_{t}\right)u_{t}+\xi_{t}\;\text{ Sensory feedback: }y_{t}=H\left(I+\epsilon_{t}\right)x_{t}+\omega_{t}$$

where x is the state vector and ξ and ε represent additive and multiplicative (control-dependent) motor noise terms, respectively. The subscript t represents the time step, and *N* is the total number of time steps in the simulations. The terms $R_{t}$ and $Q_{t}$ in the objective function, equation 2.4, are weights for effort and accuracy costs, respectively. For all simulations, we used $R_{t}=1$ for all $t.\;Q_{t}$ was separately defined for each model, depending on the number of states in that model (see below). The sensory feedback $y_{t}$ contained additive ω and multiplicative ϵ sensory noise. The optimal control law was then calculated as a feedback gain $u_{t}=L^{*}\overset{ˆ}{x}$, where the estimated state vector $\overset{ˆ}{x}$ was calculated based on the delayed feedback signal and the system dynamics (see Todorov, 2005, for details on the implementation of sensory delay and the calculation of feedback gains). For our simulations, the sensory delay was set to 50ms. The motor noise terms ξ and ε were zero mean gaussian noise with the standard deviations of 10−^4^ and 1 , respectively (with appropriate SI units). The covariance matrix for the additive sensory noise was a diagonal matrix defined as $\omega =\text{diag}\;\left(10^{-5}\right)$, and no state dependency was considered for the sensory noise (ϵ=0; see Table 1 for a full description of the parameter values). The following sections present each model variant with its state vector and dynamic properties (matrices A,B, and H in equations 2.5).

#### 2.2.2 Minimum Effort Model without Impedance.

The conventional OFC model is often formulated to minimize the square of neuromuscular effort (Todorov & Jordan, 2002b; Diedrichsen et al., 2010). To include a proxy for neuromuscular dynamics in this model, the equations of motion of the object, equation 2.1, were coupled with a first-order muscle model (Crevecoeur et al., 2019) as shown in Figure 2A,

(2.6) $$\left(M_{\text{arm }}+M+m\right)\ddot{x}\;=-ml\ddot{\phi }+F+F_{\text{pert }}\;l\ddot{\phi }\;=-g\phi -G\ddot{x}\;\tau \dot{F}\;=u-F$$

where F is the muscle output force applied to the hand $M_{\text{arm}}$ and indirectly to the cup-and-ball (see Figure 2A). Note that the interaction force $F_{\text{inter}}$ in equation 2.1 was not the same as the muscle output force F, as the latter needed to be calculated between the hand and the cup-and-ball system. To be consistent with the experimentally measured values, the interaction force was calculated from equation 2.1 given the simulated x and ϕ trajectories.

The perturbation force $F_{\text{pert}}$ was an impulse force that acted on the cupand-ball system for a short time (20ms) at the time the cup traveled 60% of the target distance. For null trials, $F_{\text{pert}}=0$ at all times. The numeric values of system parameters in the simulations were M=3kg,m=0.3kg, $l=\;0.5\;m,\;g=9.81\;m/s^{2},$ G=5,and τ=30ms. The hand mass $M_{\text{arm}}$ was a free parameter in the model-fitting procedures (see section 2.3). Given the equations of motion, the model could be written in the standard state-space form as follows:

(2.7) $$x\;={\left[x,\phi ,\dot{x},\dot{\phi },F,F_{\text{pert }}\right]}^{T}\;A\;=\left[\begin{array}{cccccc} 0 & 0 & 1 & 0 & 0 & 0\\ 0 & 0 & 0 & 1 & 0 & 0\\ 0 & \frac{mg}{\alpha } & 0 & 0 & 1/\alpha & 1/\alpha \\ 0 & \frac{-g}{l}\left(1+\frac{Gm}{\alpha }\right) & 0 & 0 & \frac{-G}{l\alpha } & \frac{-G}{l\alpha }\\ 0 & 0 & 0 & 0 & -1/\tau & 0\\ 0 & 0 & 0 & 0 & 0 & 0 \end{array}\right]\;B\;=[0,0,0,0,1/\tau ,0{]}^{T}\;H\;=I_{6\times 6}$$

where $\alpha =m+\left(M+M_{\text{arm}}\right)-mG$.

In blocks 2 and 4, participants anticipated the impulsive perturbation. This anticipation also had to be modeled within the OFC framework. Note that the optimal controller’s behavior is only influenced by the state-space matrices A,B, and H, as well as Q. Therefore, the perturbation needed to be included as a state to embed such information in matrix **A**. Further note that the position-dependent perturbation, as in the experiments, could not be modeled in a linear state-space form; instead, the perturbation was implemented as time-dependent, and its timing was manually adjusted so that the perturbation occurred at the right position in the simulations. To implement this perturbation, the zero-dynamic state variable $F_{\text{pert}}$ was set to −20N, but its corresponding terms $A_{3,6}$ and $A_{4,6}$ were zero when the perturbation force was inactive. These state-space equations were subsequently time-discretized using Euler integration with time-step δt=10ms to be used in the discrete-time optimal control problem of equations 2.4 and 2.5. Following time discretization, the time-dependent matrix $Q_{t}$ was defined as

(2.8) $$Q_{t}=\left\{\begin{array}{ll} Diag\;\left(\left[p_{x},p_{b},p_{\dot{x}},0,0,0\right]\right) & N-50<t\leq N\\ Diag\;\left(\left[0,p_{b},0,0,0,0\right]\right) & t\leq N-50 \end{array}\right.$$

with N denoting the total number of time steps. Note that the cost term $Q_{t}$ penalized the cup position (x) and cup velocity $\left(\dot{x}\right)$ in the last 50 time steps (=500ms) of the movement to ensure that the object came to rest at the target. The parameters $p_{x}$ and $p_{\dot{x}}$ in the $Q_{t}$ matrix were the penalty values assigned to the cup position and velocity ($p_{x}=p_{\dot{x}}$ in the simulations). Further, to prevent the ball from escaping during the simulations, the penalty for the ball angle $p_{b}$ held for the entire movement duration. These free penalty parameters were obtained by fitting the models to the experimental data. The initial conditions were $x_{0}=[-32.5\;cm,0,0,0,0,-20\;N{]}^{T}$ and the total simulation time was set to the average participant’s trial duration, plus 500ms hold time.

#### 2.2.3 Minimum Effort Model with Impedance.

In human motor control, mechanical impedance of the body has often been approximated by linear spring and damper elements. Figure 2B illustrates how the model of the arm was extended by an impedance. By adding a linear spring $k_{p}$ and damper $k_{d}$ to the system, the equations of motion took the form

(2.9) $$\left(M_{\text{arm }}+M+m\right)\ddot{x}\;=-ml\ddot{\phi }+k_{p}\left(x_{ref}-x\right)+k_{d}\left({\dot{x}}_{ref}-\dot{x}\right)+F_{\text{pert }}\;l\ddot{\phi }\;=-g\phi -G\ddot{x}\;\tau \dot{F}\;=u-F\;M_{ref}{\ddot{x}}_{\text{ref }}\;=k_{p}\left(x-x_{\text{ref }}\right)+k_{d}\left(\dot{x}-{\dot{x}}_{ref}\right)+F$$

where $x_{ref}$ represents the reference trajectory for the spring and damper system, the point where the muscle force F is applied (see Figure 2B). Note that the muscle force in this case could only indirectly affect the cup-and-ball dynamics via the impedance operator. The nonzero mass $M_{ref}$ in the equations was needed to avoid a mathematical singularity (division by zero) when modeling the system. This point mass can be considered as a lumpedparameter effective mass of the engaged musculature. In this model, the parameters $M_{arm},M_{ref},k_{p}$, and $k_{d}$ were free parameters found during the model-fitting procedure. The standard state-space representation of the model was

(2.10) $$x\;={\left[x,\phi ,\dot{x},\dot{\phi },F,x_{ref},{\dot{x}}_{ref},F_{\text{pert }}\right]}^{T}\;A\;=\left[\begin{array}{cccccccc} 0 & 0 & 1 & 0 & 0 & 0 & 0 & 0\\ 0 & 0 & 0 & 1 & 0 & 0 & 0 & 0\\ \frac{-k_{p}}{\alpha } & \frac{mg}{\alpha } & \frac{-k_{d}}{\alpha } & 0 & 0 & \frac{k_{p}}{\alpha } & \frac{k_{d}}{\alpha } & \frac{1}{\alpha }\\ \frac{k_{p}G}{l\alpha } & \frac{-g}{l}\left(1+\frac{Gm}{\alpha }\right) & \frac{k_{d}G}{l\alpha } & 0 & 0 & \frac{-k_{p}G}{l\alpha } & \frac{-k_{d}G}{l\alpha } & \frac{-G}{l\alpha }\\ 0 & 0 & 0 & 0 & -1/\tau & 0 & 0 & 0\\ 0 & 0 & 0 & 0 & 0 & 0 & 1 & 0\\ \frac{k_{p}}{M_{ref}} & 0 & \frac{k_{d}}{M_{rf}} & 0 & 1 & \frac{-k_{p}}{M_{rf}} & \frac{-k_{d}}{M_{ref}} & 0\\ 0 & 0 & 0 & 0 & 0 & 0 & 0 & 0 \end{array}\right]\;B\;=\left[\begin{array}{ccccc} 0,0,0,0, & 1/\tau ,0,0,0{]}^{T} & & & \end{array}\right.\;H\;=I_{8\times 8}$$

The cost term $Q_{t}$ was defined as an 8×8 diagonal matrix, with penalty terms considered for the cup position and velocity, and the ball angle, as shown in equation 2.8.

#### 2.2.4 Minimum Jerk Model without Impedance.

To study the effects of the objective function in our task, the objective of minimizing effort was contrasted with the objective of maximizing kinematic smoothness. Kinematic smoothness, frequently quantified by the time derivative of acceleration (jerk), is another widely discussed objective function for human movements (Flash & Hogan, 1985; Dingwell et al., 2004; Svinin et al., 2006, 2019). It must be noted that forces from the ball made the cup trajectory deviate from a pure minimum-jerk profile, i.e., it no longer exhibited a bell-shaped maximally smooth velocity profile (Bazzi & Sternad, 2020a). However, the OFC framework could include other cost terms, such as a penalty on the movement of the ball, to minimize jerk while taking into account the dynamics of the task.

To minimize cup jerk in the model, the equations of motion had to be rewritten to have the cup jerk as either an input or a state. This modification was required to use the quadratic cost function, equation 2.4, to minimize the squared value of jerk. In this model, the optimal feedback controller specified the third derivative of the cup position as the control input $\left(\frac{d}{dt}(\ddot{x})=u\right.$; see Figure 2C). To incorporate this equation into the statespace model, the cup acceleration $\ddot{x}$ was included as an additional state. Note that u directly prescribed the cup kinematics: the cup movement was forced. Therefore, the muscle model no longer had an effect on the overall system dynamics and was removed. Following this modification, the equations of motion became

(2.11) $$\dddot{x}=u\;l\ddot{\phi }=-g\phi -G\ddot{x}$$

Further, because the cup kinematics were fully prescribed by the controller, the perturbation force $F_{\text{pert}}$ did not affect the cup movement and was removed from the equations. Therefore, the state-space representation of the dynamics became

(2.12) $$x\;=[x,\phi ,\dot{x},\dot{\phi },\ddot{x}{]}^{T}\;A\;=\left[\begin{array}{ccccc} 0 & 0 & 1 & 0 & 0\\ 0 & 0 & 0 & 1 & 0\\ 0 & 0 & 0 & 0 & 1\\ 0 & \frac{-g}{l} & 0 & 0 & \frac{-G}{l}\\ 0 & 0 & 0 & 0 & 0 \end{array}\right]\;B\;=[0,0,0,0,1{]}^{T}\;H\;=I_{5\times 5}$$

As the control command u in this OFC variant was directly defined as the jerk of cup movement, the penalty term $u_{t}^{T}R_{t}u_{t}$ in the cost function, equation 2.4, led to a minimum jerk trajectory, while taking into account the dynamics of the ball and the requirements of the task. For this model, the $Q_{t}$ matrix was defined as a 5×5 diagonal matrix with the same ball and cup penalty terms as in equation 2.8.

#### 2.2.5 Minimum-Jerk Model with Impedance.

For the minimum-jerk variant of the model that includes impedance (see Figure 2D), the third derivative of the reference trajectory was prescribed as the control input, $\frac{d}{dt}\left({\ddot{x}}_{ref}\right)=u$. Note that if the jerk of the cup trajectory was minimized, the resulting movement would be the same as the minimum-jerk OFC model without impedance.Therefore, the acceleration of the reference trajectory was included as a state in the state vector:

(2.13) $$(M_{arm}+M+m)\ddot{x}=-ml\ddot{\phi }+k_{p}(x_{ref}-x) +k_{d}({\dot{x}}_{ref}-\dot{x})+F_{\text{pert}}\;l\ddot{\phi }=-g\phi -G\ddot{x}\;{\dddot{x}}_{ref}=u$$

which was written in a state-space form as

(2.14) $$x\;={\left[x,\phi ,\dot{x},\dot{\phi },x_{ref},{\dot{x}}_{ref},{\ddot{x}}_{ref},F_{\text{pert }}\right]}^{T}\;A\;=\left[\begin{array}{cccccccc} 0 & 0 & 1 & 0 & 0 & 0 & 0 & 0\\ 0 & 0 & 0 & 1 & 0 & 0 & 0 & 0\\ \frac{-k_{p}}{\alpha } & \frac{mg}{\alpha } & \frac{-k_{d}}{\alpha } & 0 & \frac{k_{p}}{\alpha } & \frac{k_{d}}{\alpha } & 0 & \frac{1}{\alpha }\\ \frac{k_{p}G}{l\alpha } & \frac{-g}{l}\left(1+\frac{Gm}{\alpha }\right) & \frac{k_{d}G}{l\alpha } & 0 & \frac{-k_{p}G}{l\alpha } & \frac{-k_{d}G}{l\alpha } & 0 & \frac{-G}{l\alpha }\\ 0 & 0 & 0 & 0 & 0 & 1 & 0 & 0\\ 0 & 0 & 0 & 0 & 0 & 0 & 1 & 0\\ 0 & 0 & 0 & 0 & 0 & 0 & 0 & 0\\ 0 & 0 & 0 & 0 & 0 & 0 & 0 & 0 \end{array}\right]\;B\;={\left[\begin{array}{cc} 0,0,0,0,0,1, & 0 \end{array}\right]}^{T}\;H\;=I_{8\times 8}$$

In both minimum-jerk variants (equations 2.12 and 2.14), the state penalties $Q_{t}$ were defined similarly as in equation 2.8 with penalty terms for cup position and velocity, as well as the ball angle.

#### 2.2.6 Simulating Movements with a Rigid Object.

All four models described in equations 2.7, 2.10, 2.12, and 2.14, were developed based on the cup-and-ball system. To simulate the movements with the rigid object, the states regarding the ball movement (ϕ and $\dot{\phi })$ were removed from the state vector, as well as the corresponding rows and columns from the matrices A,B,H, and Q.

#### 2.2.7 Simulating Null, Perturbed, and Catch Trials.

The experimental design tested four different trial types: null, perturbation, catch-null, and catch-perturbed. For the perturbation trials and also the null trials, prior to the onset of movement, a visual cue (the speed bump or the absence of a bump) informed the subjects of the presence or absence of a perturbation. To incorporate this “knowledge” in the simulations, the perturbation force $F_{\text{pert}}$ was included as a state in the state-space equations; by initializing simulations with $F_{\text{pert}}=-20\;N$, the knowledge about the time-dependent dynamics of the perturbation was already incorporated in the control system via the time-dependent A matrix. For the null trials, the perturbation state $F_{\text{pert}}$ was set to zero. In this case, the perturbation was neither expected by the controller, nor did it occur.

**Figure 3:**
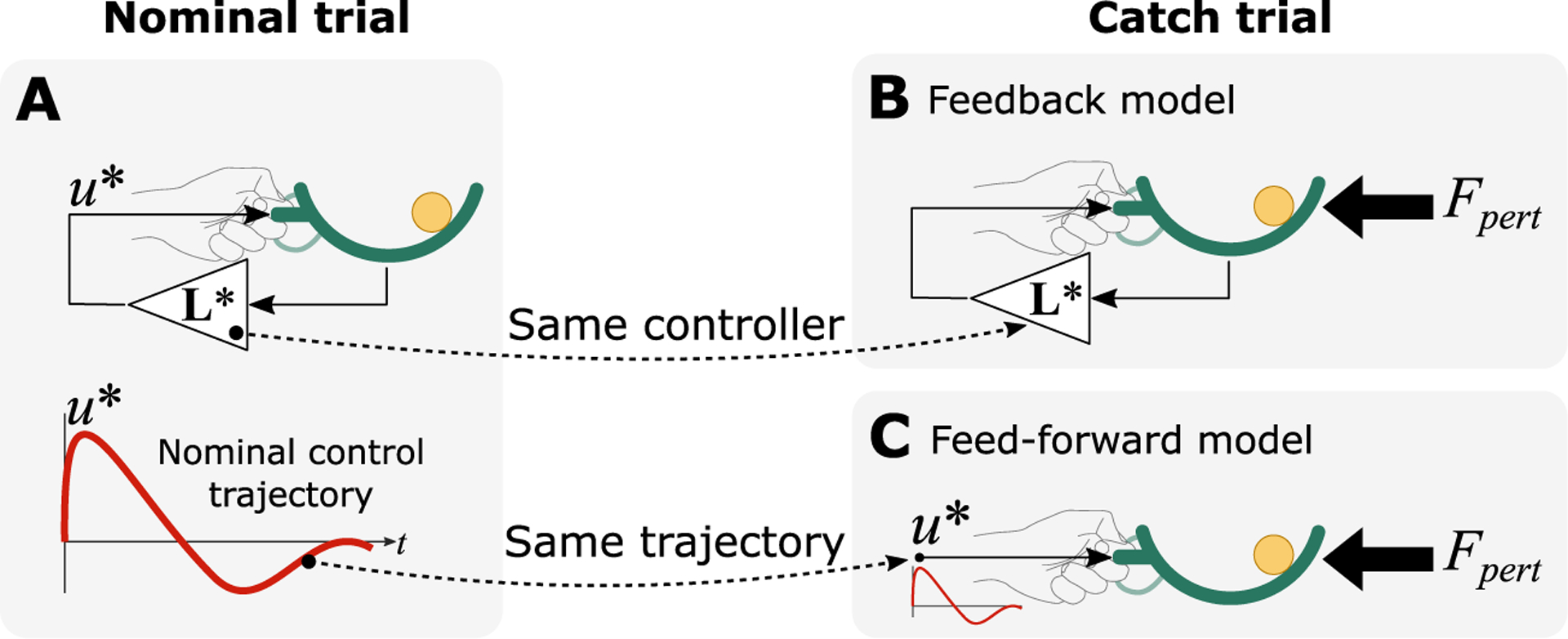
Simulating the catch-perturbed condition. (A) In the nominal condition without perturbation, an optimal control model was created, which expected no perturbation. (B) To simulate the catch-perturbed trial, the same controller $L^{*}$ that was unaware of the perturbation was used to simulate a trial that faced a perturbation (the feedback-driven model). (C) Only the nominal control trajectory $u^{*}$ was supplied to the perturbed simulation in the feedforward model. The inverse procedure was used for catch-null trials, where a perturbation-aware controller was used in a trial without perturbation.

For the catch trials, the simulations were performed in a two-step procedure (see Figure 3). For catch-perturbed trials, a movement was first simulated as a null trial, and then the optimal control gains were obtained $\left(L^{*}\right.$ in $u=L^{*}\overset{ˆ}{x}$; see Figure 3A; see also Todorov, 2005). In the second step, the movement was simulated based on the dynamics of a perturbed trial, even though the control gains were obtained from the null trial in the previous step (see Figure 3B). In this case, the controller was not prepared for a perturbation, but a perturbation occurred. Conversely, for the catch-null trials, the optimal control gains were first obtained by simulating a perturbed trial, and then the same control gains were used to simulate a movement based on the dynamics of a null trial. Here, the knowledge about the perturbation was incorporated in calculating the control gain, but the perturbation did not occur.

#### 2.2.8 Simulating Feedforward Control.

For the feedforward simulations, the control command u was first obtained from the optimal feedback control applied to a given nominal condition (e.g., for a null trial, Figure 3A), and then replayed in a feedforward manner. This controller was specifically used to simulate the catch conditions to assess how the control command without sensory feedback could compensate for unexpected perturbations.

### 2.3 Model Fitting.

Each control model contained free parameters that were used to fit the model predictions to experimental data. The free parameters for each model and their lower and upper bounds are summarized in Table 2. All other parameters, such as for the noise terms, were fixed across all models (see Table 1).

To fit each model, an offline nonlinear optimization algorithm searched for the parameter values that minimized the overall error of the fit (see below) across all four experimental blocks. The models were fit to the data of each participant separately. This optimization procedure consisted of two stages. In the first stage, a global optimization process was implemented to find appropriate initial values for the parameters to be used in a more refined optimization later. This stage was implemented using the particle swarm optimization algorithm (MATLAB function particleswarm, MATLAB 2021a, Mathworks Inc, Natick, MA) with a maximum of 300 iterations. In the second stage, the obtained parameters were used as a starting point for a nonlinear gradient-based optimization algorithm (sequential quadratic programming, implemented with fmincon in MATLAB 2021a), to converge to the optimal values faster.

### 2.4 Model Evaluation against Experimental Data.

To compare the simulated and experimental trajectories for model fitting, all trajectories were first time-aligned based on the moment when the cup had traveled 60% of the target distance; this coincided with the perturbation onset in the perturbed trials. All experimental trajectories were resampled at 100 Hz to match the sampling rate of simulated trajectories. To evaluate the performance of each model, the root-mean-squared error (RMSE) between model and data was obtained for five variables: position and velocity of the cup, angular position and velocity of the ball, and the interaction force. For each variable, the model prediction was compared to the data in the last 40 trials of each block (to focus on stable performance). The resulting RMSE values were averaged and then normalized as

(2.15) $$e_{□}=\frac{\frac{1}{n}\sum_{i=1}^{n}\sqrt{\frac{1}{N}\sum_{t=0}^{N}{\left({□}_{t}^{i}-{□}_{t}^{\text{sim}}\right)}^{2}}}{\frac{1}{n}\sum_{i=1}^{n}\sqrt{\frac{1}{N-1}\sum_{t=0}^{N-1}{\left({□}_{t}^{i}\right)}^{2}}}$$

where the symbol □ represents the variable of interest $\left(x,\dot{x},\phi ,\dot{\phi }\right.$, and $\left.F_{\text{inter}}\right),\;i$ is the trial number (out of n=40 trials in the block), and t is the time step (with total number of N steps that depended on the participant’s average trial duration). The normalization intended to make the error comparable across different variables. Then the average of these normalized errors across all variables was taken as the single evaluation measure that represented the overall modeling error for a given block and subject:

(2.16) $$e=\frac{1}{5}\left(e_{x}+e_{\dot{x}}+e_{\phi }+e_{\dot{\phi }}+e_{F_{\text{inter}\;}}\right)$$

For the rigid-object condition, $e_{\phi }$ and $e_{\phi }$ were removed.

### 2.5 Statistical Analysis of the Modeling Results.

The overall modeling error in equation 2.16 was used to statistically compare the selected models. The goal was to evaluate whether the model variants resulted in statistically different predictions quantified by the model-fitting errors.

To evaluate the effect of impedance on the model behavior (hypothesis 1), a three-way repeated-measures ANOVA was performed. The three factors were model type (with versus without impedance), object type (rigid object versus cup-and-ball), and perturbation type (null versus perturbed). The next analysis on the effects of the cost function applied a four-way repeated-measures ANOVA (hypothesis 2). The factors were model type, object type, and perturbation type as before, as well as the cost function (minimum-effort versus minimum-jerk). Catch trials were excluded for these two analyses. Finally, to assess the effects of feedback on performance, another four-way repeated-measures ANOVA was used with the factor feedback condition (feedback versus feedforward) instead of cost function (hypothesis 3). Only catch trials were used in the last analysis. To disentangle the interactions, post-hoc comparisons with Tukey-Kramer corrections were applied. All statistical tests were performed in MATLAB 2021a, using the ANOVA tools and multcompare function.

As different models had different number of parameters, the Bayesian information criterion (BIC) was applied to each model. BIC evaluates the model performance based on the data-fitting error, while also compensating for the number of free parameters to avoid overfitting (Schwarz, 1978). A BIC difference of greater than 4.6 (a Bayes factor of greater than 10) was considered as strong evidence in favor of the model with the lower BIC value (Kass & Raftery, 1995):

(2.17) $$BIC=nlog\;\left(\frac{e}{n}\right)+klog\;(n)$$

where n is the number of data points, k is the number of free parameters in the model, and e is the performance error calculated in equation 2.16.

## 3 Results

### 3.1 Behavioral Results.

Participants were largely successful in all task conditions. When transporting the cup and ball in blocks 3 and 4, they lost the ball in only 56 trials out of the total of 2200 trials in this condition. The median success rates across participants (i.e., percentage of trials in which the ball was not lost) were 99% and 97% in blocks 3 and 4 , respectively.

To probe whether there were improvements due to practice within each block, trial durations across the 100 trials of each block were examined. The median movement time between the first 25 and last 25 trials of each block were compared using the Wilcoxon signed rank test; failed trials and catch trials were excluded. The median movement time decreased with practice only in block 3 (unperturbed cup-and-ball condition; see Table 3). No significant changes in movement times were observed in other blocks. Note the significantly higher movement times in blocks 3 and 4 when the ball was sliding inside the cup; this observation gave first evidence for the higher demands when transporting the cup with the moving ball.

**Figure 4:**
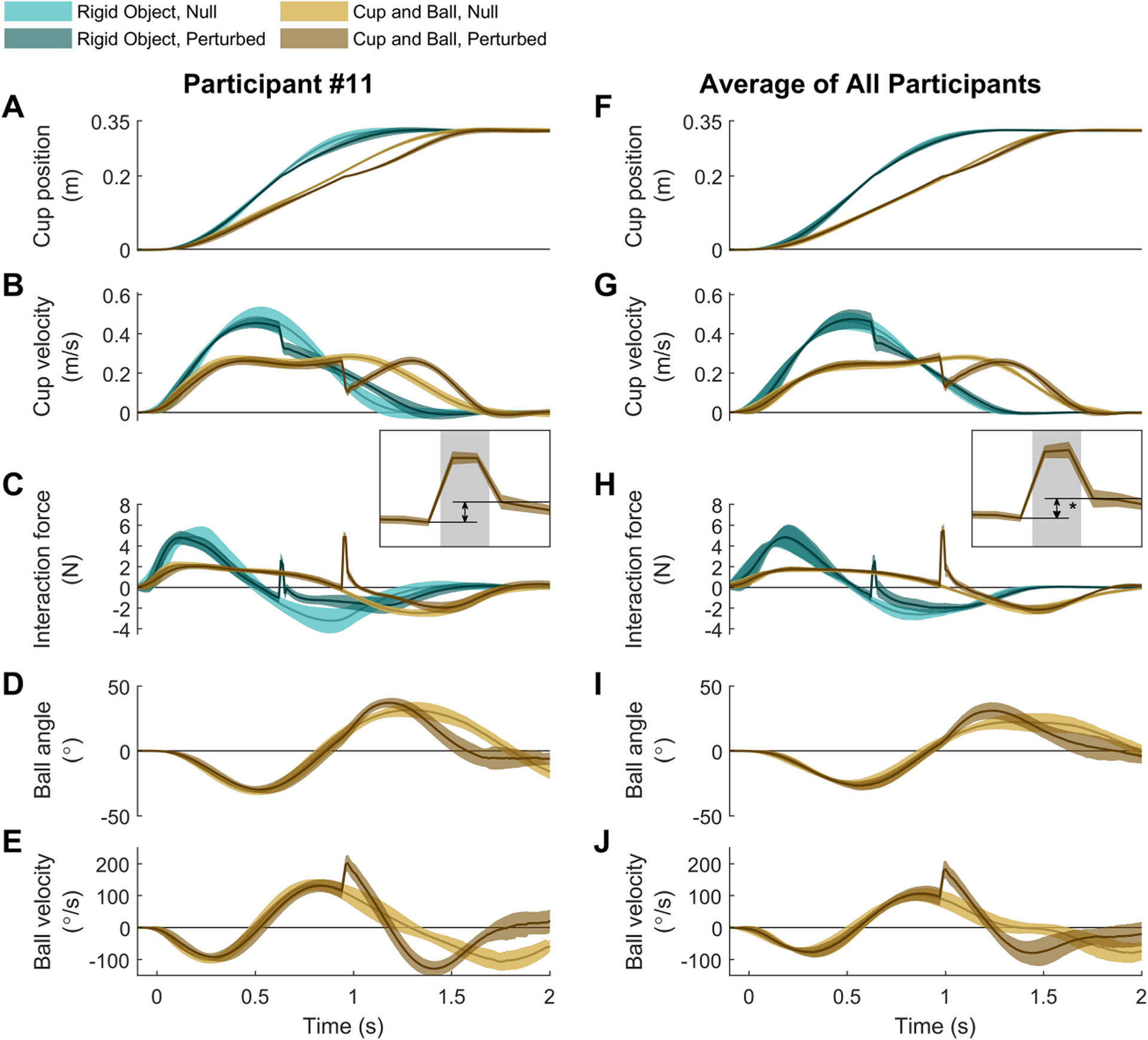
(A-E) Example participant’s trajectories with the rigid object and the cup-and-ball system under free and perturbed conditions. Different color lines show the mean ± 1 standard deviation of the corresponding trajectories across trials for each block (excluding the catch trials). The inset in panel C highlights the detailed features of the interaction force at the perturbation in the cup-and-ball condition. The shaded region is the 20ms interval in which the perturbation was applied. (F-J) Summary of all participants’ trajectories. Mean ± 1 standard deviation of the average trajectories from all participants are shown.

Figure 4 summarizes average trajectories of cup and ball and interaction force in all conditions; catch and failed trials were excluded. Panels A to E show one example participant’s behavior, and panels F to J present averages across all participants. The other individual participants’ data are provided in the supplementary material, Figures S-2 through S-45. As expected, in the rigid object condition of blocks 1 and 2, participants moved the object to the target with kinematics that resembled those seen in free reaching (i.e., a smooth bell-shaped velocity profile; see Figures 4A and 4B, cyan trajectories). With the introduction of the ball dynamics in blocks 3 and 4, new movement profiles emerged that were distinctively different from those in the rigid object condition. Specifically, the cup velocity (see Figure 4B, light brown trajectories) exhibited a plateau, instead of the prominent peak in the rigid object condition. These features were robustly observed across all participants, as indicated by the narrow standard deviation bands in Figures 4F and 4J.

Prior to the onset of the predictable perturbations in blocks 2 and 4, participants’ movement patterns were similar to those in the unperturbed trials, with both the rigid object and the cup-and-ball system (see Figure 4, darker brown trajectories). Until arriving at the perturbation location, participants continued to move with a velocity profile that showed a single prominent peak in the rigid object case and a plateau in the cup-and-ball condition (see Figures 4B and 4G). In both conditions, the impulsive resistive force caused a sudden drop in the cup velocity; in the cup-and-ball condition, the cup velocity rebounded after the perturbation (see Figures 4B and 4G), but this rebound was absent in the rigid-object condition. Further, with both objects, the interaction force showed a prominent spike (see Figures 4C and 4H). The interaction force exhibited a discontinuity at the time of perturbation as highlighted in the zoomed-in insets in Figures 4C and 4H; the interaction force right after the perturbation was consistently larger than its value at the onset of perturbation as the inset highlights. Averaged across participants, this increase was 0.99±0.72N with the rigid object $\left(t(10)=4.553,p=1.05\times 10^{-3}\right)$ and 1.52±0.69N with the cup-and-ball system $\left(t(10)=7.323,p=2.53\times 10^{-5}\right)$.

### 3.2. Modeling the Behavior.

#### 3.2.1 The Effects of Impedance.

**Figure 5:**
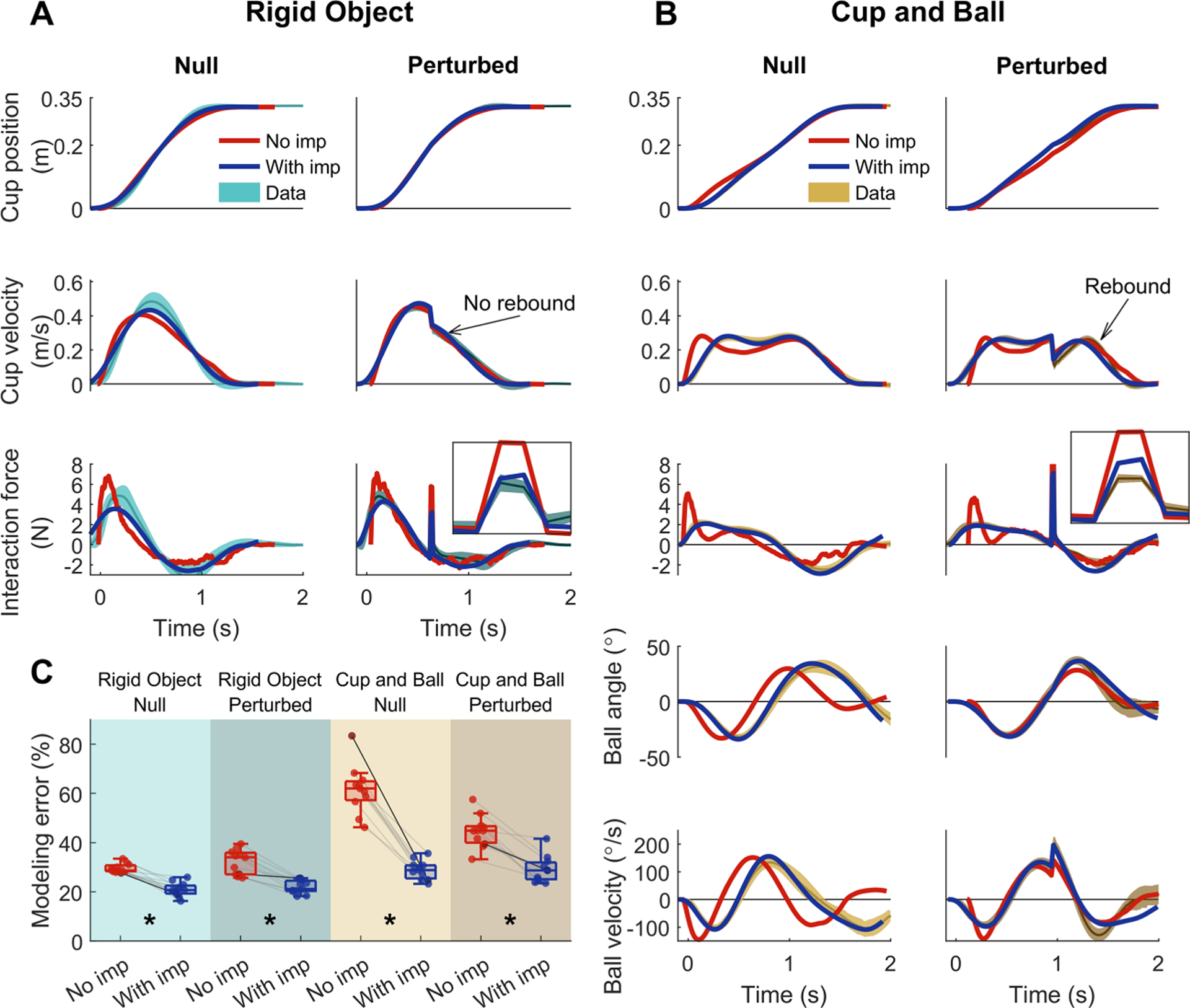
Comparison of the responses of the minimum-effort models against the human data. (A) Rigid object condition. (B) Cup-and-ball condition. Interaction forces and positions and velocities of the cup and the ball from the same exemplary participant as in Figure 4 are shown. The shaded bands around the mean trajectories show 1 standard deviation. The two OFC responses (with and without impedance) are overlaid on the human data. (C) Modeling error quantified by the mean of the normalized root-mean-squared error of all trajectories. Each pair of dots connected by a line represents the modeling of one participant. The black line belongs to the shown participant.

Figures 5A and 5B illustrate the simulation results from the standard minimum-effort model with and without impedance, together with the experimental data for one participant. For the rigid-object condition (see Figure 5A), both models captured the qualitative patterns of the human movements in both perturbed and unperturbed trials. Specifically, both models produced the bell-shaped velocity profile and the velocity drop without rebound after the perturbation. However, the models differed in capturing the features of the interaction force around the perturbation. In the perturbed trials, the data showed an increase in the force level from before to after the perturbation (average across participants: 0.99±0.72N). While such a sudden increase was reproduced by the model that included impedance (0.38±0.33N), the model without impedance failed to account for this behavior and instead showed a decrease in force (−0.15±0.13N).

The inclusion of the ball dynamics separated the two models’ behavior further (see Figure 5B). Without impedance, the qualitative characteristics of the simulated movement deviated from the human data more distinctively. In contrast, with impedance, the OFC model modulated the interaction force and consequently the cup velocity less, leading to qualitatively similar behavior as seen in the human data. Similar to the rigid-object condition, the inclusion of impedance allowed the model to reproduce the discontinuous increase in interaction force from before to after the perturbation. Averaged across participants, the simulated interaction force increased by 0.81±0.54N (human data: 1.52±0.69N). This feature could not be captured in the absence of impedance, and the interaction force decreased by −0.15±0.08N in the simulation. Another salient behavioral feature in the perturbed cup-and-ball condition was the smooth rebound of the cup velocity after the perturbation. In the face of perturbations, both models closely replicated the drop and rebound of the cup velocity as well as the spike in interaction force.

The overall error between the simulated and measured behavior was quantified using an aggregate root-mean-square error of fit that took into account the trajectories of all cup-and-ball states and the interaction force (see Figure 5C). In both null and perturbed conditions, this overall modeling error was smaller when impedance was included in the model. A threeway repeated-measures ANOVA revealed statistically significant two-way and three-way interactions and main effects. The full ANOVA results are presented in Table S-1. Because the goal was to compare the modeling error with and without impedance in each test condition, planned pair-wise comparisons were made within each block using Tukey-Kramer tests. Results indicated statistically significant differences between the two models in all four condition blocks p=0.0016, $,p=0.0038,\;p=7.68\times 10^{-5}$ and p=0.013 for blocks 1 to 4 , respectively). The inclusion of impedance resulted in significantly better fit to the data in all conditions.

**Figure 6:**
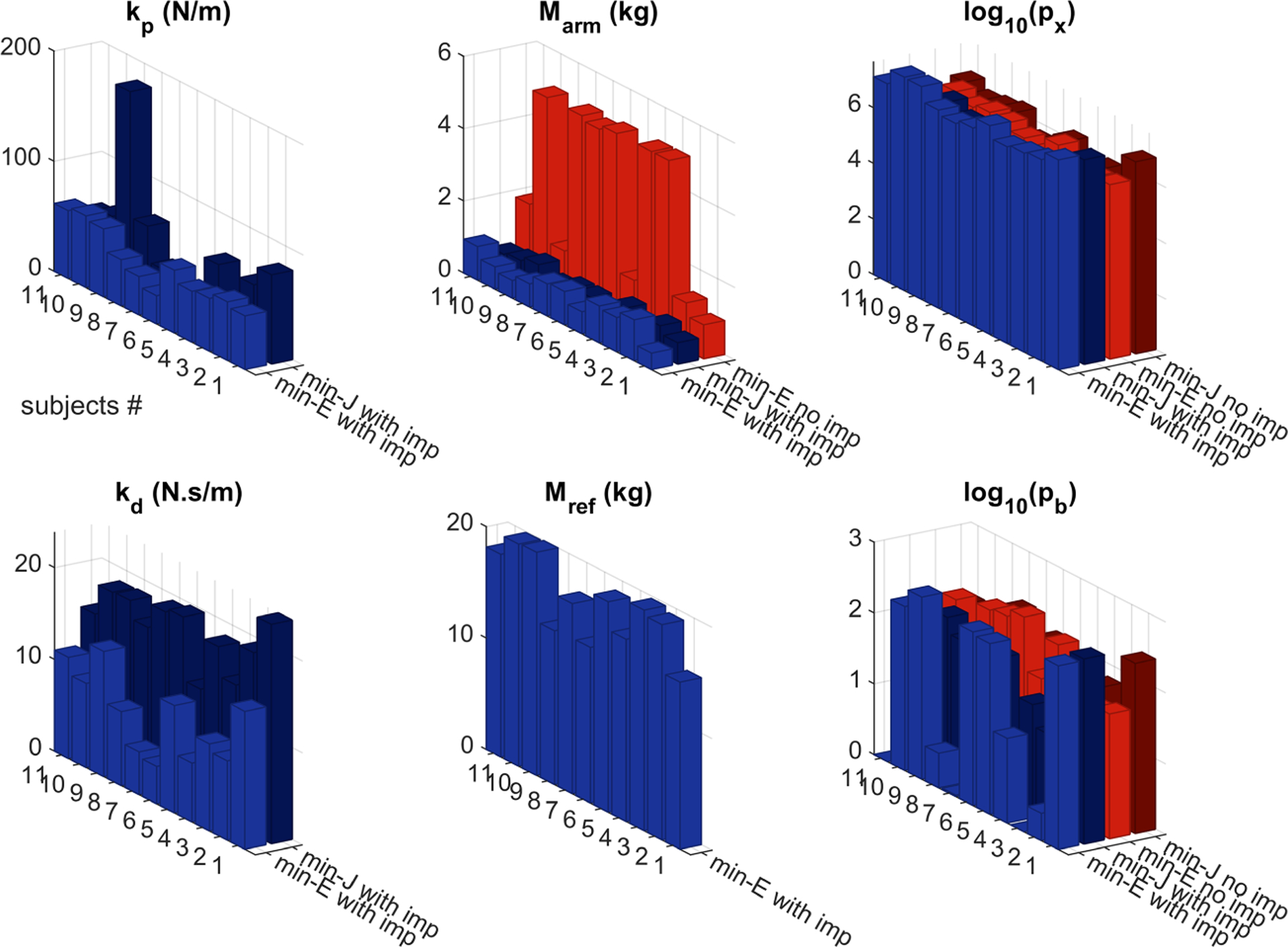
Model parameters fitted to each participant’s data. Impedance stiffness $k_{p}$ and damping $k_{d}$, arm mass $M_{arm}$, reference trajectory’s inertia $M_{ref}$, and penalties on cup and ball states, ${log}_{10}\;\left(p_{x}\right)$ and ${log}_{10}\;\left(p_{b}\right)$, are identified. See Table 4 for the average values.

The models involved a number of parameters that were fitted to individual participants’ data and are summarized in Figure 6 and Table 4. Results of the BIC analyses showed that the inclusion of impedance in the model reduced BIC for all participants with a difference of △BIC>61, suggesting that the model with impedance proved to be a better underlying modeling structure (see details in Figure S-1 in the supplementary material). These modeling results highlighted the importance of mechanical impedance for the overall system behavior.

#### 3.2.2 The Effects of the Objective Function.

For the above analyses, both models included minimization of effort as their objective function, consistent with many previous studies (Todorov & Jordan, 2002b; Diedrichsen et al., 2010). The following comparisons tested the influence of the objective function on the models’ behavior in the context of interactions with a complex object.

**Figure 7:**
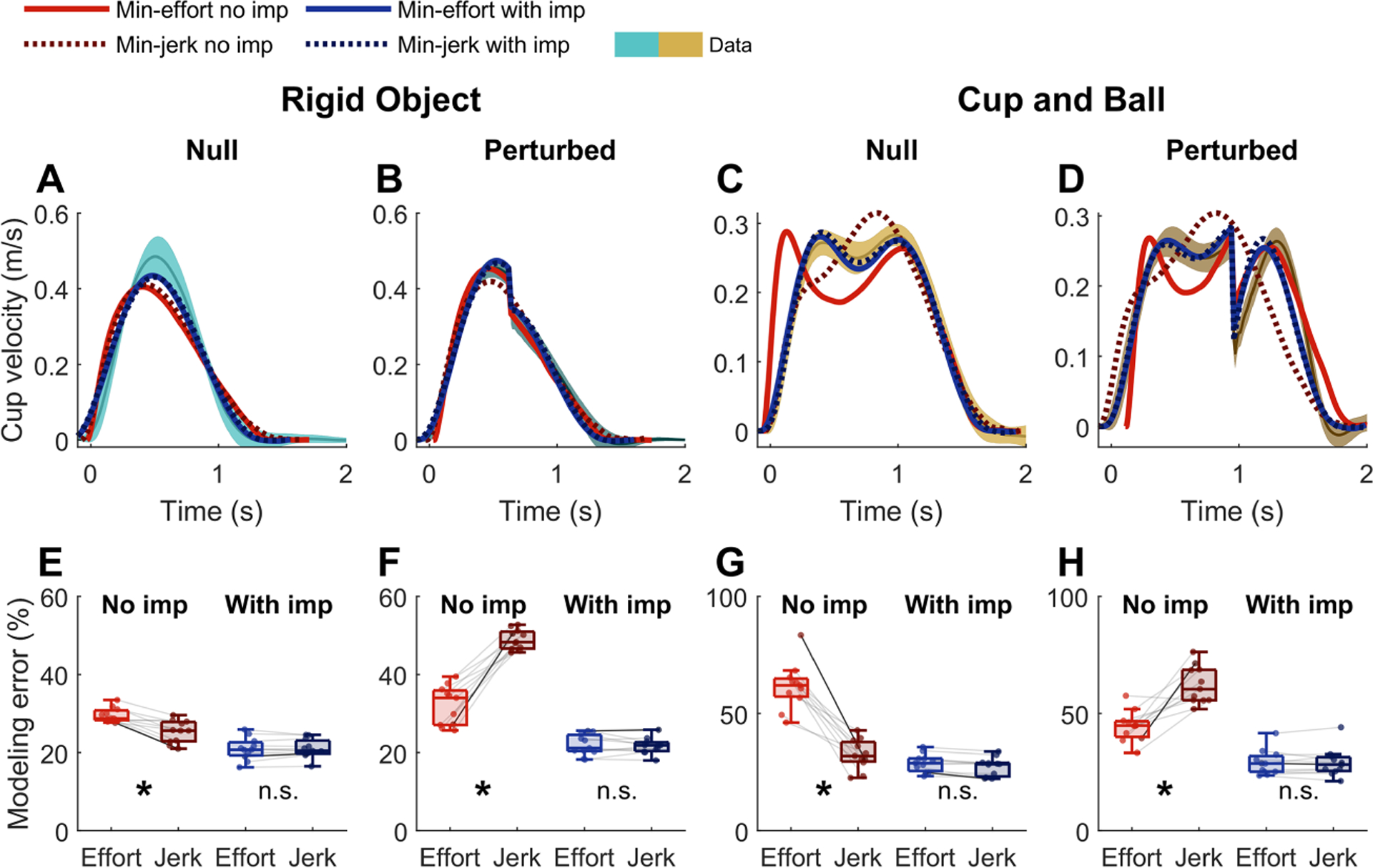
Effect of the objective function on model behavior. The minimumeffort and minimum-jerk criteria are compared in models with and without impedance. (A-D) The simulated cup trajectories overlaid on one example participant’s data (same participant as in Figure 4). (E-H) Quantitative comparisons of the overall modeling error across all participants. The asterisk indicates statistically significant differences (p<0.05) between the minimum-effort and minimum-jerk models; n.s. (not significant) indicates no statistically significant differences (p>0.05). Each thin line represents a participant; the solid black line belongs to the shown participant.

In interaction with the rigid object (see Figure 7A), all four model variants (with or without impedance, minimizing jerk, or minimizing effort) produced the expected smooth bell-shaped velocity profile. All of these models, with the exception of minimum-jerk without impedance, performed qualitatively similar to the human data when facing the perturbations in block 2 (see Figure 7B). Note that the minimum-jerk model directly prescribed the cup kinematics and, by design, the perturbation forces in block 2 did not affect the cup movement and did not produce the sudden velocity drop (dashed red line in Figure 7B).

In contrast, in the cup-and-ball condition, the two models without impedance produced distinct movement profiles (see Figure 7C). The minimum-jerk model variant generated a single-peaked velocity profile, as opposed to the pronounced double-peaked profile of the minimum-effort variant (compare the solid and dashed red lines in Figure 7C). However, when impedance was included, the two variants produced very similar movement patterns. Similar observations hold in the perturbed cup-and-ball condition (see Figure 7D): without impedance, the minimum-effort and minimum-jerk variants behaved distinctively from each other (solid and dashed red lines), while the two cost functions resulted in similar behavior when impedance was present in the model (solid and dashed blue lines).

These qualitative similarities and differences were clearly reflected in the quantitative error (see Figures 7E and 7F). Without impedance, the modeling errors from the minimum-effort and minimum-jerk variants were different in all four test conditions. However, with impedance, the two objective functions resulted in comparable modeling errors. A fourway repeated-measured ANOVA compared the modeling error with the factors model type (with versus without impedance), objective function (minimum-effort versus minimum-jerk), object type (rigid object versus cup-and-ball), and perturbation type (unperturbed versus perturbed). Results revealed that the model type produced a strong main effect $(F(10)=1069.1,\;\left.p=1.69\times 10^{-11}\right)$, while the cost function did not yield a significant main effect (F(10)=0.010,p=0.920) Only the three-way and four-way interactions that involved model type and cost function showed statistical significance (F(10)≥17.13,, p≤0.002). The full ANOVA results are presented in Table S-2. Planned pairwise comparisons within each test condition (see Figures 7E to 7H) showed that the errors from the two model variants without impedance were different in all four blocks (p≤0.0132). However, with impedance, the two model variants were not statistically distinguishable in any of the blocks (p≥0.195).

#### 3.2.3 The Effects of Sensory Feedback.

To investigate the role of sensory feedback on the models’ behavior, catch trials were analyzed. Note that catch trials were visually identical to the rest trials within the same block, and there was no indication that would inform the participants about the change in the perturbation condition. These trials afforded separating the effects of preplanning from a feedback-driven response in the face of the unexpected event.

**Figure 8:**
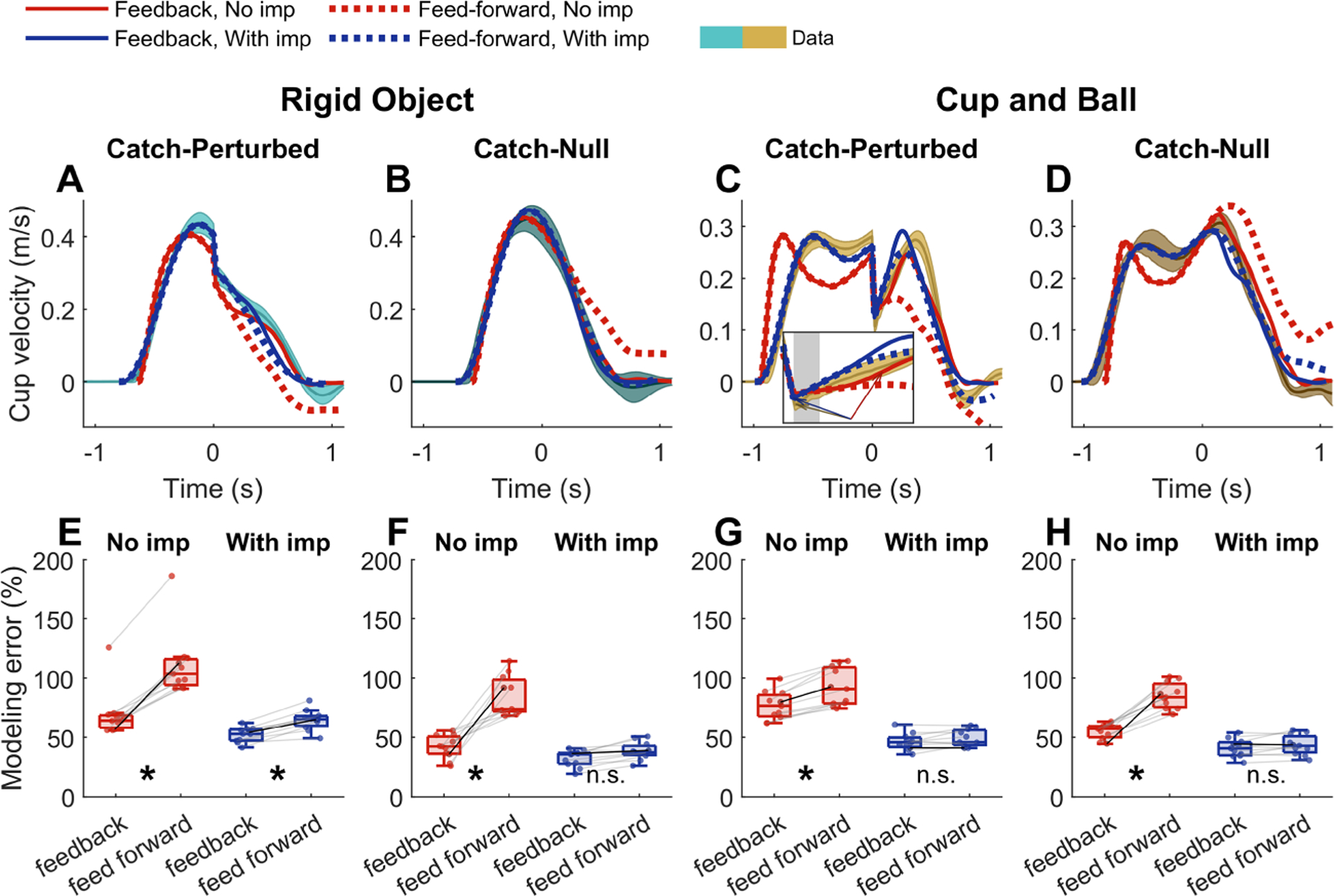
Catch trials to assess the effects of sensory feedback on model performance. In catch-perturbed trials, participants unexpectedly experienced a perturbation in a block where 95% were null trials. In catch-null trials, the visual cue (speed bump) alerted subjects to the upcoming perturbation as in most other trials, but no perturbation was applied. (A-D) Experimental and simulated results for catch trials: cyan and light brown bands represent one participant’s data for rigid-object and cup-and-ball conditions, respectively (same participant as in Figure 4). Solid lines represent the feedback model without (red) and with (blue) impedance; dashed lines represent the feedforward replay of the nominal control trajectories in the catch trial. The inset in panel C magnifies the trajectories around the perturbation. The shaded region corresponds to 50ms simulated sensory delay, and the arrows point to the moment of maximum cup acceleration. All time series of experimental data and simulations were aligned based on the moment when the cup arrived at the onset of perturbations, at 60% distance toward the target. (E−H) Overall error between the human data and simulation. The asterisk indicates statistically significant differences (p<0.05) between the feedback and feedforward models; n.s. indicates not significant (p>0.05). Each thin line represents one participant; the solid black line belongs to the participant shown above.

Figures 8A to 8D illustrate the experimental and simulation results for both types of catch trials. Only the standard minimum-effort models were analyzed here. When sensory feedback was present, the model without impedance (solid red lines) could drive the rigid object to the target and stop there, despite the unexpected perturbation in the catch-perturbed trial (see Figure 8A). When sensory feedback was removed from the model (dashed red lines), the simulated cup velocity no longer reached zero velocity at the end of the trial; the cup stopped and moved back in the opposite direction with negative velocity by the end of the trial (see Figure 8A). Interestingly, by introducing impedance, the feedback and feedforward control policies became similar (respectively, solid and dashed blue lines in Figure 8A). Both models could recover from the unexpected perturbation and successfully transport the rigid object to the target and stop there. Similar behavior was observed in the catch-null trials (see Figure 8B), where the perturbation was unexpectedly removed: without impedance, the feedforward model could not cope with the unexpected scenario. In contrast, the inclusion of impedance enabled both models, with or without sensory feedback, to successfully finish the task. The inclusion of the ball enhanced these effects, and the undershoot and overshoot of the cup velocity with the feedforward were even more pronounced in the absence of impedance (see Figures 8C and 8D). Both variants with impedance, however, were successful at the task.

The rebound in the cup velocity following the perturbation (see Figure 8C) distinguished the models even further. Without impedance, the velocity rebound deviated from the data even when the control model utilized sensory feedback (highlighted in the inset of Figure 8C). In the data, the cup acceleration peaked immediately (within 5ms) after the perturbation ended. Across participants, the average cup acceleration before perturbation onset was $0.15\pm 0.09\;m/s^{2}$ and rose to $1.06\pm 0.27\;m/s^{2}$ immediately (within 5ms) after the perturbation. This instantaneous behavior was observed in all participants. However, in the absence of impedance, the simulated cup acceleration did not exhibit this instantaneous rise; cup acceleration was $0.098\pm 0.091\;m/s^{2}$ and $0.072\pm 0.075\;m/s^{2}$, before and after perturbation, respectively. In this case, the controller received sensory feedback with a 50ms delay and could respond to the perturbation only after processing the delayed information. Consequently, the cup acceleration took 152±8ms to rise to a value that was comparable to the measured peak acceleration, within 1 standard deviation from the mean of the data. The model with impedance reproduced this instantaneous response after the perturbation, and cup acceleration peaked immediately following the perturbation and reached $0.71\pm 0.30\;m/s^{2}$ (from $0.17\pm 0.06\;m/s^{2}$ at perturbation onset). Note that although the feedforward model with impedance had an extra inertia parameter $M_{\text{ref}}$ compared to the model with no impedance, this additional inertia cannot account for the rebound of cup velocity and acceleration after the perturbation (see Figure 8C). Such immediate rebound is achieved through a mechanism that can store and release energy in the face of a perturbation, which in our case is the stiffness element in the model.

To quantify and compare the error between different models, only the trajectories after the perturbation were taken into account because the feedback and feedforward responses were identical before the perturbation. As Figures 8E to 8H show, without impedance, the feedforward variant consistently underperformed the feedback-driven one in all four conditions. However, the errors from the feedback and feedforward variants of the models were comparable in the presence of impedance. A four-way repeated-measures ANOVA was used to analyze the modeling error; the four factors were model type (with versus without impedance), feedback structure (feedback versus feedforward), object type (rigid object versus cup-and-ball), and perturbation type (catch-null versus catchperturbed). All two-, three- and four-way interactions that involved both model type and feedback structure revealed statistical significance (F(10)≥6.97,p≤0.0247). Further, both model type and feedback structure showed strong main effects $\left(F(10)\geq 99.1,p\leq 1.66\times 10^{-6}\right)$. The ANOVA results are shown in the supplementary materials. Following these results, planned pairwise comparisons of the model variants within each block (see Figures 8E to 8H) showed that the feedforward model without impedance underperformed the feedback one in all test conditions with statistical significance $\left(p\leq 5.61\times 10^{-3}\right)$. However, the two variants of the model with impedance were not statistically different in blocks 2,3 and 4(p≥0.084); only in block 1 did the feedback model perform better than the feedforward variant $\left(p=5.80\times 10^{-4}\right)$.

## 4 Discussion

To date, numerous studies have examined the roles of the body mechanics, optimality principles, and sensory feedback for the control of voluntary goal-directed movements. Typically the experiments were carefully designed to isolate and explore the concept in question—for example, the cost functions (Todorov, 2004; Wolpert et al., 1995), mechanical impedance (Burdet et al., 2001; Selen et al., 2009), or the interaction between feedback and feedforward pathways (Maeda et al., 2020). The interplay of these critical elements, however, has received less attention, with only few exceptions. For instance, the interplay between sensory feedback and impedance was studied in an isometric holding task (Crevecoeur & Scott, 2014) and in a postural control task (Van Wouwe et al., 2022). Common to these studies are the simplified experimental tasks to generate clear data; for example, the feedforward command could safely be assumed constant in the cited articles. However, such simplified tasks may not be rich enough to reveal the full relation between the constituent elements of motor control. Hence, the goal of this work was to study the interplay of body mechanics, optimality criteria, and sensory feedback in an experimental assay in which humans interacted with an object that introduced internal dynamics with an underactuated degree of freedom. Such a movement task posed a new set of control challenges that afforded a more intricate view into control processes.

### 4.1 Contributions of Impedance.

The body and its mechanics (embodiment) play a significant role in determining control, especially in more complex movements in interaction with objects and the environment. Thus, a model’s prediction about the brain’s role in motor control critically depends on the neuromechanical details included in the control model (Pinter et al., 2012). To address the crucial interplay of top-down control and body dynamics, this study examined OFC’s predicted behavior when the model for the body was extended with a simple representation of its inherent dynamics, mechanical impedance. To evaluate the contribution of impedance, different variants of OFC were developed and compared.

Comparison of human kinematics in object interactions with the corresponding model simulations showed that the OFC model without impedance failed to reproduce several salient features in human performance. Several results highlighted the superior performance of the model that included impedance. First, the model with impedance produced kinematic profiles that deviated significantly less from the experimental trajectories, as quantified by the aggregate RMS error (see Figure 5C). Second, in the absence of impedance, the simulations could not reproduce the sudden increase in the interaction force immediately following the perturbation, a robust feature in the human data. Third, without impedance, the model’s response showed a noticeable delay to an unexpected perturbation due to the 50 ms sensory delay in the model (see Figure 8C). No such delay was observed in the human data. However, when an impedance element was included, the model captured the subjects’ overall movement profiles to a significantly greater degree. The impedance element acted as an energy buffer, allowing the model to capture the discontinuity in the interaction forces following a perturbation. Impedance also allowed the model to replicate the instantaneous response to unexpected perturbations.

The two phenomena at the perturbation, the change in interaction force and acceleration post-perturbation, occurred at a very fast timescale (<20ms). Neither voluntary nor involuntary neural responses could be generated at such timescales. For comparison, the fastest stretch reflex in upper extremities starts only 20 to 50ms after perturbation onset (Pruszynski & Scott, 2012). These observations imply that the instantaneous responses should be attributed to the biomechanics of the arm. Within the 20ms duration of the impulsive perturbation force, the cup velocity decreased significantly, while the cup position and also the position and velocity of the impedance’s zero-force trajectory changed only negligibly. The drop in the cup’s velocity caused a sudden increase in the damping force and, ultimately, an increase in the interaction force. The same instantaneous rise in interaction force was also the reason for the immediate rebound of cup velocity after the unexpected perturbation. The model without impedance lacked this mechanical contribution, and the velocity could accelerate and rebound only after processing the delayed feedback to command an increase in muscle force.

The impedance included in the model consisted of a pair of linear and constant spring and damper elements. Despite this simple design, the model clearly outperformed the one without impedance. In reality, nonlinear muscle, joint, and inter-limb mechanics contribute to the effective impedance of the limb, resulting in stiffness values that vary with posture (Gomi & Kawato, 1997; Perreault et al., 2001) or the evolving movement (Gomi & Kawato, 1996; Lee et al., 2012). Accurate representation of mechanical impedance is crucial to peel back the mechanics of the body and reveal the descending commands (Latash, 1994; Domen et al., 1999; Hogan, 2017; Hermus et al., 2020). In addition to the passive contributions of the musculoskeletal properties, task-dependent modulation of stiffness has also been proposed as a means of control (Burdet et al., 2001, 2006; Franklin et al., 2007). The effects of time-varying modulation of impedance have been studied in musculoskeletal simulations (Van Wouwe et al., 2022; Xiong et al., 2021; Berret & Jean, 2020; Shourijeh & Fregly, 2020) and in robot control (Hammoud et al., 2021). Results suggested that the brain may indeed use impedance-modulation as another control mechanism. However, it remains a challenge to parse the contributions of active stiffness modulation from the variations caused by the passive mechanics of the body due to the persistent challenges of estimating time-varying impedance in vivo (Rouse et al., 2014). To avoid unnecessary complications and assumptions, the simple time-invariant model was adopted that still describes the human data remarkably well.

The behavior of the models critically depended on the parameter values used in the simulations. Although the models were highly simplified compared to the human physiology, the lumped parameter values can be discussed in relation to biomechanical properties of the body. The identified impedance parameters were within the range of previously reported values for the end-point (hand) impedance, although the range reported in the literature spans one order of magnitude. The estimated end-point stiffness in the literature ranged from 40N/m (Nayeem et al., 2020; Nayeem et al., 2023) to 200N/m (Maurice et al., 2018) during rhythmic movements, and > 250 N/m (Perreault et al., 2001; Krutky et al., 2013) and >60N/m (Mussa-Ivaldi et al., 1985) in isometric holding tasks where short-range stiffness was a major contributor. In comparison, the end-point stiffness identified via matching the model to the experimental data in our work was 49±12N/m and 59±47N/m in the minimum-effort and minimum-jerk models, respectively. Likewise, our estimated damping was 9.3±3.4N.s / m and 18±3N.s/m in the two models, which were within the range of 10−50N.s/m reported in (Nayeem et al., 2020; Nayeem et al., 2023; Maurice et al., 2018; Krutky et al., 2013).

In the models that included impedance, the mass of the arm $M_{\text{arm}}$ represented the effective inertia after the impedance element; it was estimated to be 0.78±0.23kg (minimum-effort) and 0.61±0.14kg (minimum-jerk), which is slightly higher than the mass of the hand—on average 0.5% to 0.6% of body mass (Dumas et al., 2007), approximately 0.3 to 0.55 kg. This consistency can be explained by noting that forearm and upper arm partly contributed to the effective post-impedance inertia. In the absence of impedance, the identified $M_{\text{arm}}$ was 3.3±2.0kg, which was close to the total mass of the arm—4.2% to 4.7% of body mass or 2.5 to 4.3 kg (Dumas et al., 2007). The lumped parameter $M_{\text{ref}}=17\pm 2\;kg$ obtained when fitting the data was much larger than that of the arm. This could be physiologically realistic because this parameter represented the effective inertia of all forceproducing machinery in the body. This effective inertia might include the contributions of trunk and even leg muscles, as it is not unreasonable to assume that core muscles also engage in responding to perturbations, hence, leading to a relatively larger inertia value for $M_{\text{ref}}$.

Not only did the added mechanical impedance replicate human movements much better; it also provided further important insights about two cornerstones of optimal feedback control: detailed features of the cost function became less important when impedance was added, and sensory feedback for the processing of error and correction became less critical. These two issues are discussed next.

### 4.2 Contributions of the Cost Function.

To answer why a certain movement pattern is chosen over possible alternatives, the cornerstone of optimal control theory is that the controller, or the brain, seeks to minimize a certain objective function. In movement neuroscience, there has been considerable debate as to which objective function the brain is trying to optimize (see Todorov, 2004, for a review). A multitude of objective functions have been proposed, ranging from the kinematics-based objectives, for example, minimum jerk (Flash & Hogan, 1985; Svinin et al., 2019), minimum crackle (Dingwell et al., 2004), and minimum acceleration (Leib & Karniel, 2012), to the kinetics-based ones such as minimum torque change (Uno et al., 1989) and minimum muscle effort (Todorov & Jordan, 2002b; Ronsse et al., 2010), to the minimum variance cost (Harris & Wolpert, 1998) and the physiological energy expenditure (Anderson & Pandy, 2001; Wong et al., 2021). All of these objectives were able to describe human movements reasonably well in their respective experimental contexts. Support for any of these objectives typically involved comparing them against other objectives for a given task and then choosing the one that better matched the data. In the present study, the comparisons made between the two OFC variants with two different objective functions, minimum effort and jerk, also displayed distinct differences in the movement patterns. Strikingly, though, when impedance was included in the controller, no quantifiable differences were observed, and both cost functions replicated the human behavior equally well. This finding suggests that when OFC included even minimal biomechanical features, details of the objective function may become inconsequential. In the same spirit, Diedrichsen and colleagues (2010) have shown that under the assumption of signal-dependent noise, the objective of reducing effort is equivalent to reducing end-point variance. Similarly, Wong et al. (2021) showed that smooth movements are more energy efficient. Here, it is shown that the physiologically realistic element of arm impedance masks distinctions between effort and smoothness. One important implication is that the underlying control principles of the brain may be fundamentally unobservable due to the filtering effects of the body mechanics. However, it needs to be kept in mind that this finding was obtained in the context of interactions with a complex object. To what degree this blurring of details holds for other tasks requires more investigation.

### 4.3 Contributions of Sensory Feedback.

Features of feedforward (preplanned) and feedback (online corrections) control mechanisms are intertwined in coordinated human movement. Following Woodworth in 1899, numerous authors have distinguished a ballistic (feedforward) and a homing-in phase associated with error corrections (feedback) in simple pointing movements (Woodworth, 1899; Meyer et al., 1988). In more recent years, Crevecoeur & Scott (2014) argued that feedback control was necessary to complement a feedforward command to replicate the human response to perturbations in an isometric task. Yeo et al. (2016) showed that when sensory information was uncertain, human movements could not be explained by feedback control alone and feedforward pathways were necessary. Even in tasks that heavily rely on continuous sensory information, such as balancing a stick on the fingertip, there are compelling arguments for intermittent episodes of feedforward predictive control (Loram et al., 2012).

This study used a dynamically complex task where the underactuated object created perturbations depending on the hand’s actions. In addition to expected perturbations, unexpected perturbations in the catch trials were given to identify the role of sensory feedback. The simulations of the catch trials without an impedance element showed that the feedforward replay of the OFC’s control command was unable to cope with the perturbations or their unexpected absence. This behavior did not match the subjects’ behavior (see Figure 8). On the other hand, the feedback-driven OFC model achieved a better fit to the data with significantly smaller model error. Based on these results, one may readily conclude that a feedback loop is necessary. However, when the model included impedance, both the feedback and feedforward variants replicated the experimental data equally well. These findings showed that mechanical effects may have intricate contributions to coping with complex events. Even a task that involves underactuated dynamics and challenging requirements, such as responding to the disturbance without “spilling the coffee” may be achieved by a feedforward control scheme as long as it is mediated by body mechanics.

It must be noted that our results do not simply provide support for either feedforward or feedback control. Instead, the key insight from these results is that under biomechanically more plausible assumptions, the distinction between predictive feedforward and sensory-driven feedback control may become less apparent. Similar effects were reported previously in a simple reaching task by Berret and Jean (2020), who showed that attenuation of task-dependent errors could be achieved by a feedforward tuning of the arm impedance. It should also be noted that these results must be interpreted in the context of the chosen experimental task. It is obvious that large enough and long enough perturbations can disrupt the movement to a degree that requires sensory-driven corrections in terms of both long-latency reflexes and voluntary responses. Indeed, Crevecoeur and Scott (2014) showed that optimal feedback corrections were necessary to complement mechanical impedance after large perturbations in a holding task. However, given that arm impedance is nonlinear (for example, it exhibits short-range stiffness; Joyce et al., 1969; Rack & Westbury, 1974) and that it changes with postural configuration (Pando et al., 2014) and movement (Gomi & Kawato, 1996; Lee et al., 2012), great care is needed when searching for the control structure using perturbations. New computational techniques that simultaneously model feedback and feedforward pathways as developed in a recent study may provide new insights (Van Wouwe et al., 2022).

### 4.4 Role of Impedance: Two Perspectives.

From a mathematical perspective, the impedance element in the model introduced three additional states to the dynamics of the system that the optimal feedback controller had to address. However, unlike inertial mechanics that can also increase the number of states (e.g., in multi-segment arm models), impedance endows the system with potential energy storage and dissipation properties. These properties could not be achieved with inertial mechanics alone. The implications of this physical and mathematical change in the system can be interpreted from two perspectives.

#### 4.4.1 Brain Controls the Body.

A first position puts priority to the fact that the brain is the site of control and the body is the system to be controlled. Thus, the brain as the sole control authority is fully responsible for defining the characteristics of the movements based on knowledge about the body. Grounded in this dualistic viewpoint, much of neuroscience has tried to shed light on the neural representations and neural activity patterns for the generation of movements. The search for the “control signals” in the primary motor or parietal cortices that directly correlate with the behavior at the single-neuron (Georgopoulos et al., 1986) or population level (Churchland et al., 2012; Shenoy et al., 2013) follows this basic position, if not philosophically, then at least practically. To facilitate this search, the movements themselves have been held simple to allow many repetitions under the “same” conditions and also to eliminate any interactive effects from the environment. Many studies on intracortical data in nonhuman primates have adopted such simple center-out reaching (Georgopoulos et al., 1986; Churchland et al., 2012) or cranking tasks (Russo et al., 2020). While intriguing insights have been gained, interactions with an object and the environment would present a step up in complexity and in conceptual terms. Our focus on the interaction with a dynamical object indicates that the physical properties of the body may present important challenges to the interpretability of purely top-down perspectives. Concretely, following our results, issues whether incoming sensory information affected the behavior and whether the cost function was effort or smoothness became blurred by adding simple biomechanical elements. In light of these findings it becomes evident that specific facets of control might be filtered out by the dynamics of the body.

#### 4.4.2 Brain and Body Work Together.

In a second view, innate dynamical properties of the body, such as mechanical impedance, are interpreted as a lower-level autonomous control authority that shares the control responsibility with the higher-level information processors. Thus, this embodiment of control means that not all characteristics of observable behavior can be attributed to the higher-level controller. The significance of the innate passive dynamics for producing movements was strikingly demonstrated in a series of passive walkers (McGeer, 1990; Collins et al., 2005). These non-actuated mechanical linkages could produce stable gaits in the absence of any motors, sensors, or a “brain.” Passive interactions of the body with a complex environment could even allow a dead fish to swim upstream (Beal et al., 2006).

Undeniably, functional human behavior is richer than these passive movements, and bodily mechanics alone cannot generate the full spectrum of functionally specific behavior. Hence, descending neural commands need to be sensitively interfaced to not only account for, but also leverage bodily mechanics. The physical properties of the body provide useful resources for embodied computation for both perception and action. Morphological computation for guided actions is well studied in animals. For instance, a housefly’s compound eyes can afford complex navigation skills using simple visuomotor circuitry (Franceschini et al., 1992), and the biomechanics of a goat’s hoof enables localized slip control in rough terrains (Abad et al., 2016). Similarly in humans, it has been demonstrated that joint compliance plays a significant role in accurate haptic perception: correct tuning of stiffness largely simplifies the computational load during control of movement (Sornkarn et al., 2016). In our results, mechanical impedance of the arm proved capable of negotiating the unexpected perturbations in a feedback-deprived control scheme, highlighting the possibility of producing “intelligent” responses through morphological or embodied computation.

Further, physical processes on multiple scales, ranging from passive muscle force enhancement via titin proteins (Herzog, 2018) to limb inertial mechanics, provide means to distribute control authority across the body, thereby alleviating some of the computational burden on the brain. In human movement neuroscience, only a few approaches have placed emphasis on the dynamics emerging from the interplay between control and the dynamics of the body. From a neurophysiological perspective, the equilibrium point hypothesis aimed to reveal how the central nervous system specifies lambda, the equilibrium or reference configuration of a limb, via tuning of the phasic and tonic stretch reflexes (Feldman, 1986). From a mechanical perspective, a number of studies have demonstrated the tuning of impedance during movement (Burdet et al., 2001; Franklin et al., 2007). Further, studies on bimanual coordination have detailed how rhythmically moving limbs self-organize into autonomous nonlinear oscillators that synchronize into stable phase relations without requiring detailed top-down control (Sternad et al., 1998; Ronsse et al., 2009). Hence, movements may be the emerging response of the body leveraging the environment with simple parameter specifications or motor commands (Sternad et al., 2000). However, to date, these approaches have been limited to simple tasks. In order to advance one step further, this study employed optimal feedback control as a high-level controller that guides the behavior of the low-level dynamics as a way to reconcile the duality between information processing in the brain and embodiment. Such interfacing ultimately shapes the final behavior.

## 5. Conclusion

This study highlighted the importance of mechanical impedance of the body in modeling human movements. The experimental assay was humans transporting a complex object with underactuated dynamics. Even the simple linear transport revealed that the unactuated degree of freedom required more than a bell-shaped velocity profile of the hand, quintessential in reaching or transporting a rigid body. Without a regard for the compliance of the body, an information processing OFC model fell short of reproducing characteristic features of human movements; a successful optimal feedback controller necessitated embodiment (mechanical impedance) in the control loop. Perturbation trials further revealed that impedance buffered the energy and thereby obviated the need for feedback in the optimal controller. Including impedance into the model also blurred the distinction between cost functions previously suggested for various reaching tasks. Taken together, our results emphasize that attention must be given to body mechanics to better understand the controller. The results highlight the formidable challenge to gain insights into the neural controller due to its tight intertwining with the body’s and the task’s dynamics.

## Supplementary Material

Sharif.Razavian.Supplementary.Material

## Figures and Tables

**Table 1:** Model Parameters That Were Kept the Same in All Models.

Parameter	Description	Value*
*d*	Sensory delay	0.05
***ε***	Standard deviation of control-dependent process noise	1
***ϵ***	Standard deviation of state-dependent sensory noise	0
***ξ***	Standard deviation of additive process noise^†^	1 × 10^−4^
***ω***	Covariance matrix of additive sensory noise	Diag(1 × 10^−5^)
***η***	Covariance matrix of internal noise in state estimator (Todorov, 2005)	Diag(1 × 10^−8^)
*n*	Number of time steps to hold the object at target	50
*τ*	Time constant of the first-order muscle dynamics	0.03

* In corresponding SI units and appropriate dimensions.

† Only for control-affected state (e.g., muscle force) and the perturbation state.

**Table 2:** Free Parameters for Model Fitting.

		Min-effort		Min-jerk	
Parameter	Bounds (lower, upper)	No impedance	With impedance	No impedance	With impedance
*p*_*x*_	(10^2^, 10^9^)	✓	✓	✓	✓
*p*_*b*_	(1, 10^5^)	✓	✓	✓	✓
*M*_*arm*_ (kg)	(0, 5)	✓	✓	N/A	✓
*k*_*p*_ (N/m)	(0.1, 200)	N/A	✓	N/A	✓
*k*_*d*_ (N.s/m)	(0.1, 200)	N/A	✓	N/A	✓
*M*_*ref*_ (kg)	(0.05, 20)	N/A	✓	N/A	N/A

Note: The check marks indicate that a given parameter was present in a given model.

**Table 3:** Trial Durations in the 4 Experimental Blocks.

Block	All trials	First 25 trials	Last 25 trials	Wilcoxon signed rank test
1	1.43 ± 0.24 s	1.45 ± 0.44 s	1.41 ± 0.25 s	*W* = 40, *p* = 0.57
2	1.43 ± 0.20 s	1.45 ± 0.23 s	1.40 ± 0.24 s	*W* = 50, *p* = 0.15
3	1.91 ± 0.34 s	1.97 ± 0.37 s	1.86 ± 0.25 s	*W* = 95, *p* = 0.019
4	1.81 ± 0.27 s	1.83 ± 0.23 s	1.80 ± 0.22 s	*W* = 43, *p* = 0.41

Note: Median trial durations across all participants are reported.

**Table 4:** Mean ± Standard Deviation of the Identified Parameters, Averaged across Participants.

	Min-effort		Min-jerk	
Parameter	No impedance	With impedance	No impedance	With impedance
*k*_*p*_ (N/m)	N/A	49.47 ± 12.08	N/A	58.92 ± 47.49
*k*_*d*_ (N.s/m)	N/A	9.26 ± 3.45	N/A	18.04 ± 2.96
*M*_*arm*_ (kg)	3.28 ± 1.96	0.79 ± 0.23	N/A	0.61 ± 0.14
*M*_*ref*_ (kg)	N/A	17.46 ± 2.39	N/A	N/A
log_10_ (*p*_*x*_)	6.45 ± 0.22	7.28 ± 0.26	6.17 ± 0.41	6.34 ± 0.50
log_10_ (*p*_*b*_)	2.08 ± 0.31	1.31 ± 1.16	1.91 ± 0.30	1.44 ± 0.80

Note: Impedance stiffness $k_{p}$ and damping $k_{d}$, arm mass $M_{arm}$, reference trajectory’s inertia $M_{ref}$, and penalties on cup and ball states ${log}_{10}\;\left(p_{b}\right),{log}_{10}\;\left(p_{x}\right)$ are reported. N/A indicates that the parameter was not included in the model.
