# Supplementary material for: Body Mechanics, Optimality, and Sensory Feedback in the Human Control of Complex Objects
Source: Neural Comput. Author manuscript; Available in PMC 2023 Jun 7. (PMC10246336; doi:10.1162/neco_a_01576)
Supplement: Sharif.Razavian.Supplementary.Material [file NIHMS1904888-supplement-Sharif_Razavian_Supplementary_Material.pdf]

Supplementary materials

Body mechanics, optimality, and sensory feedback in the human control of complex objects

Reza Sharif Razavian[1,2†*], Mohsen Sadeghi[1,2†], Salah Bazzi[2,3†], Rashida Nayeem[2], Dagmar Sternad[1,2,3,4]

1 Department of Biology, Northeastern University, Boston, MA, USA

2 Department of Electrical and Computer Engineering, Northeastern University, Boston, MA, USA

3 Institute for Experiential Robotics, Northeastern University, Boston, MA, USA

4 Department of Physics, Northeastern University, Boston, MA, USA

† These authors contributed equally

* razavian.reza@nau.edu

Bayesian Information Criterion

The average absolute error for all four models fitted to each participant data and the corresponding and the Bayesian Information Criterion (BIC) are provided in **Fig. S-1**. Refer to the main text for the details of calculations.

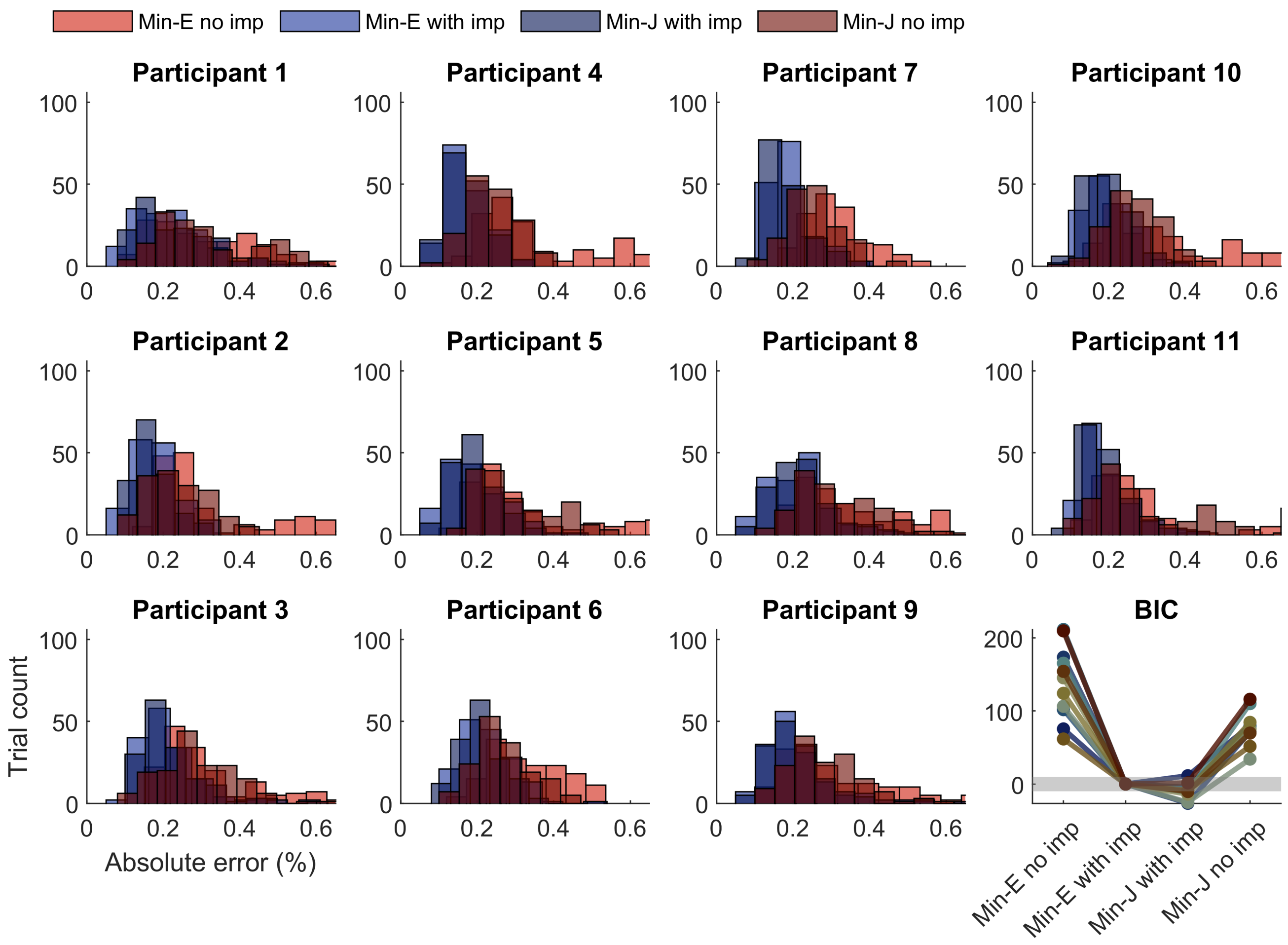

Fig S-1. The average absolute error for all four models fitted to each participant data. The Bayesian Information Criterion (BIC) is calculated from the absolute error.

Individual Participants' Data

All eleven participants' data and their fitted models are shown in **Fig. S-2** to **Fig. S-45**. For description of the figures, please refer to the main text. Participant #11's data were shown in the main text.

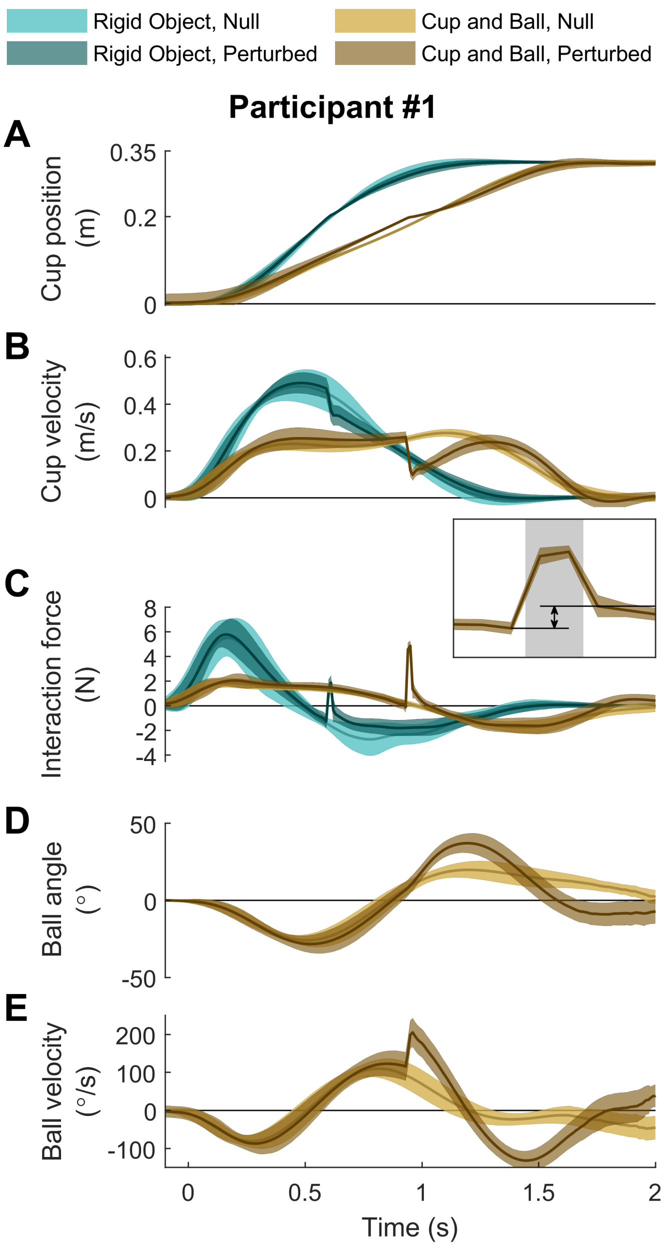

Fig S-2. Participant #1 behavior in the four blocks.

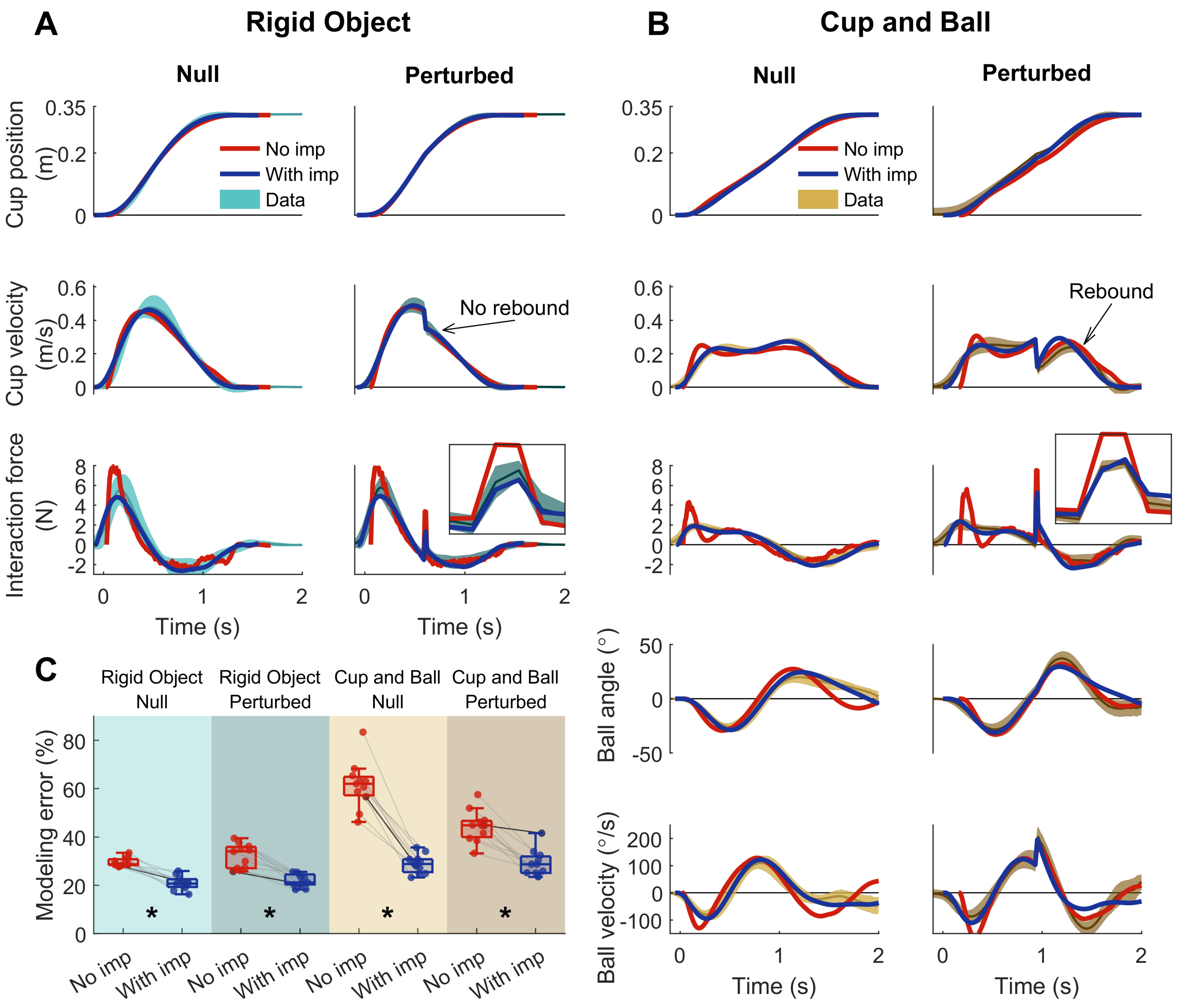

Fig S-3. Comparison of the model responses with participant #1's data.

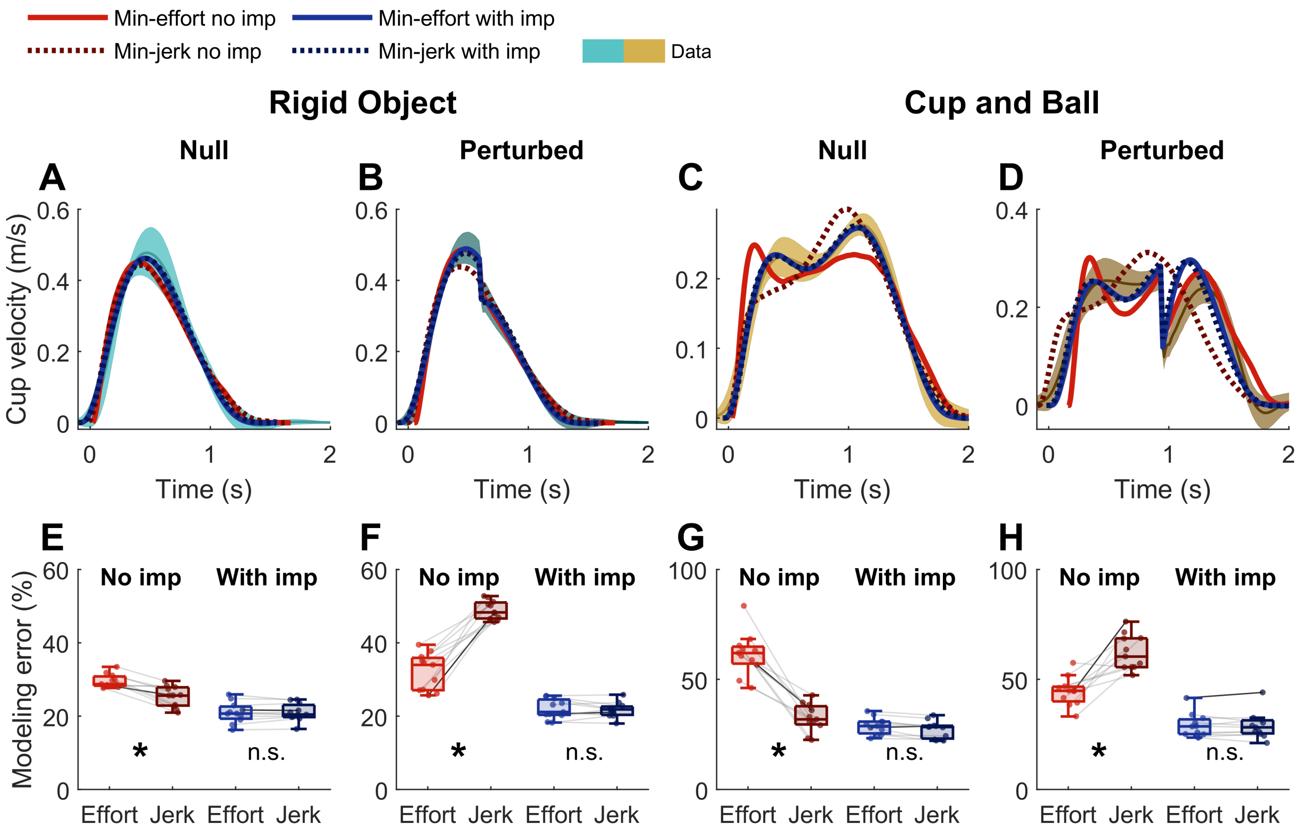

Fig S-4. Effects of the optimality criterion on the models fitted to participant #1's data

-2.0in0in

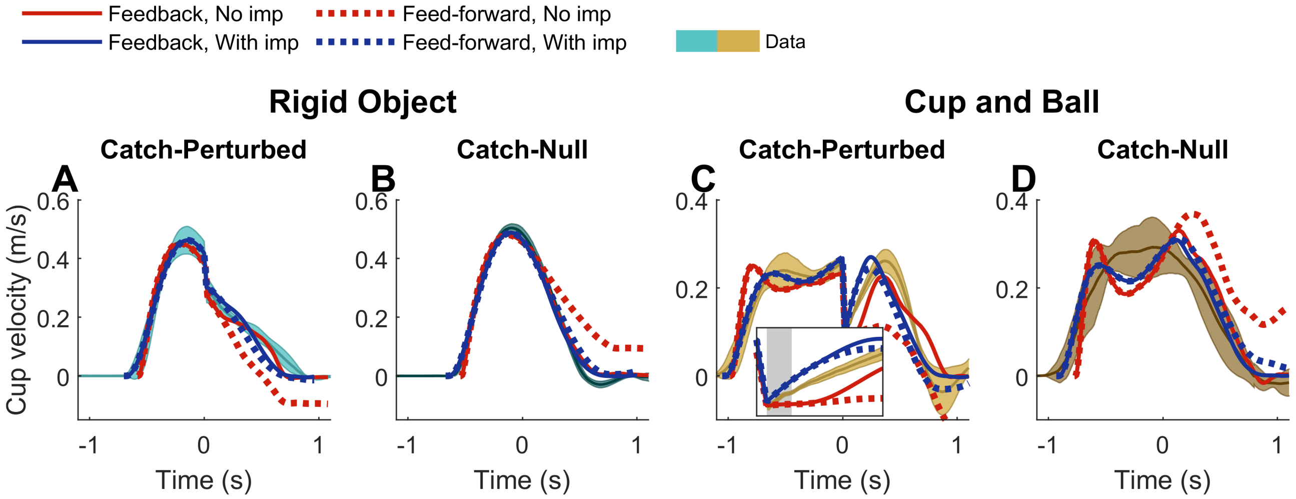

Fig S-5. Participant #1's catch trials and the the simulated behaviors.

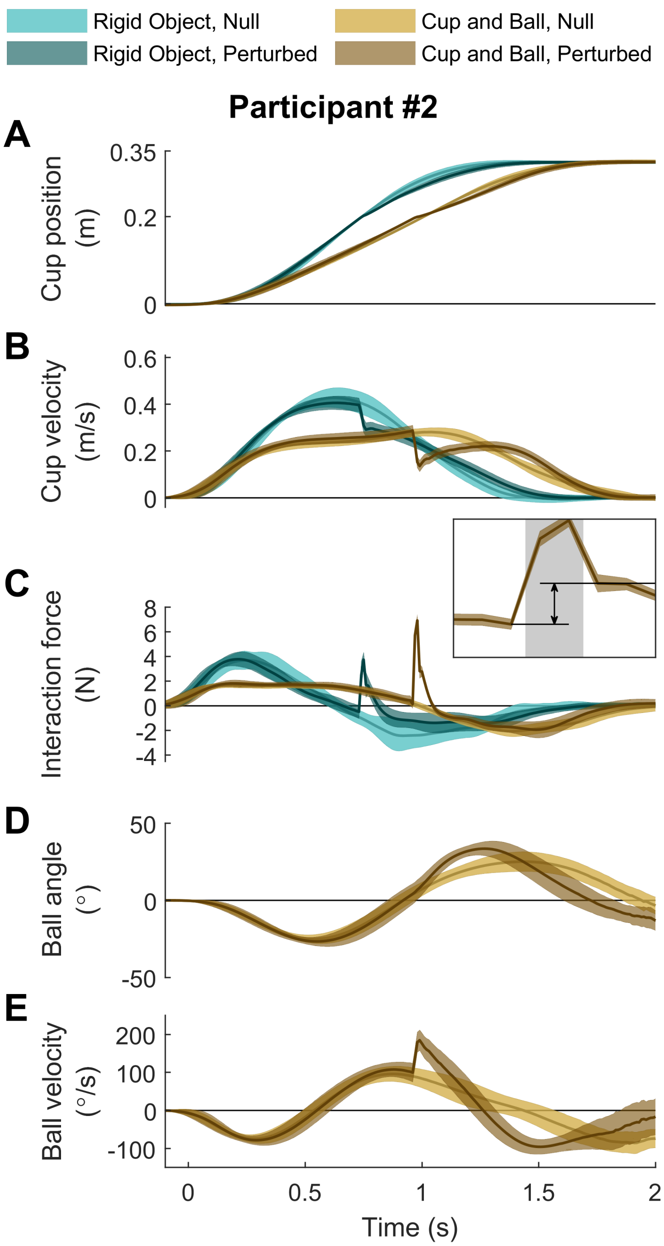

Fig S-6. Participant #2 behavior in the four blocks.

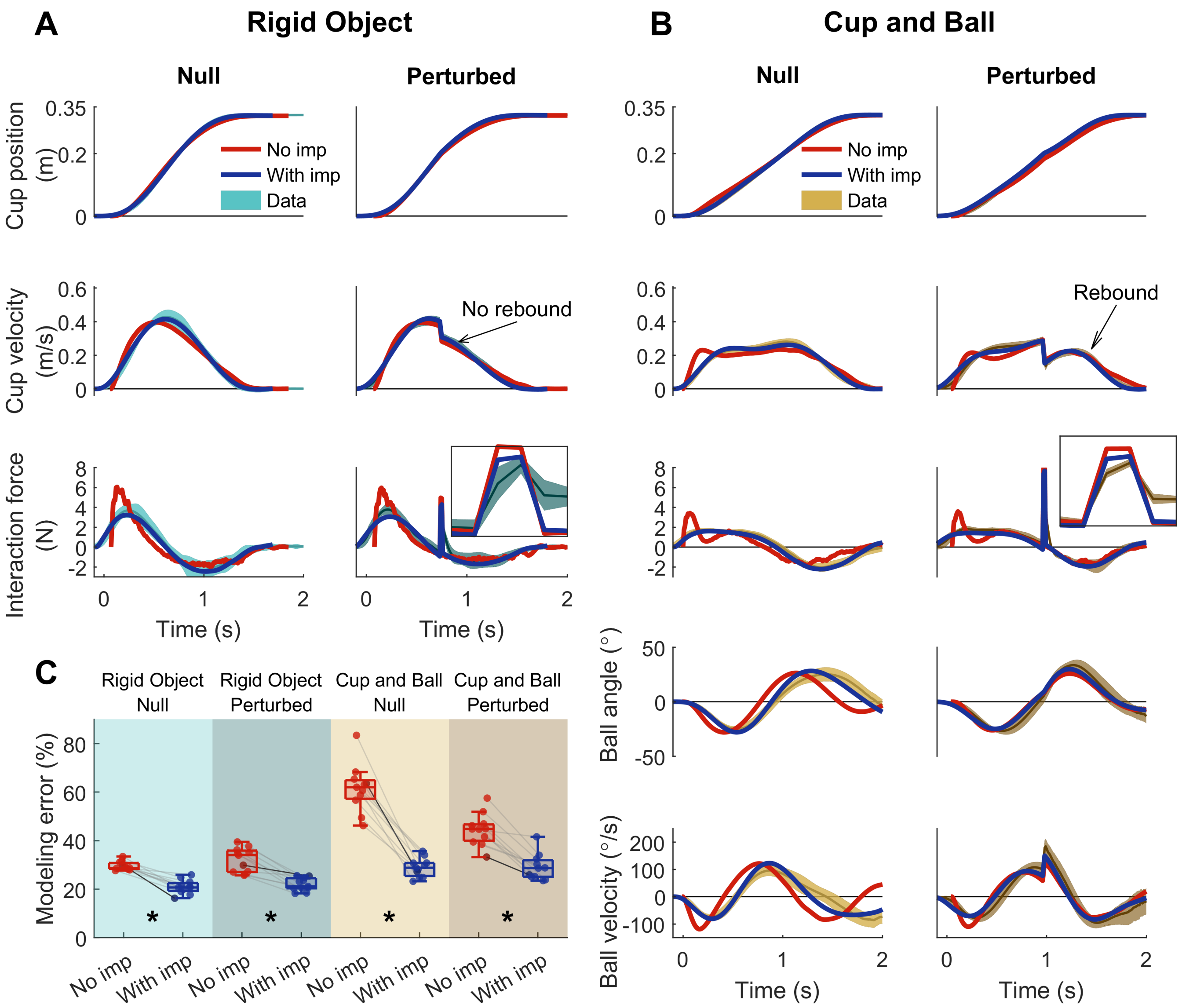

Fig S-7. Comparison of the model responses with participant #2's data.

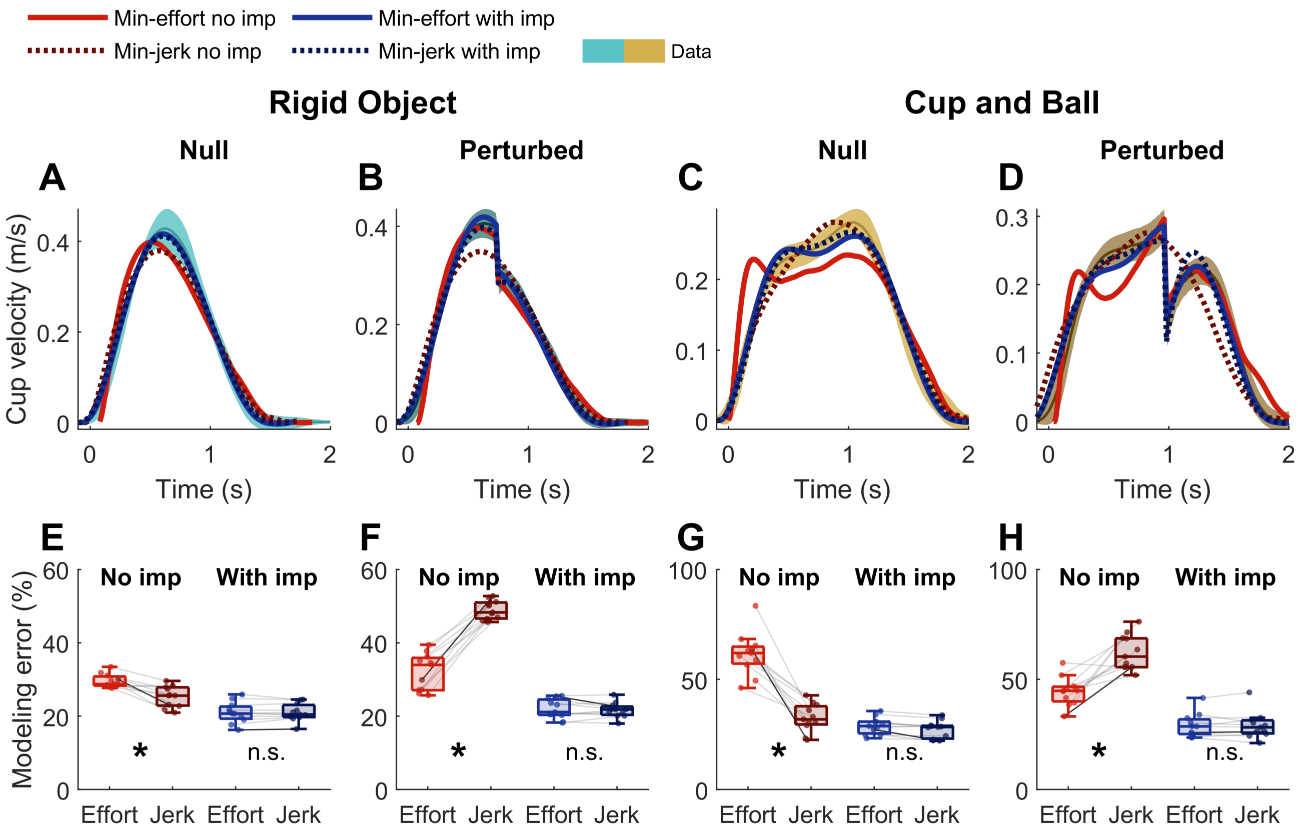

Fig S-8. Effects of the optimality criterion on the models fitted to participant #2's data

-2.0in0in

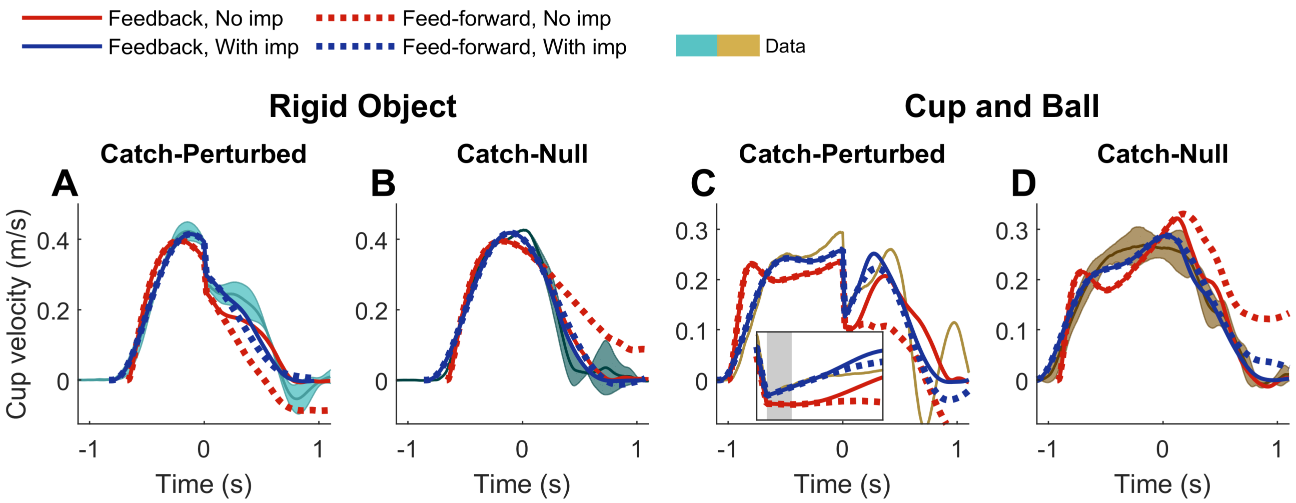

Fig S-9. Participant #2's catch trials and the the simulated behaviors.

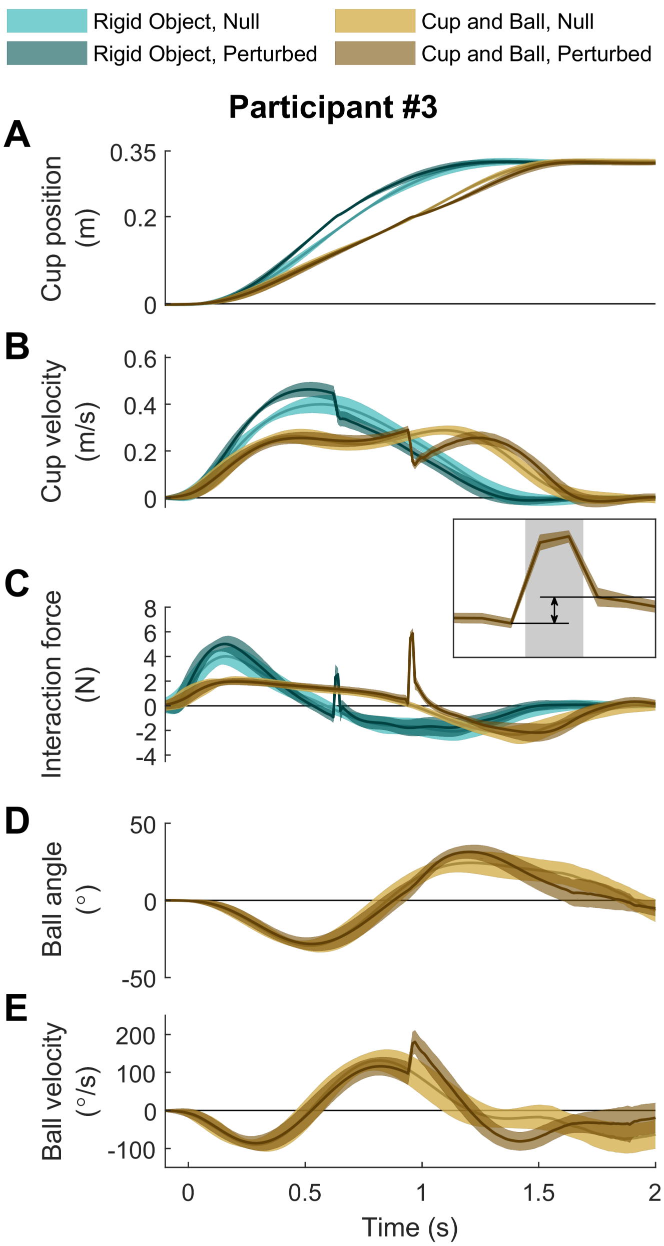

Fig S-10. Participant #3 behavior in the four blocks.

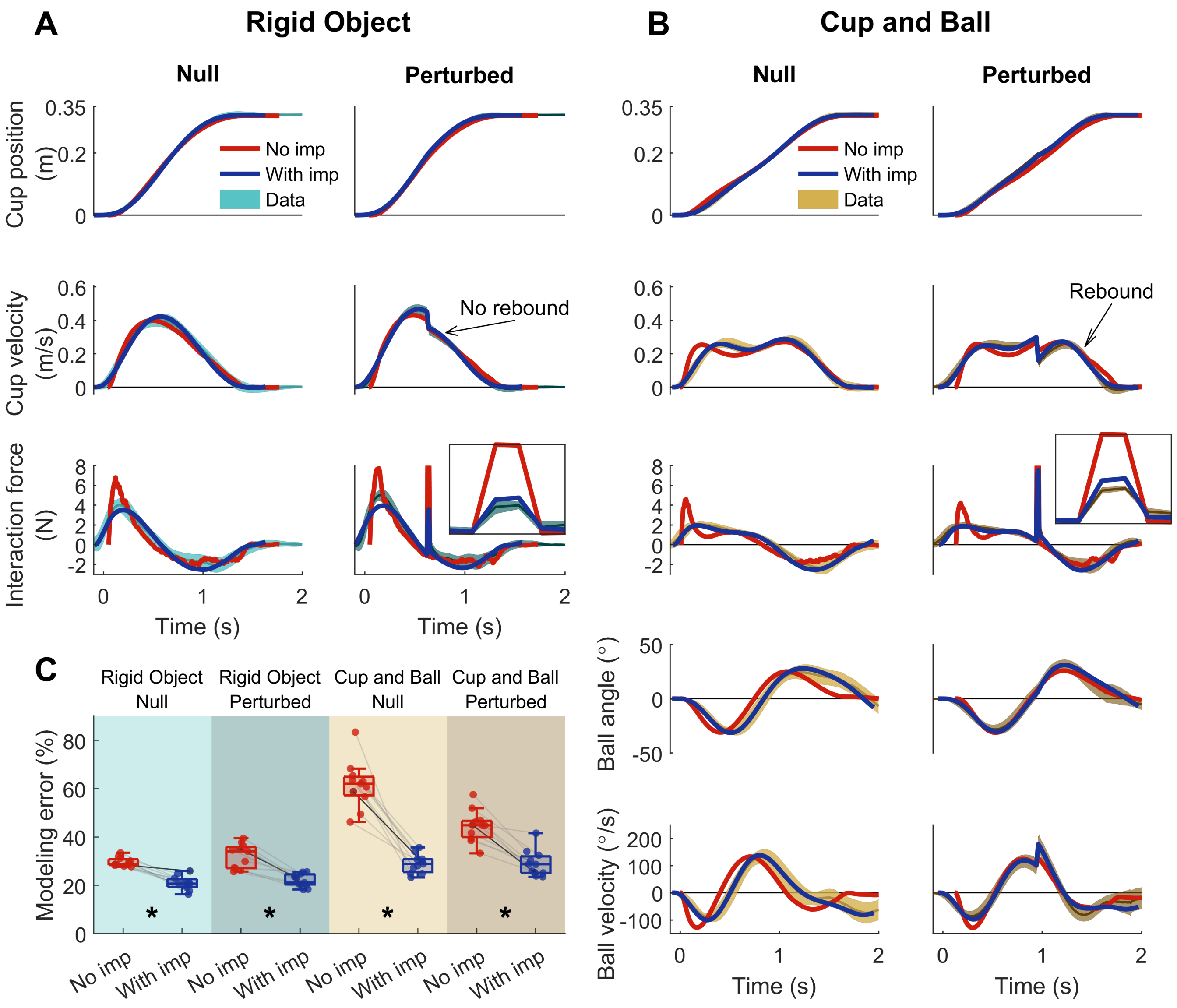

Fig S-11. Comparison of the model responses with participant #3's data.

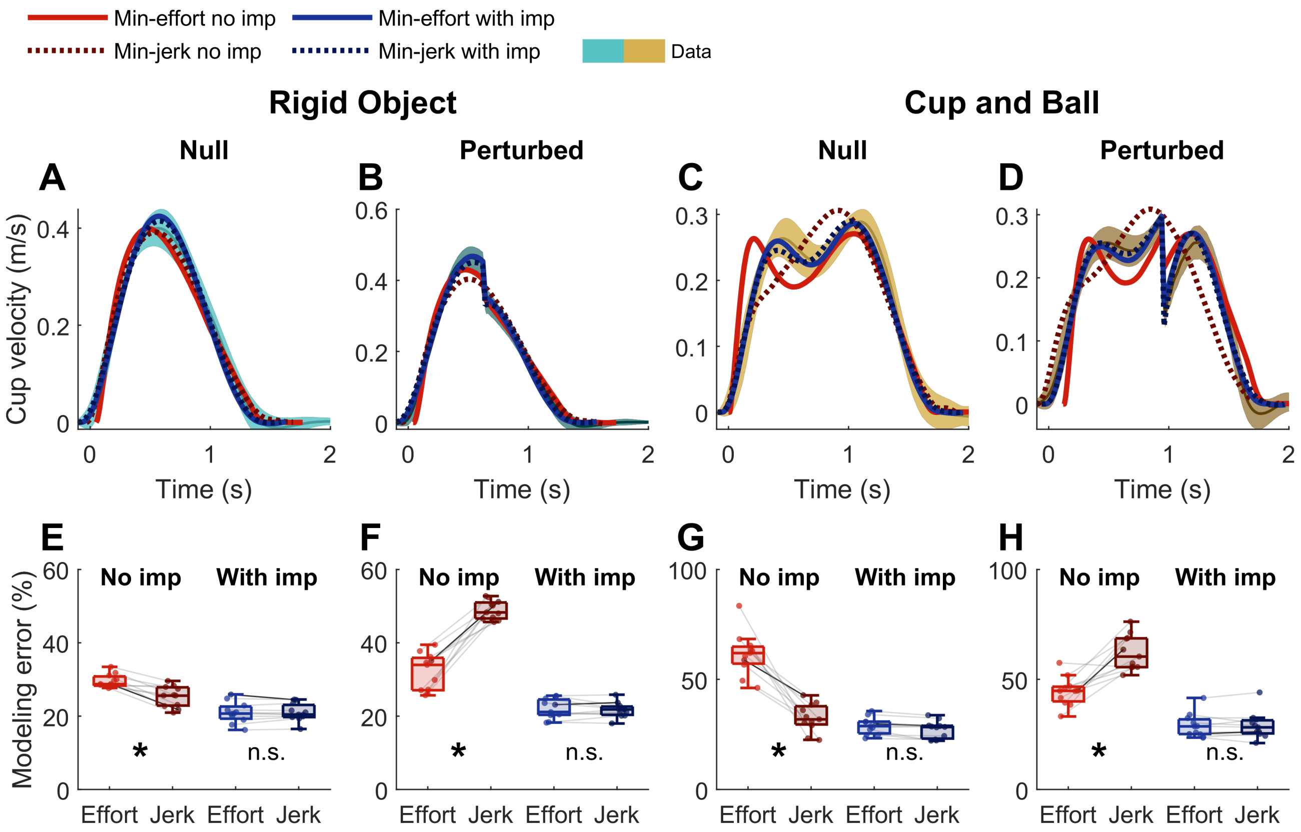

Fig S-12. Effects of the optimality criterion on the models fitted to participant #3's data

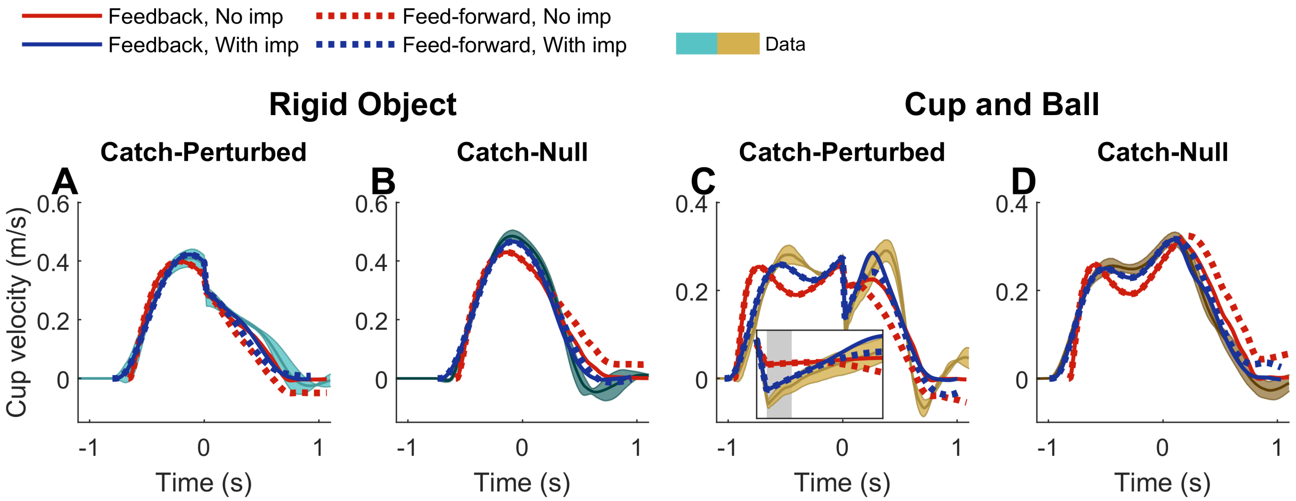

Fig S-13. Participant #3's catch trials and the the simulated behaviors.

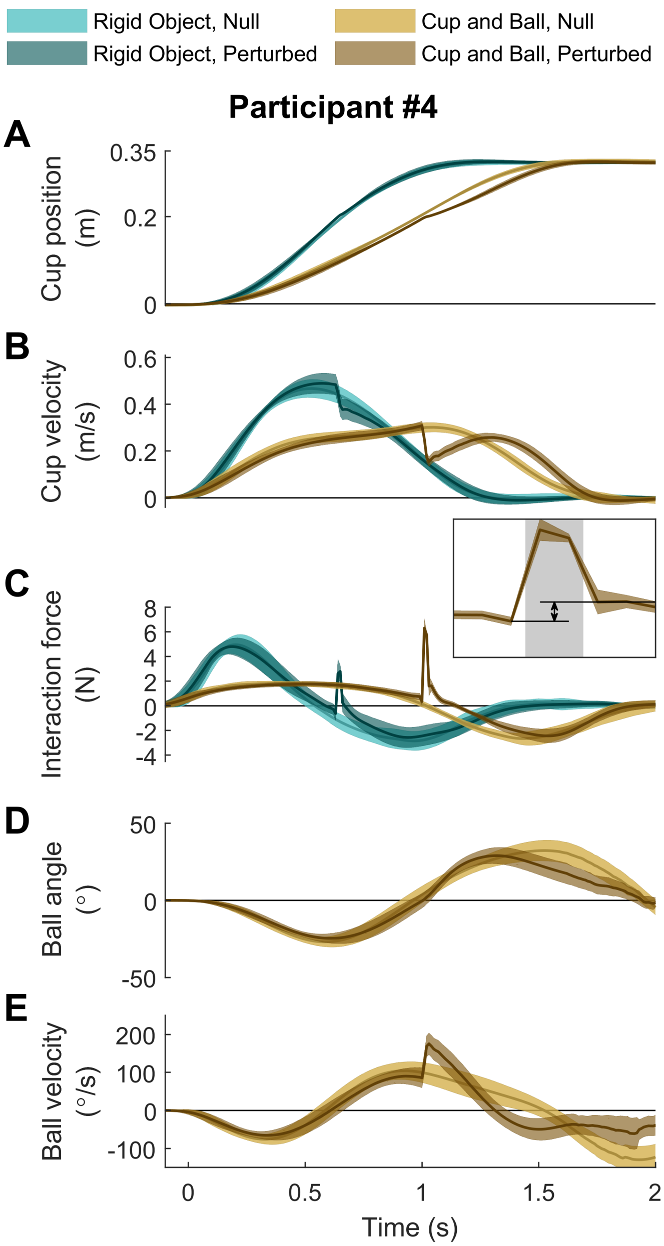

Fig S-14. Participant #4 behavior in the four blocks.

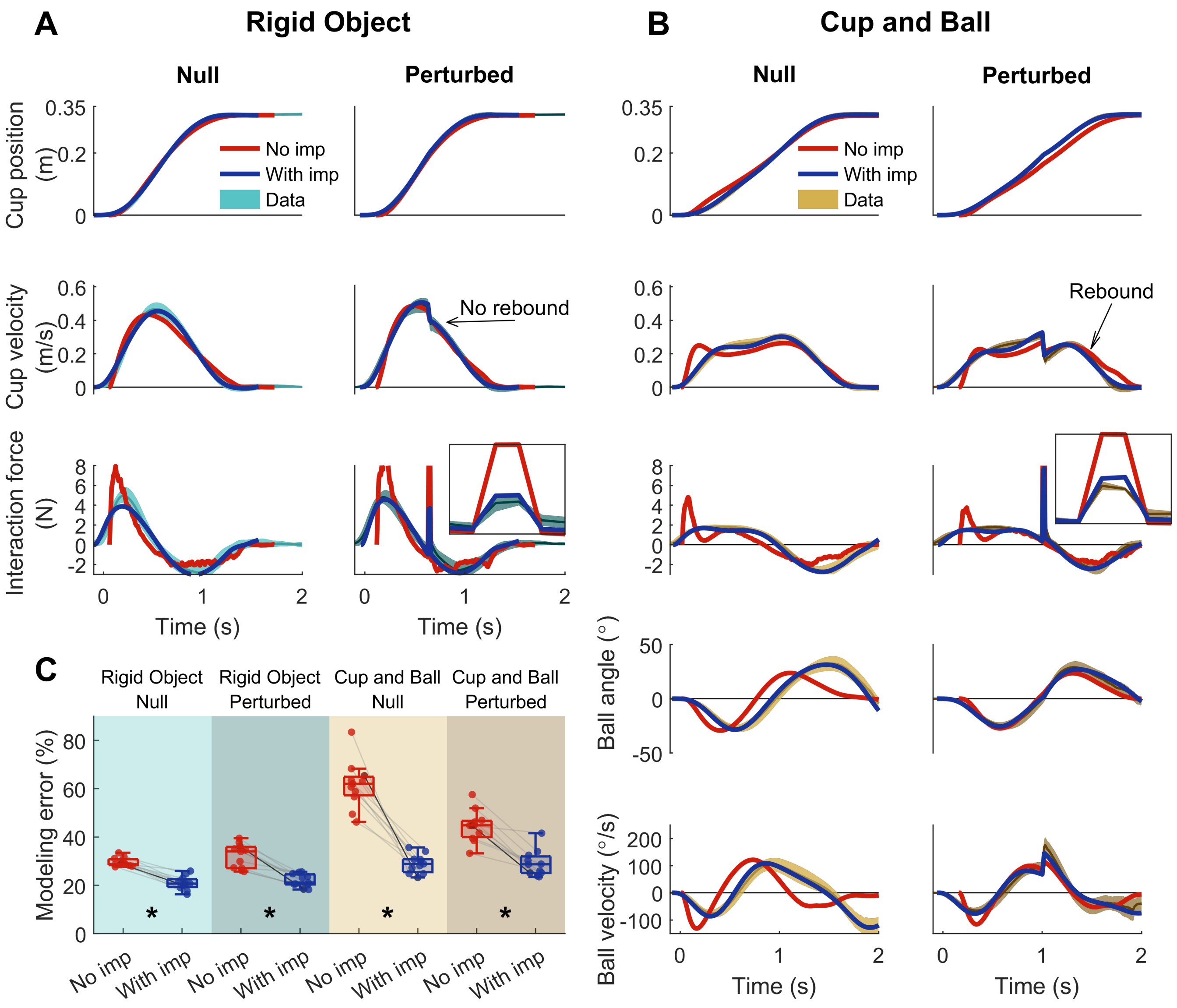

Fig S-15. Comparison of the model responses with participant #4's data.

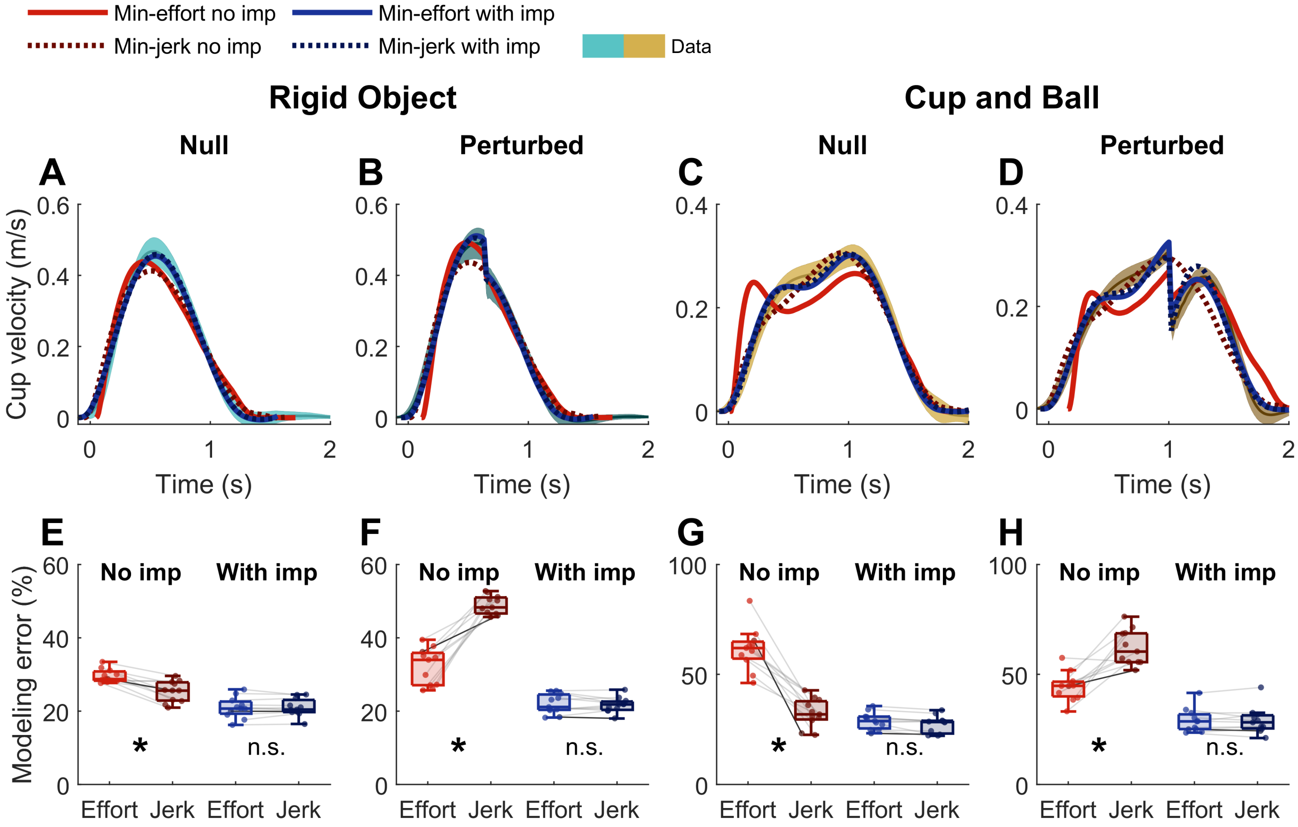

Fig S-16. Effects of the optimality criterion on the models fitted to participant #4's data

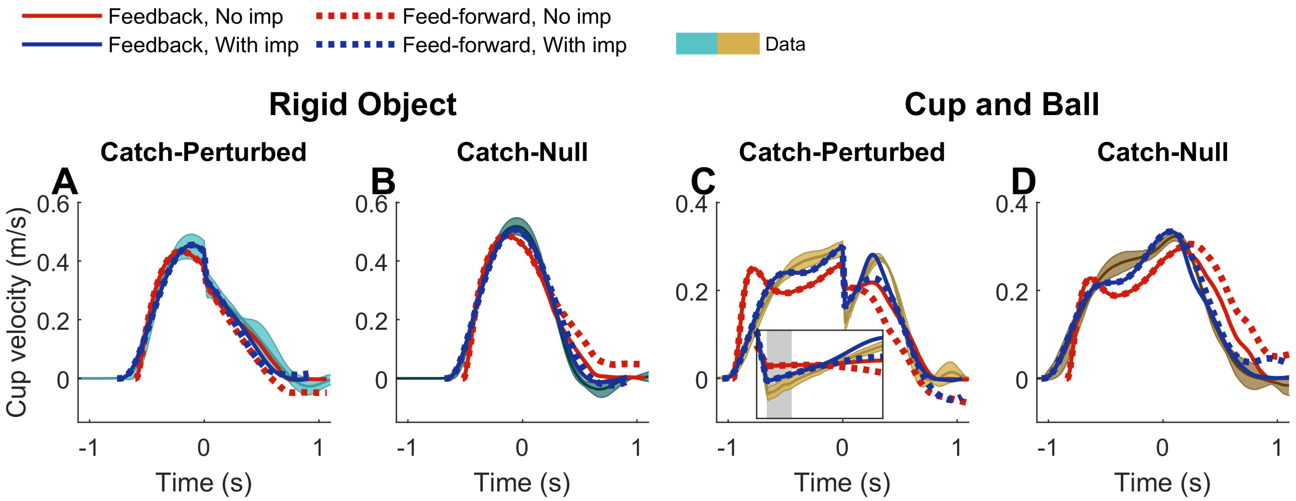

Fig S-17. Participant #4's catch trials and the the simulated behaviors.

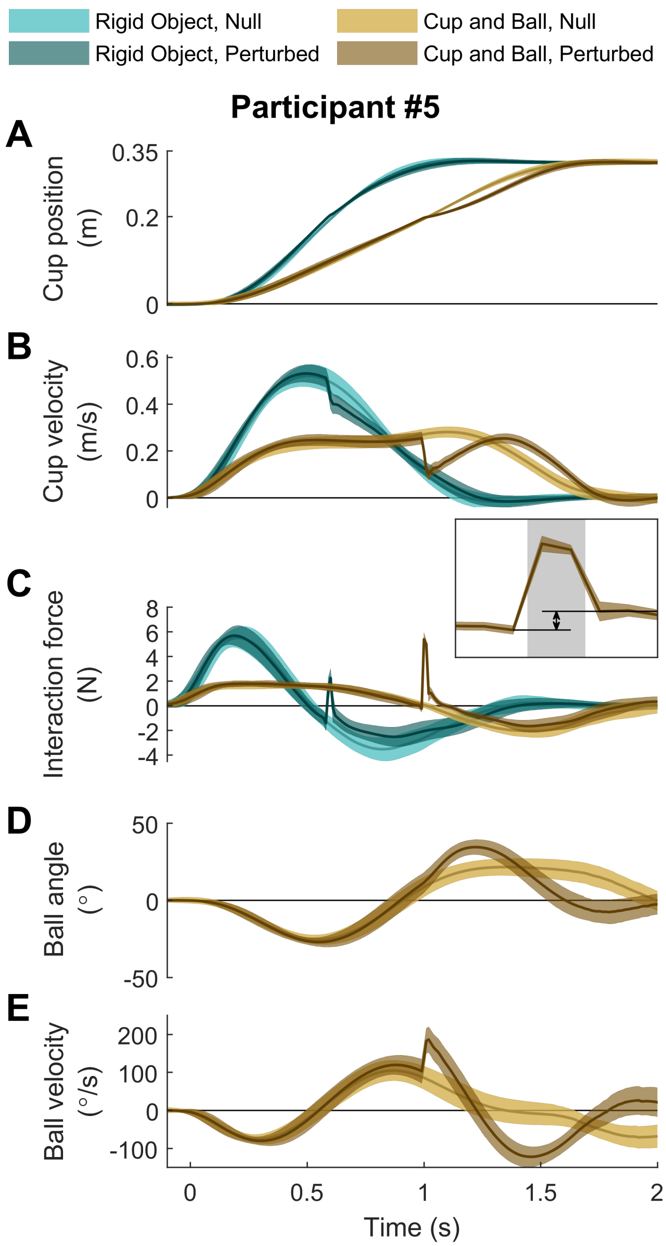

Fig S-18. Participant #5 behavior in the four blocks.

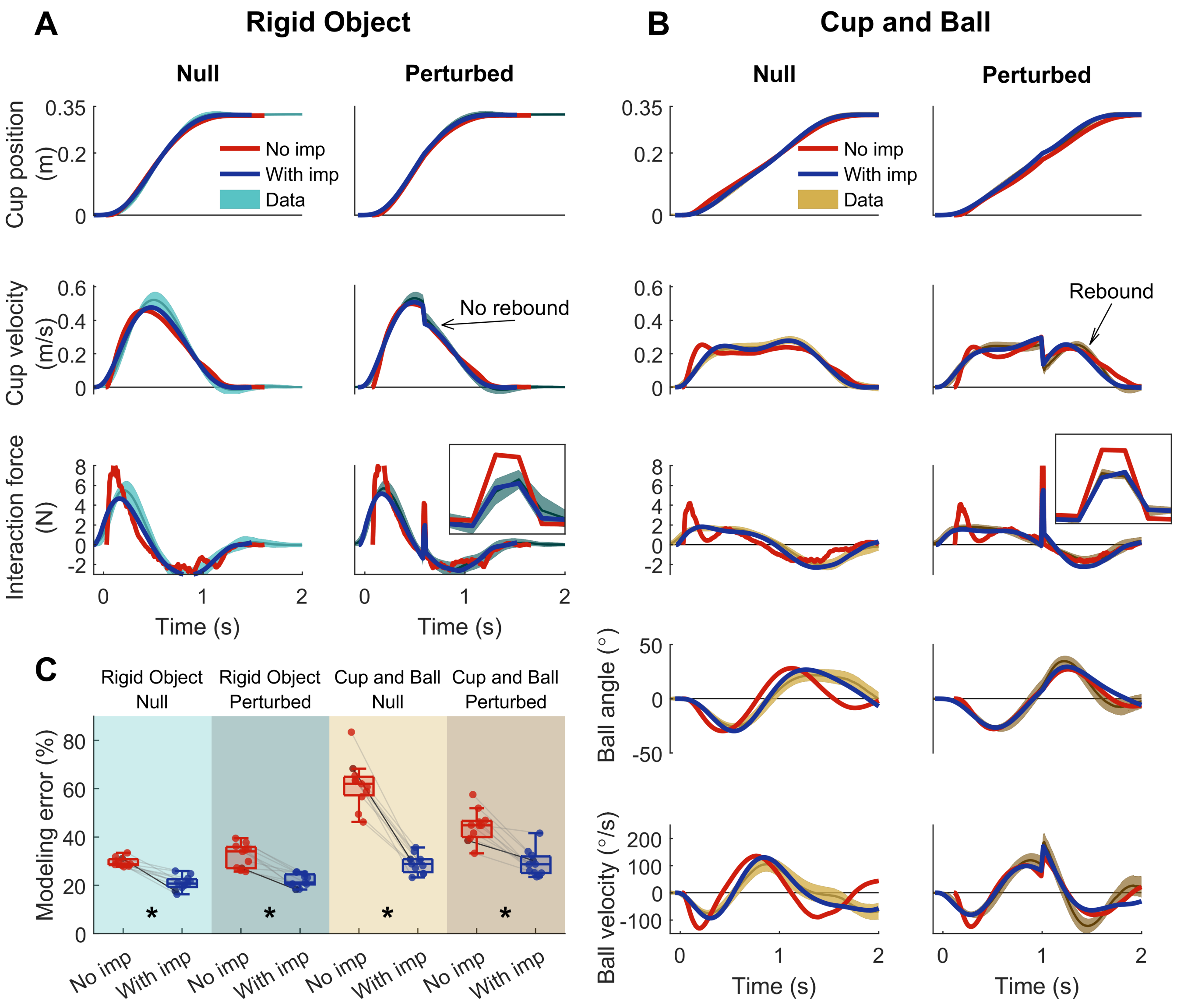

Fig S-19. Comparison of the model responses with participant #5's data.

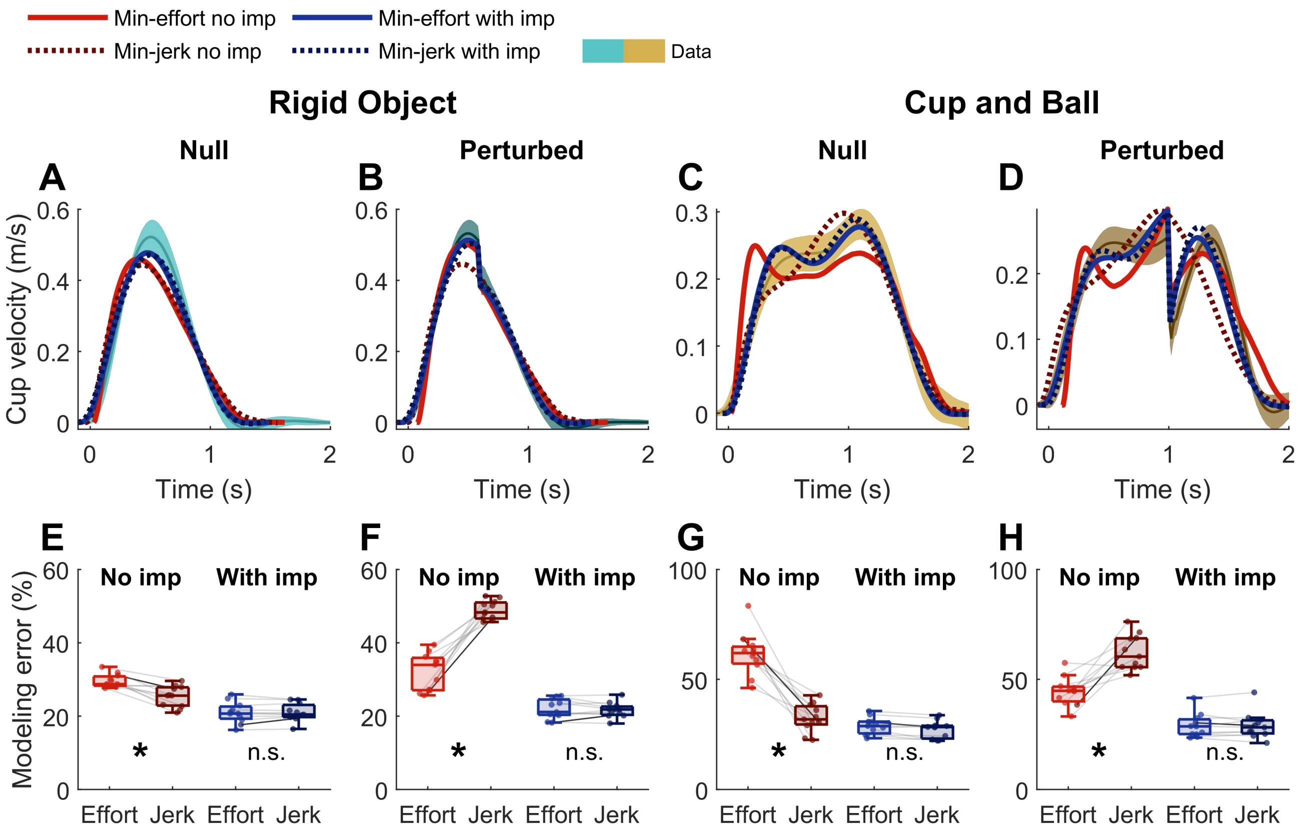

Fig S-20. Effects of the optimality criterion on the models fitted to participant #5's data

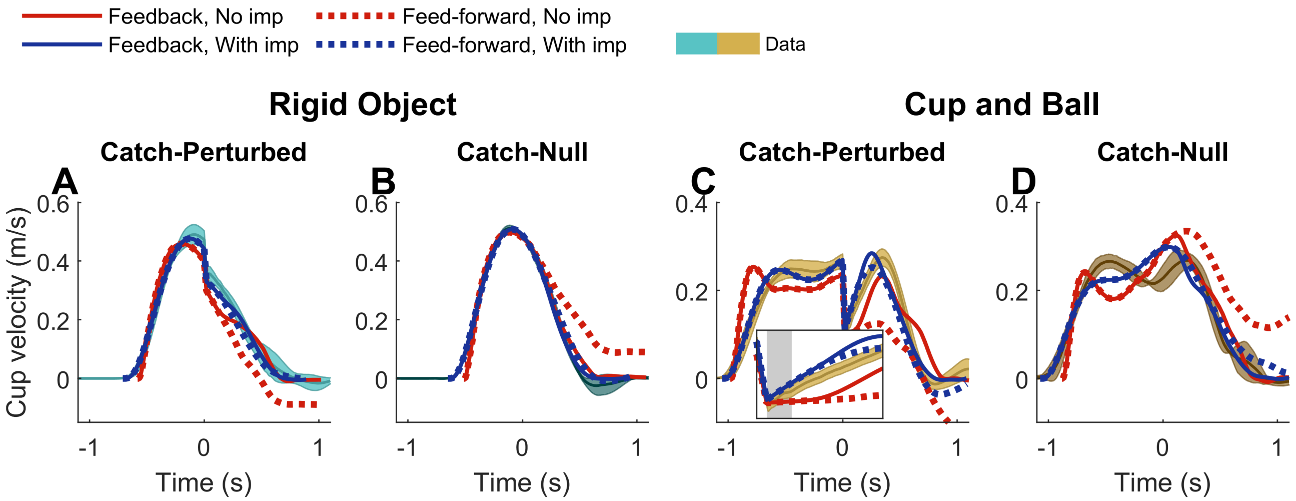

Fig S-21. Participant #5's catch trials and the the simulated behaviors.

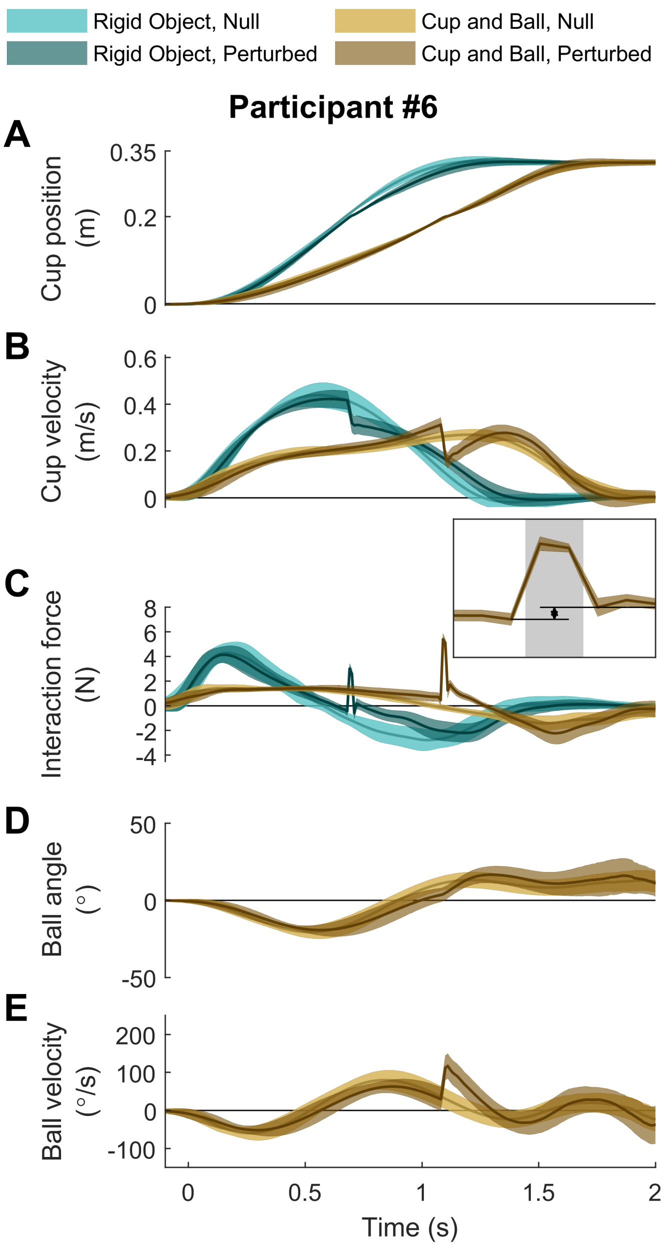

Fig S-22. Participant #6 behavior in the four blocks.

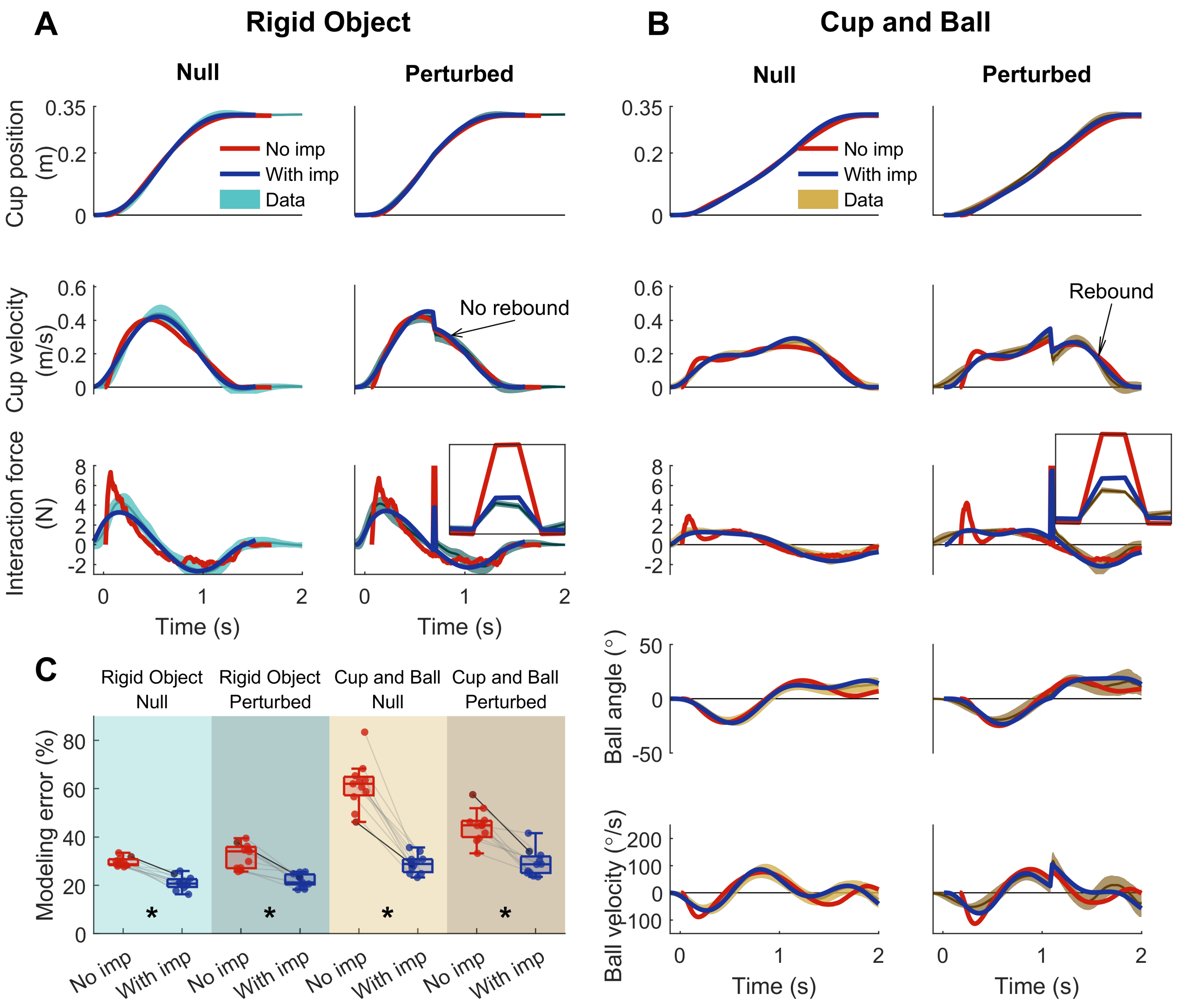

Fig S-23. Comparison of the model responses with participant #6's data.

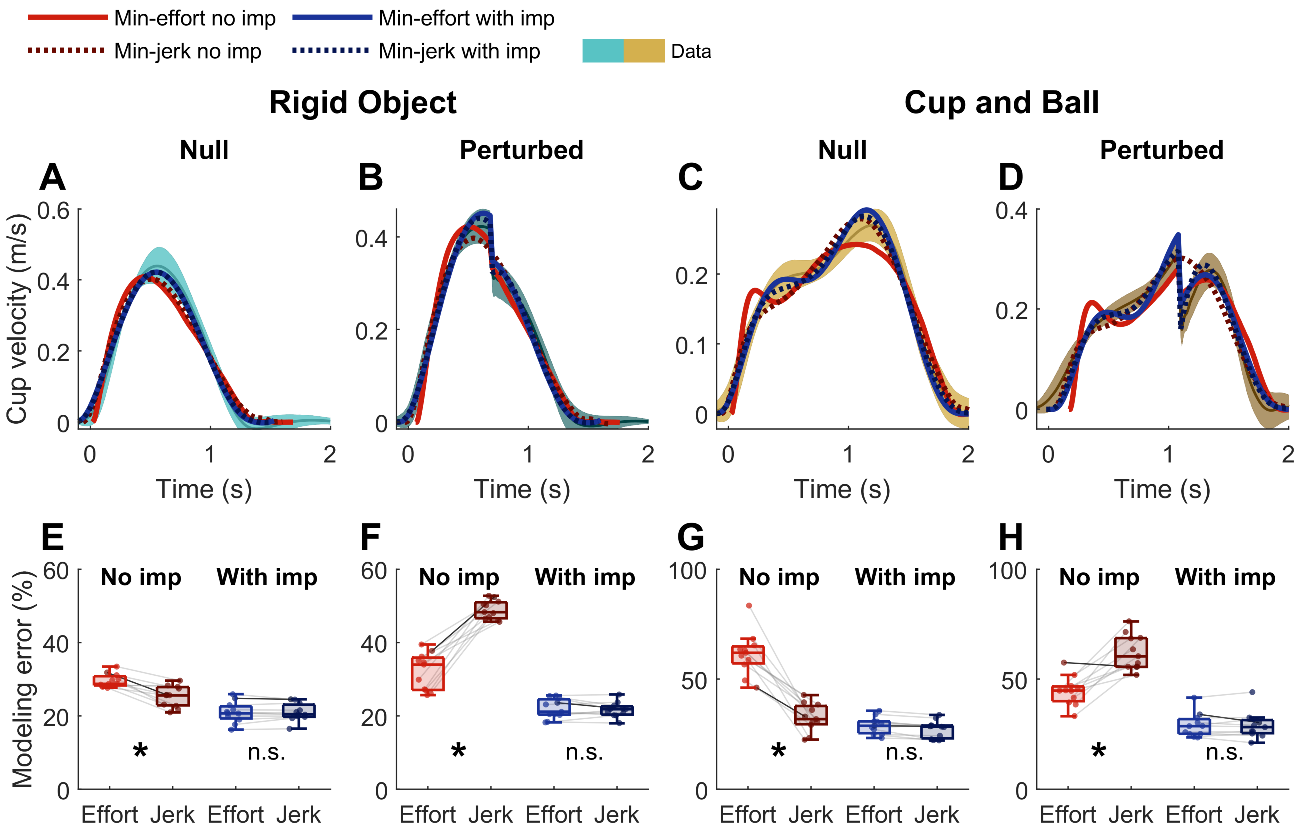

Fig S-24. Effects of the optimality criterion on the models fitted to participant #6's data

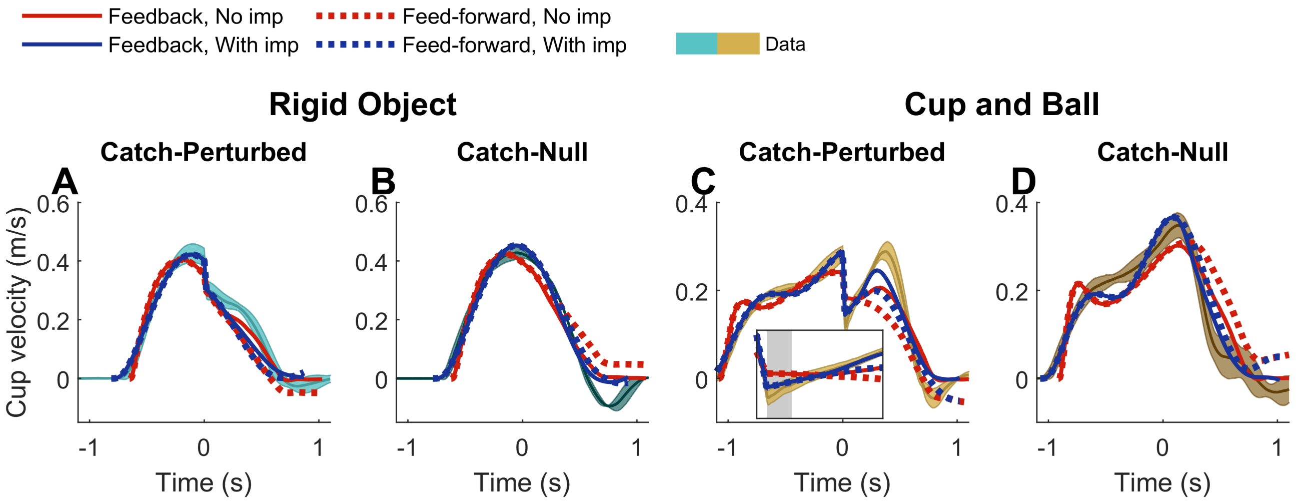

Fig S-25. Participant #6's catch trials and the the simulated behaviors.

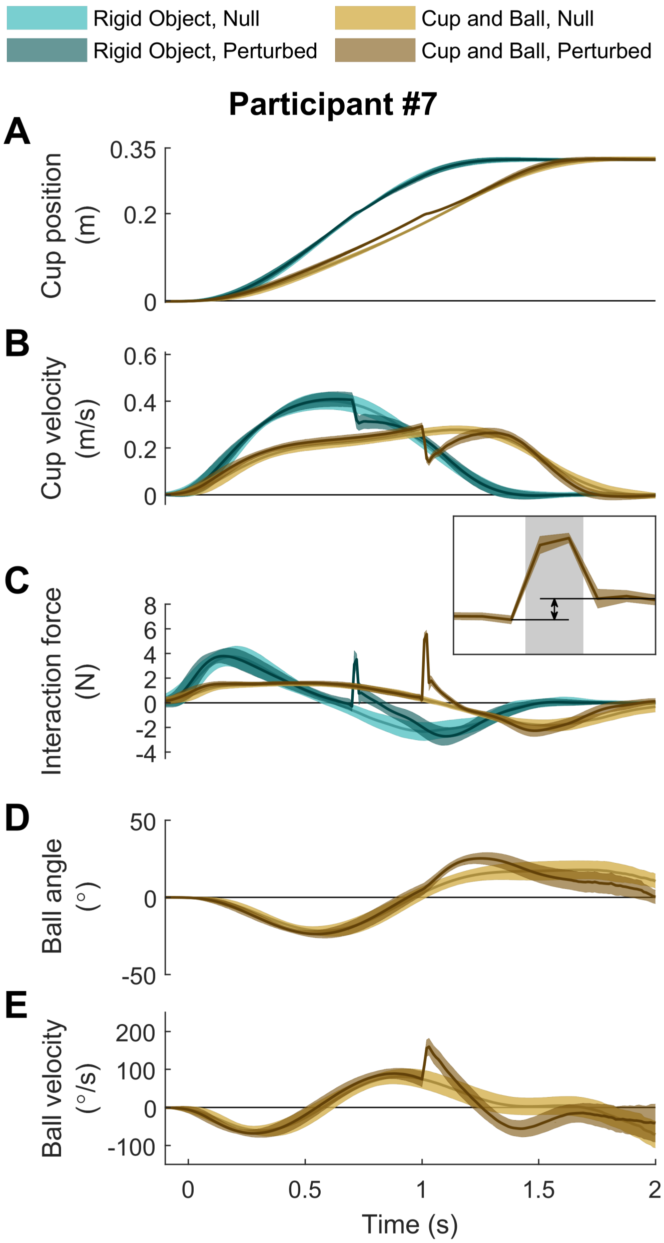

Fig S-26. Participant #7 behavior in the four blocks.

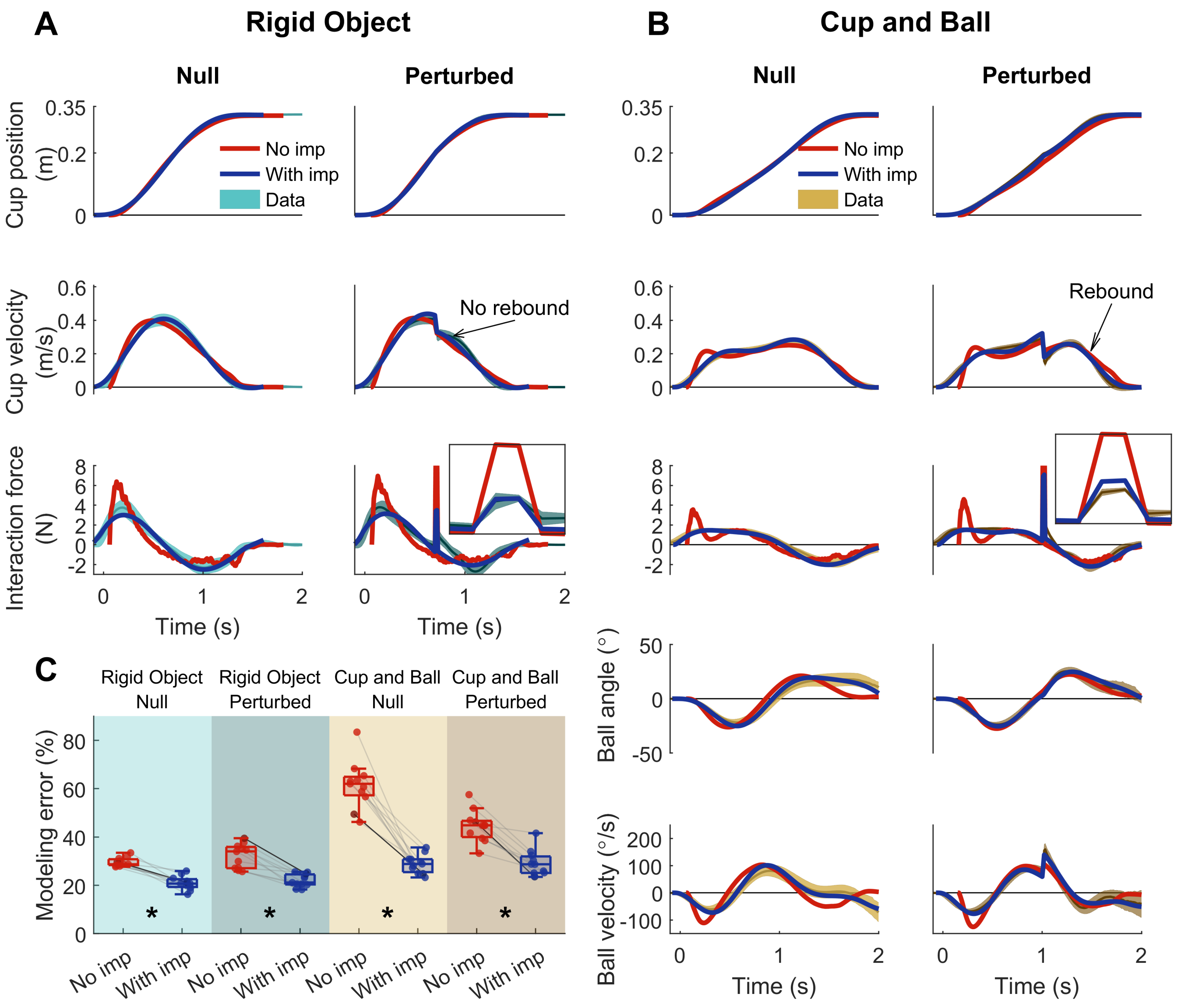

Fig S-27. Comparison of the model responses with participant #7's data.

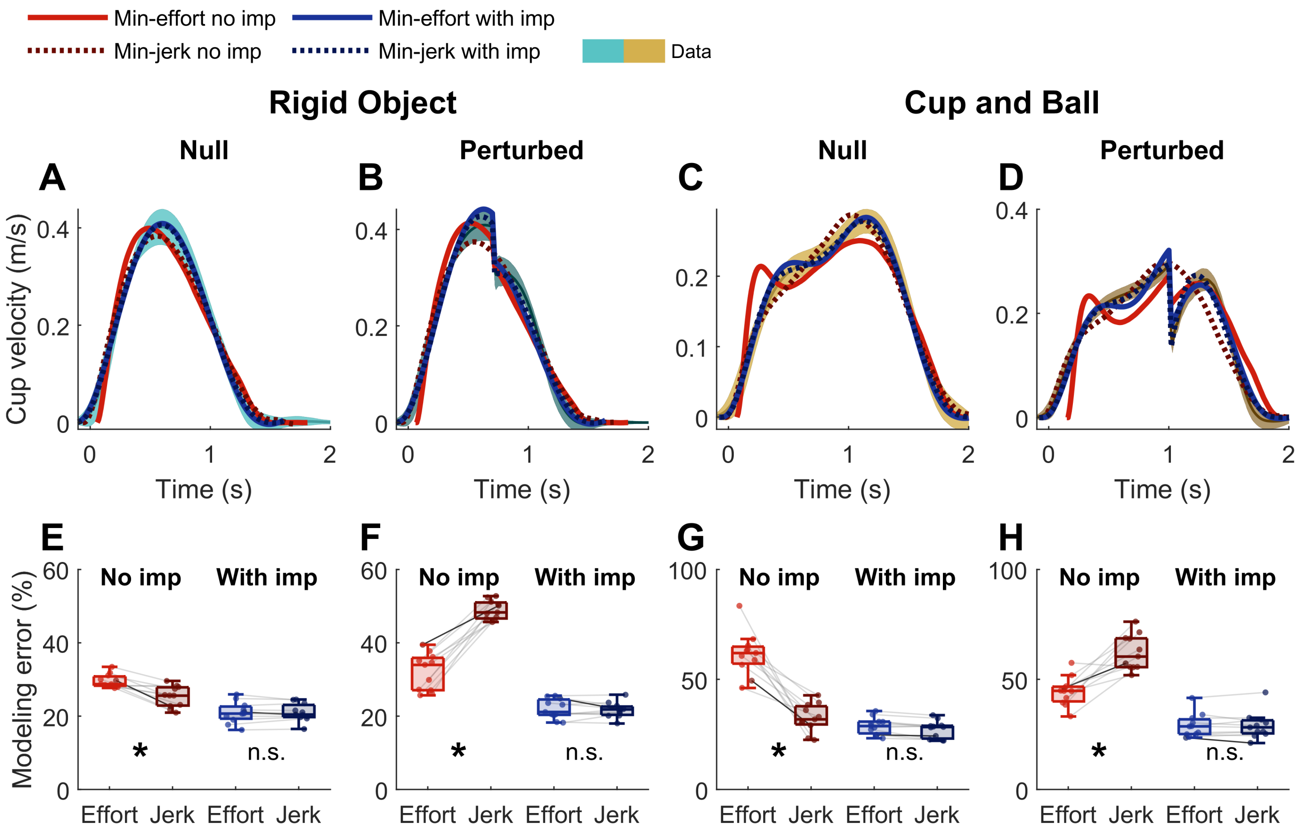

Fig S-28. Effects of the optimality criterion on the models fitted to participant #7's data

-2.0in0in

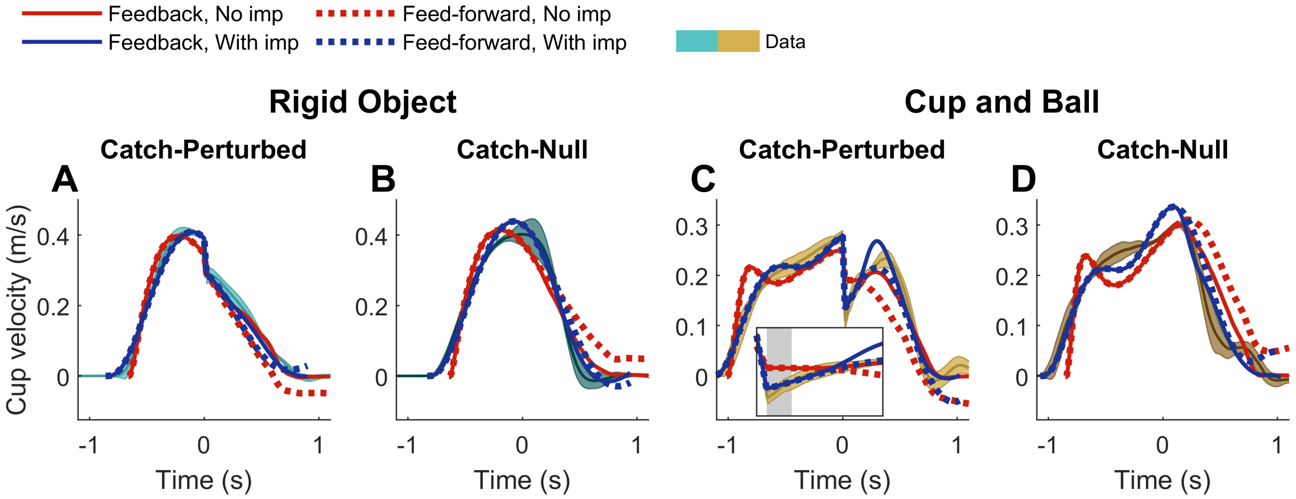

Fig S-29. Participant #7's catch trials and the the simulated behaviors.

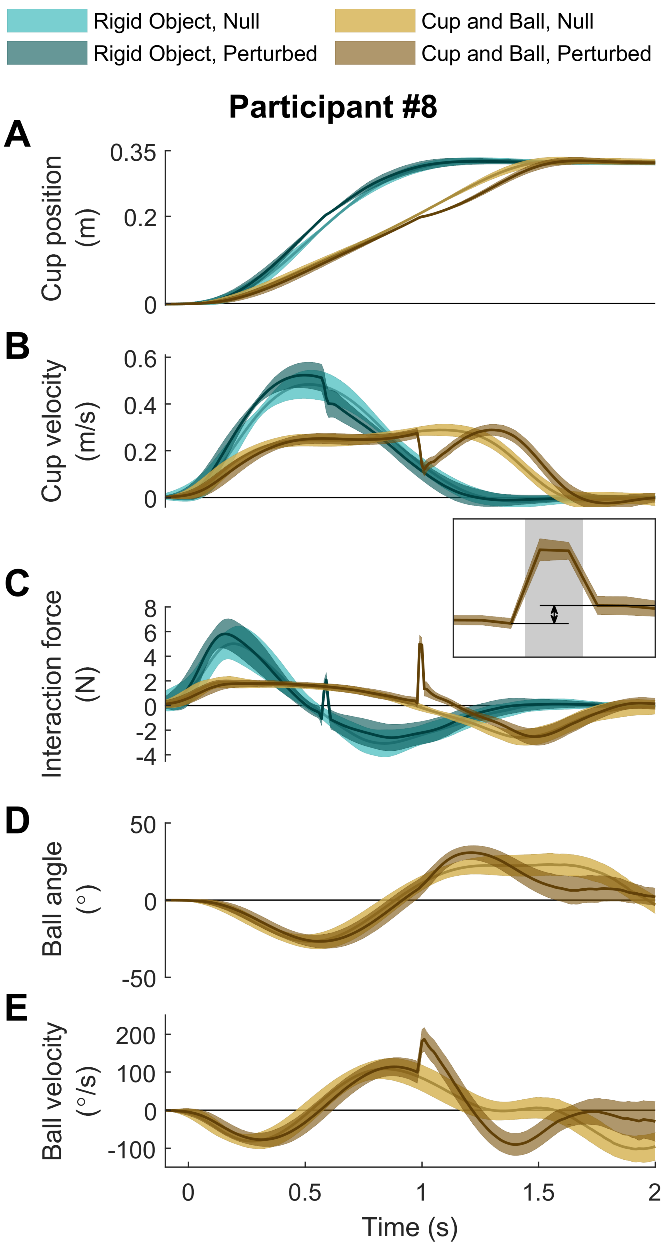

Fig S-30. Participant #8 behavior in the four blocks.

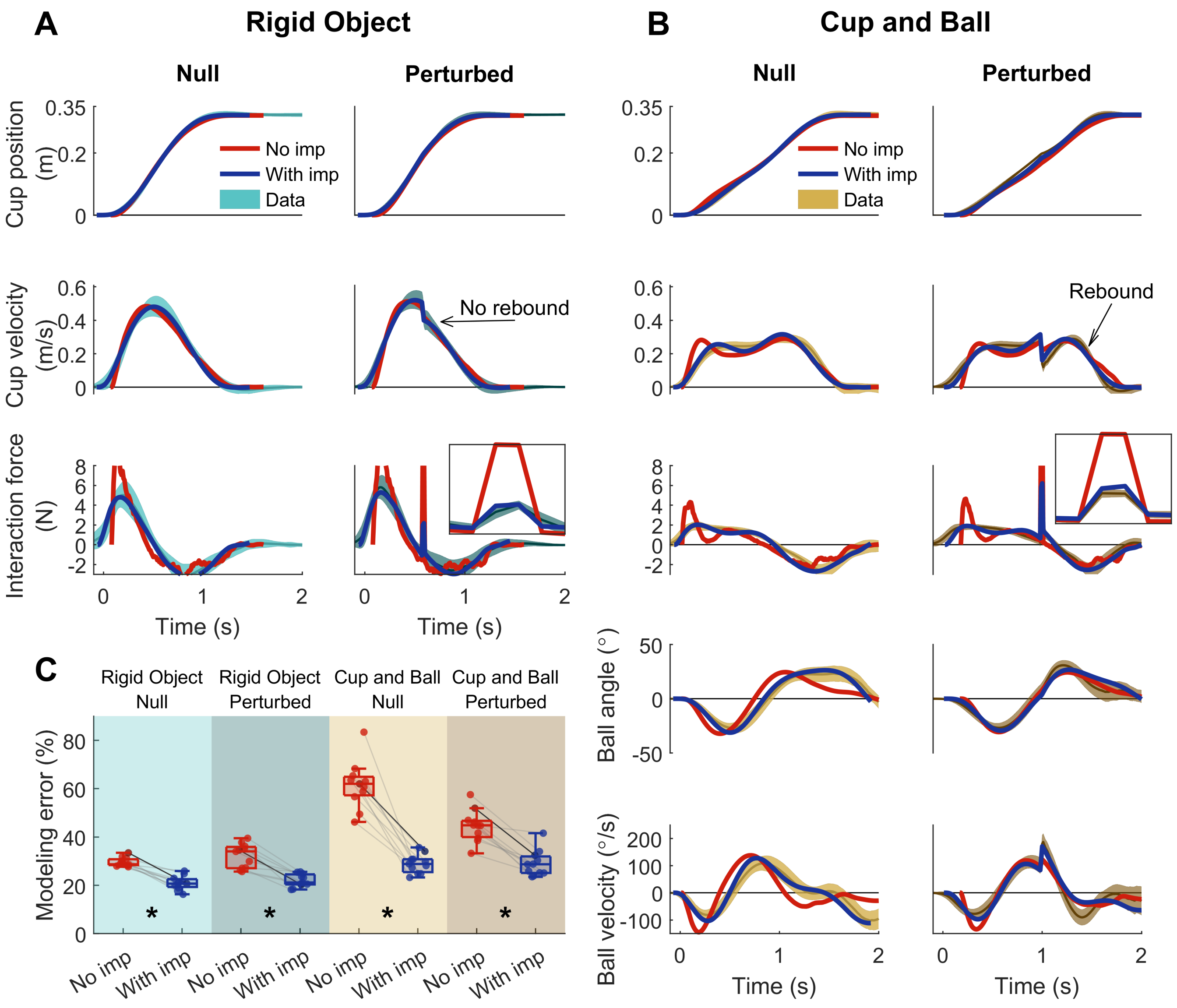

Fig S-31. Comparison of the model responses with participant #8's data.

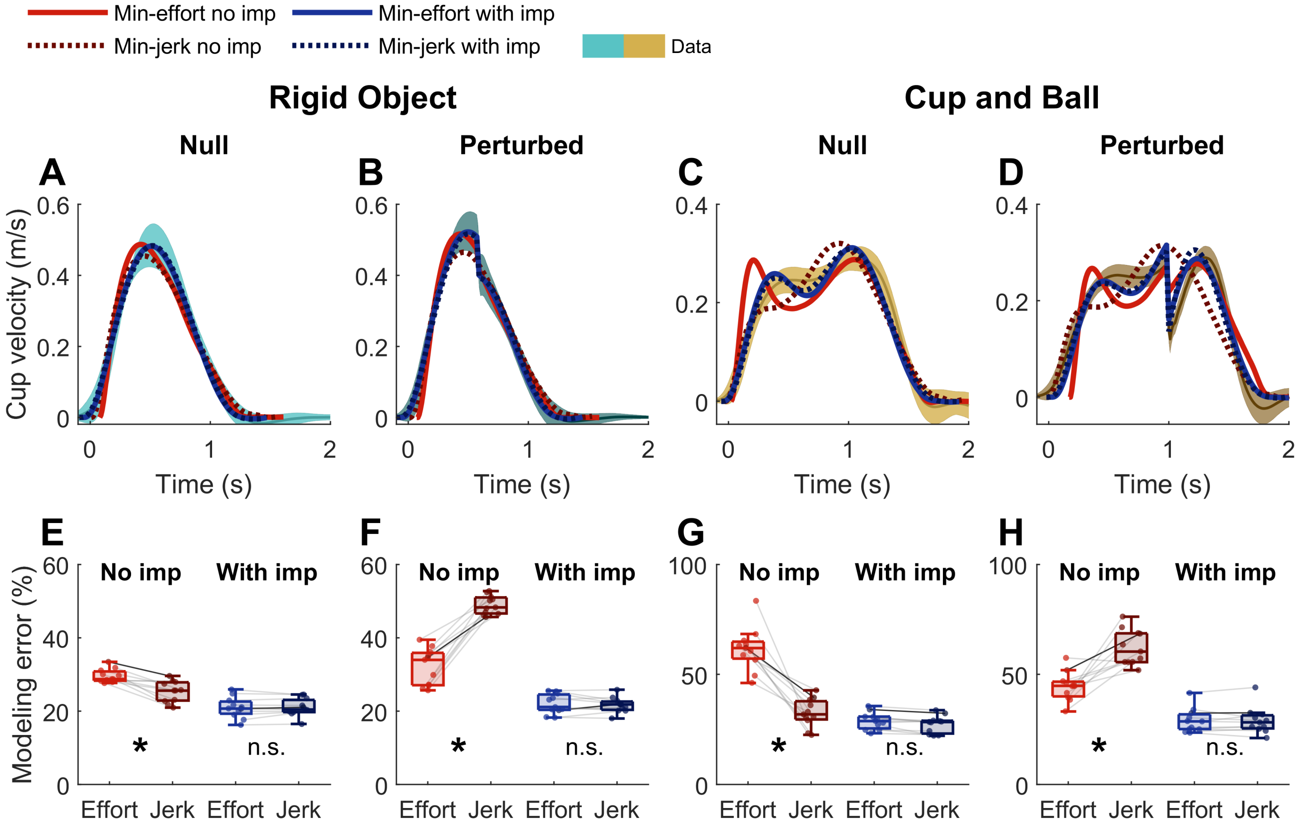

Fig S-32. Effects of the optimality criterion on the models fitted to participant #8's data

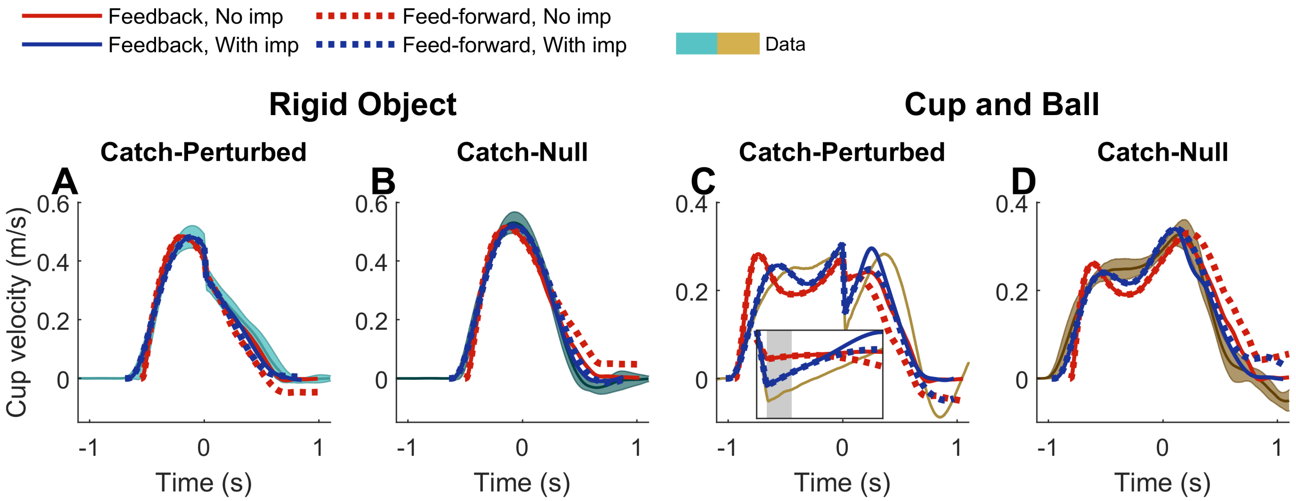

Fig S-33. Participant #8's catch trials and the the simulated behaviors.

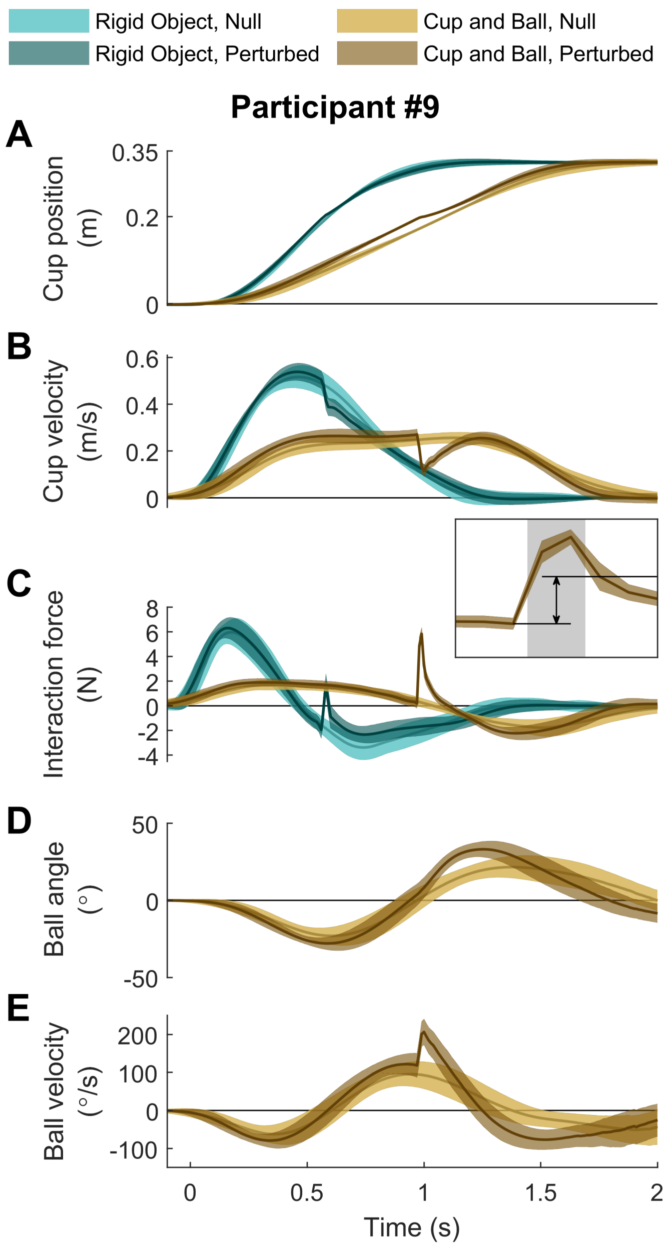

Fig S-34. Participant #9 behavior in the four blocks.

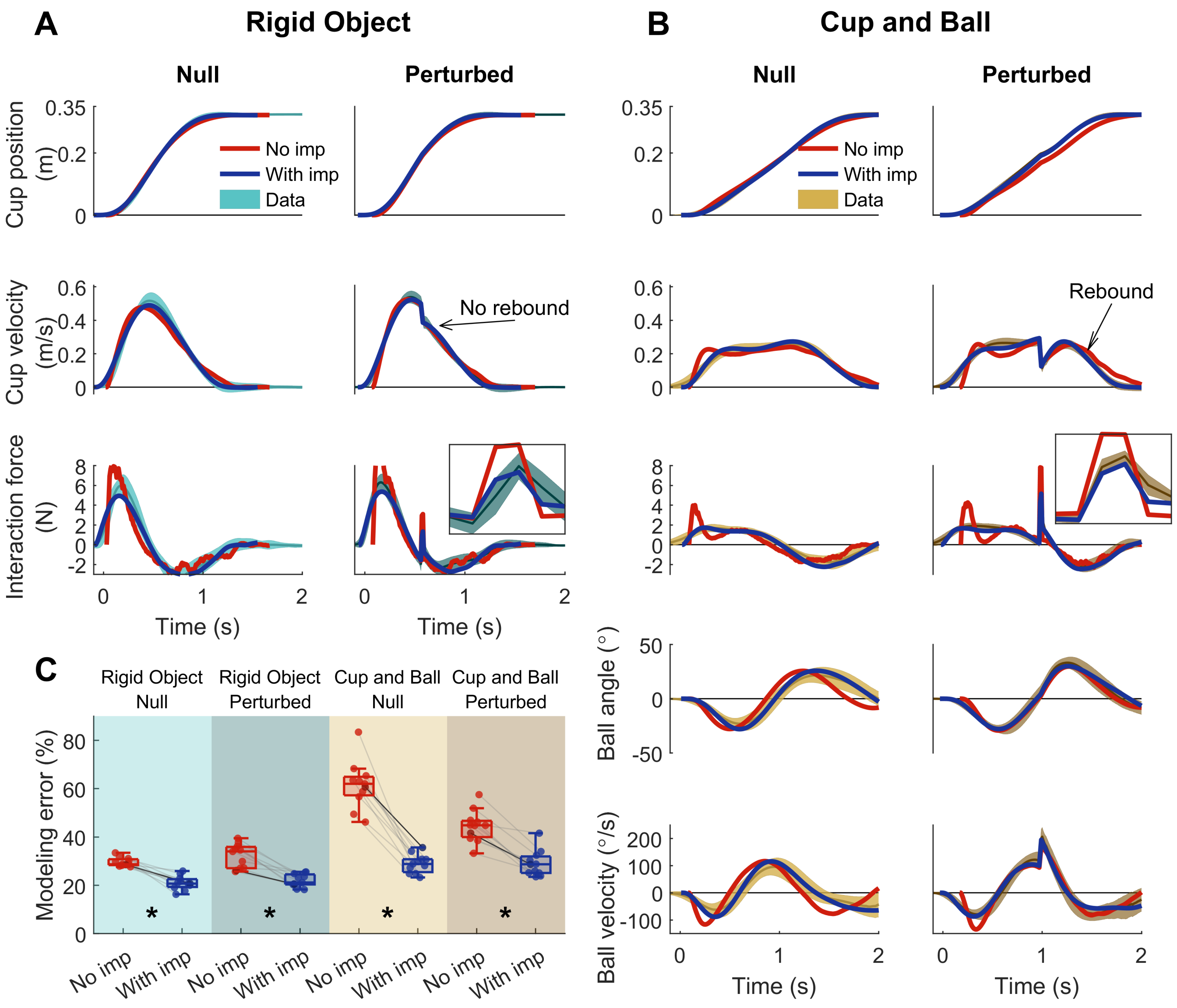

Fig S-35. Comparison of the model responses with participant #9's data.

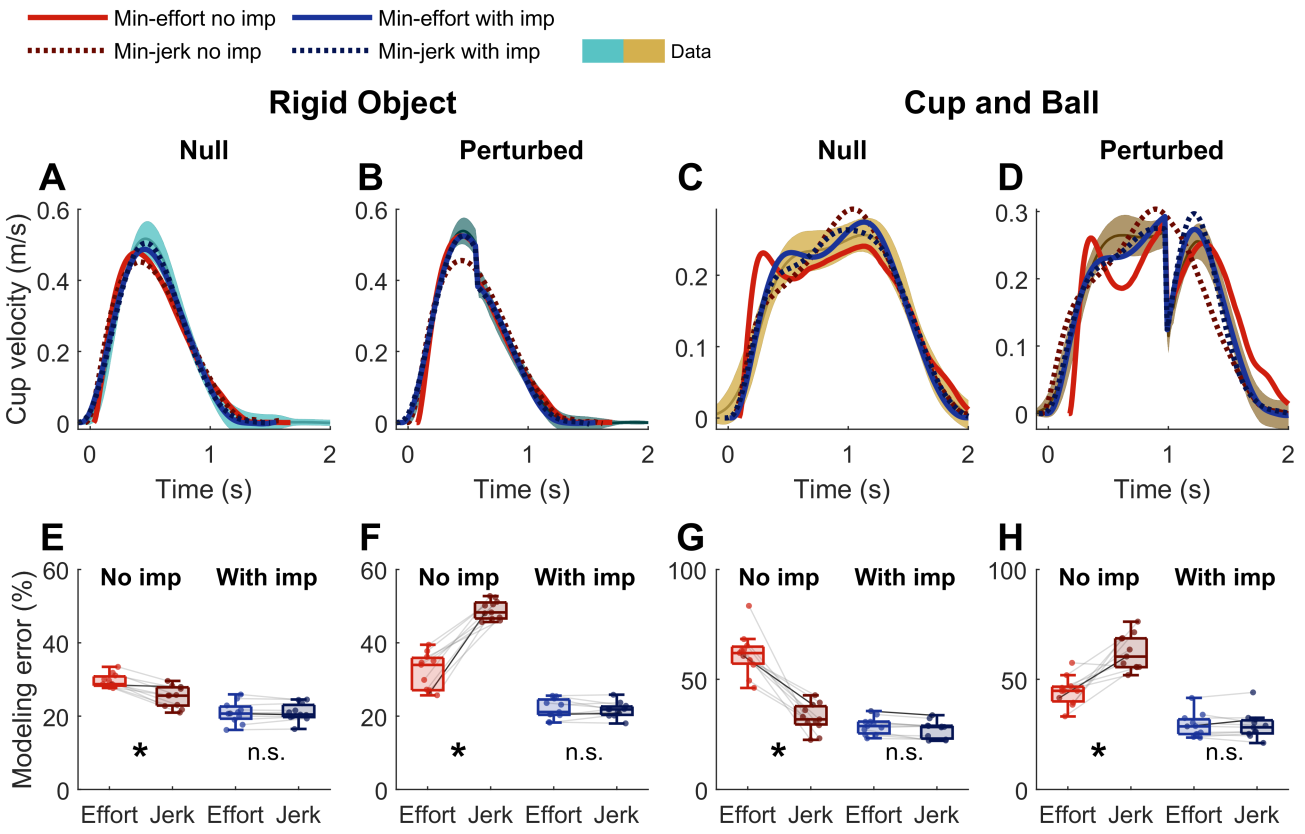

Fig S-36. Effects of the optimality criterion on the models fitted to participant #9's data

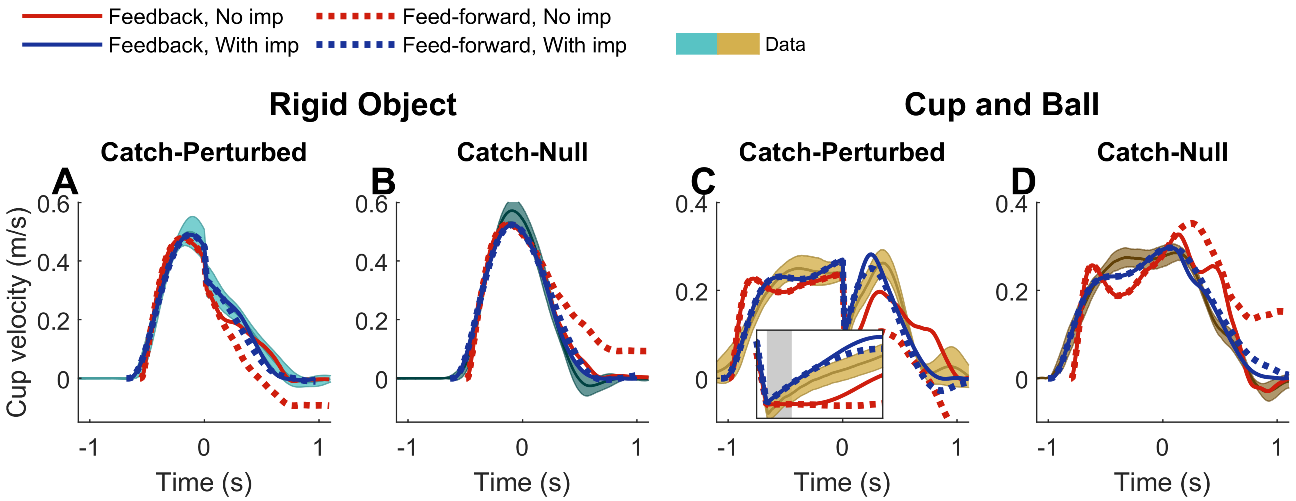

Fig S-37. Participant #9's catch trials and the the simulated behaviors.

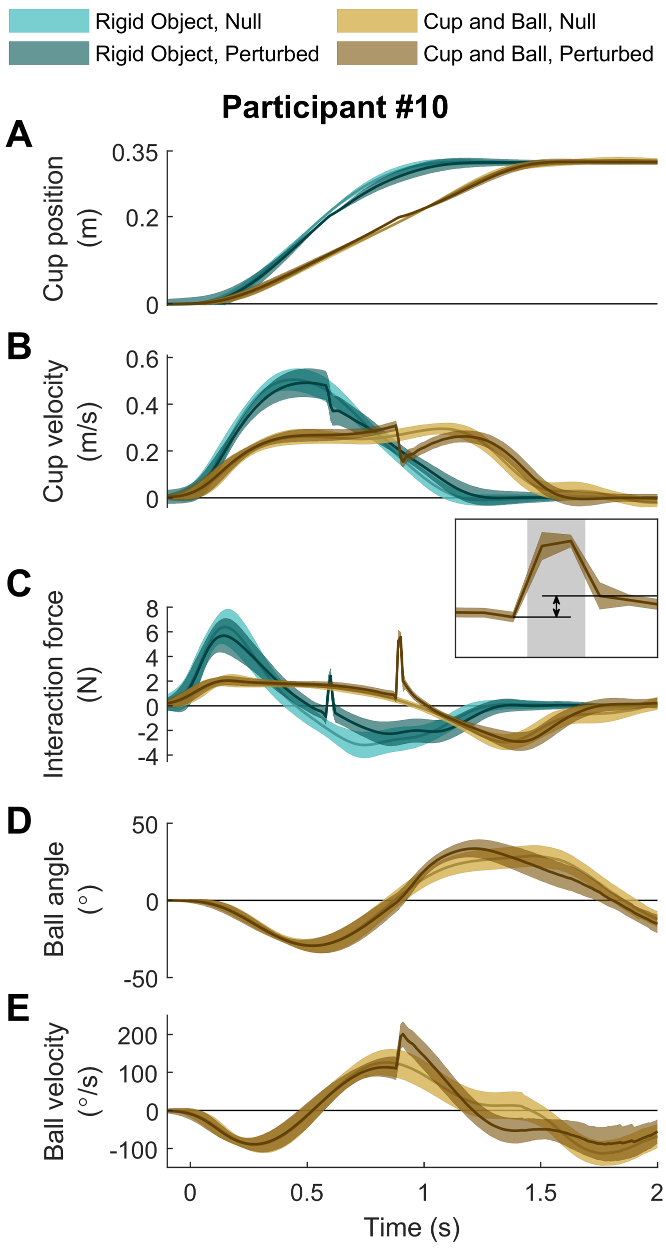

Fig S-38. Participant #10 behavior in the four blocks.

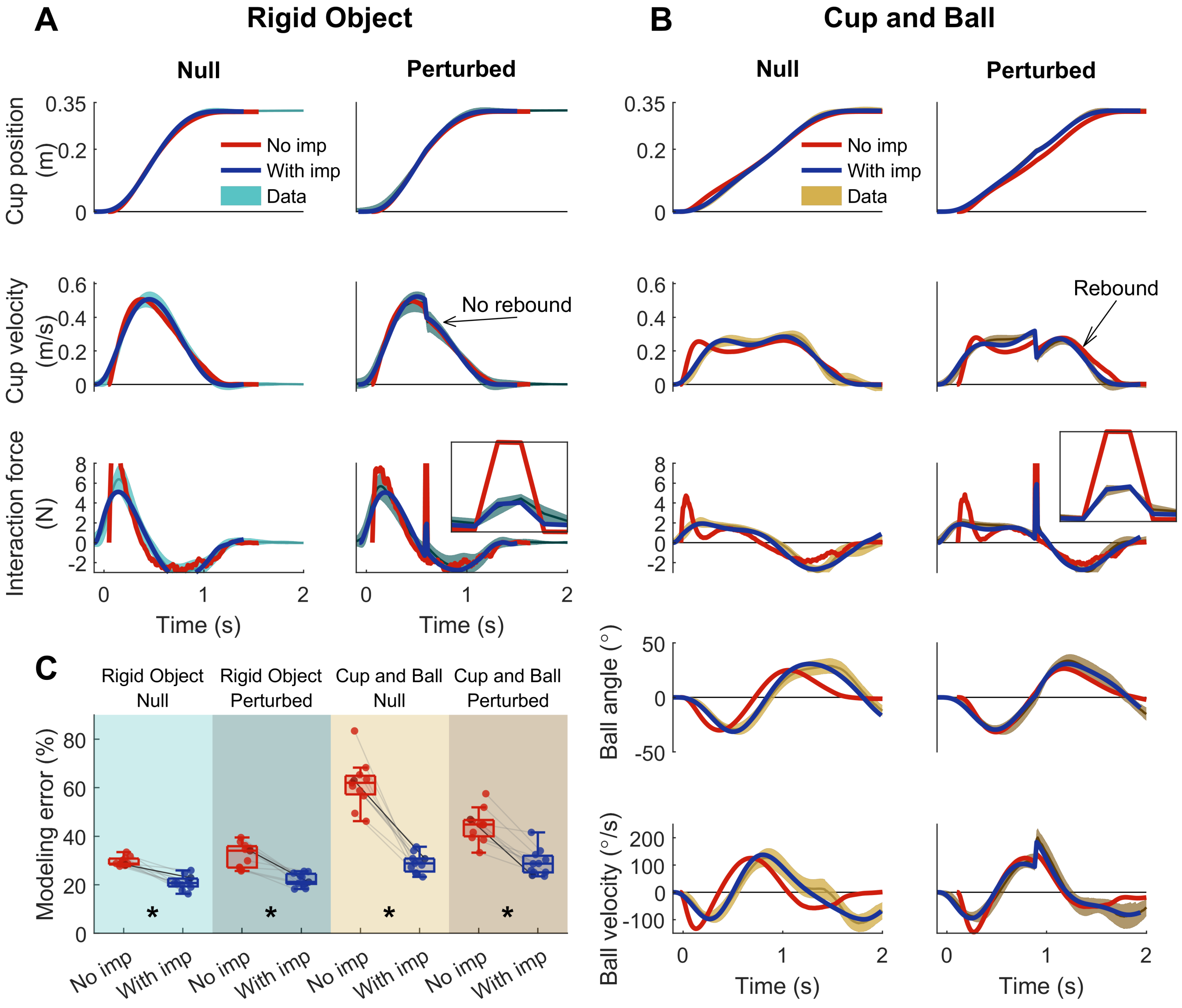

Fig S-39. Comparison of the model responses with participant #10's data.

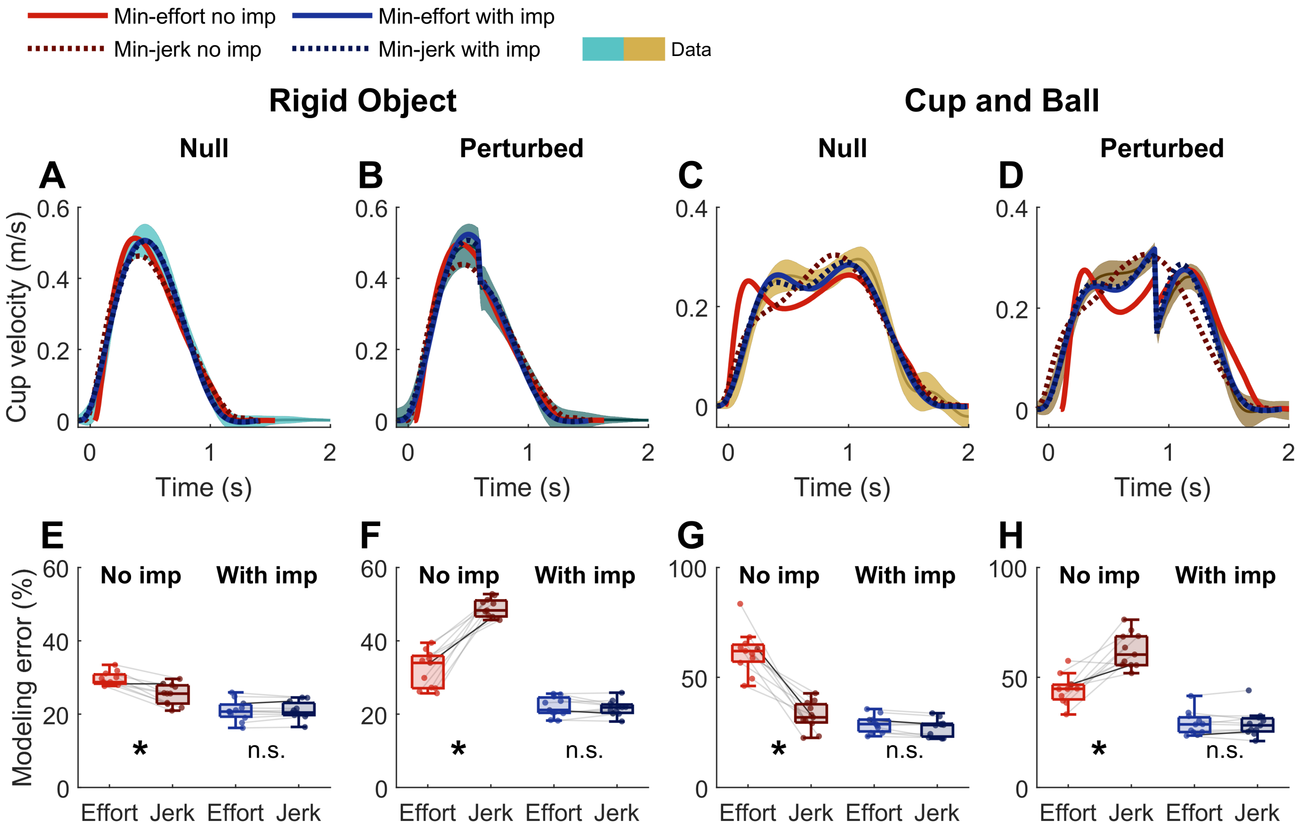

Fig S-40. Effects of the optimality criterion on the models fitted to participant #10's data

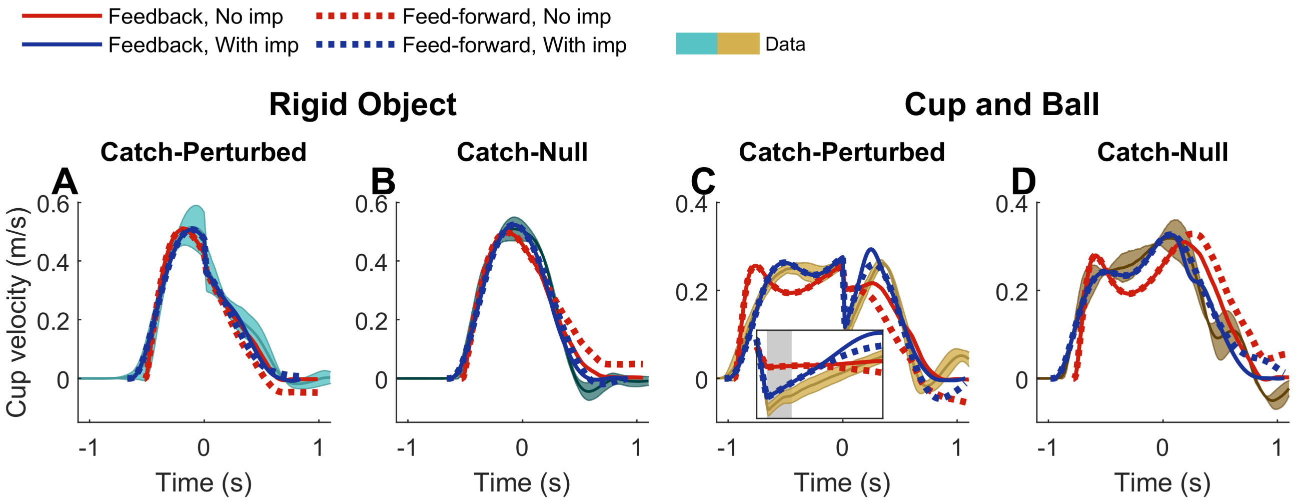

Fig S-41. Participant #10's catch trials and the the simulated behaviors.

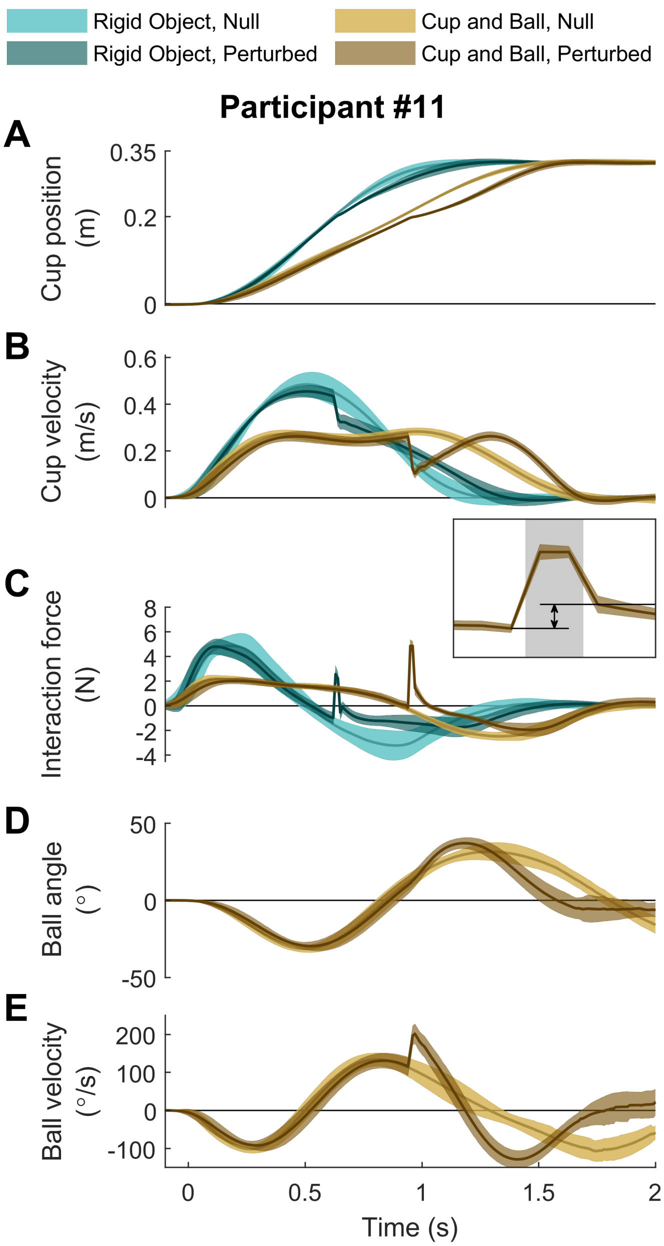

Fig S-42. Participant #11 behavior in the four blocks.

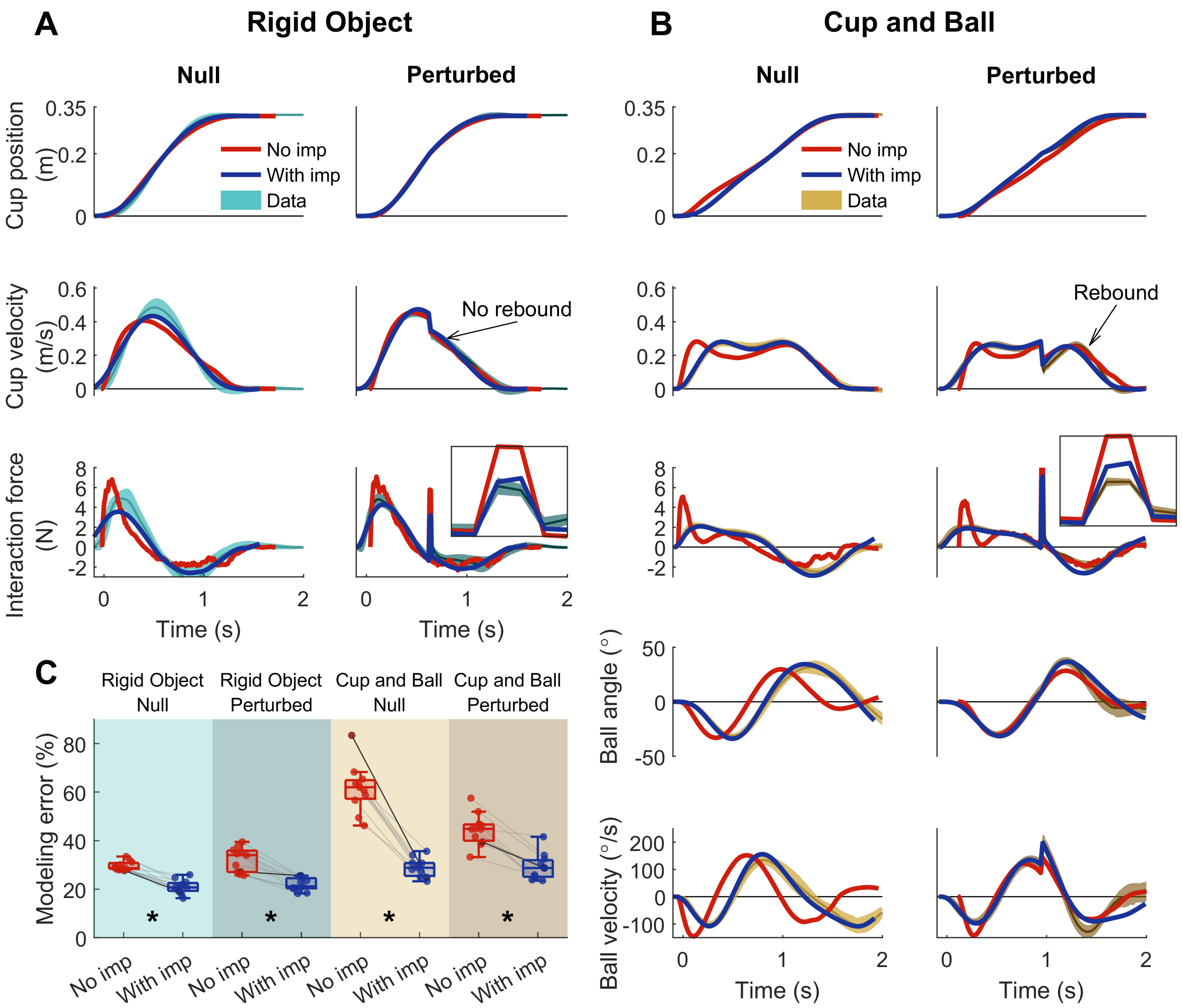

Fig S-43. Comparison of the model responses with participant #11's data.

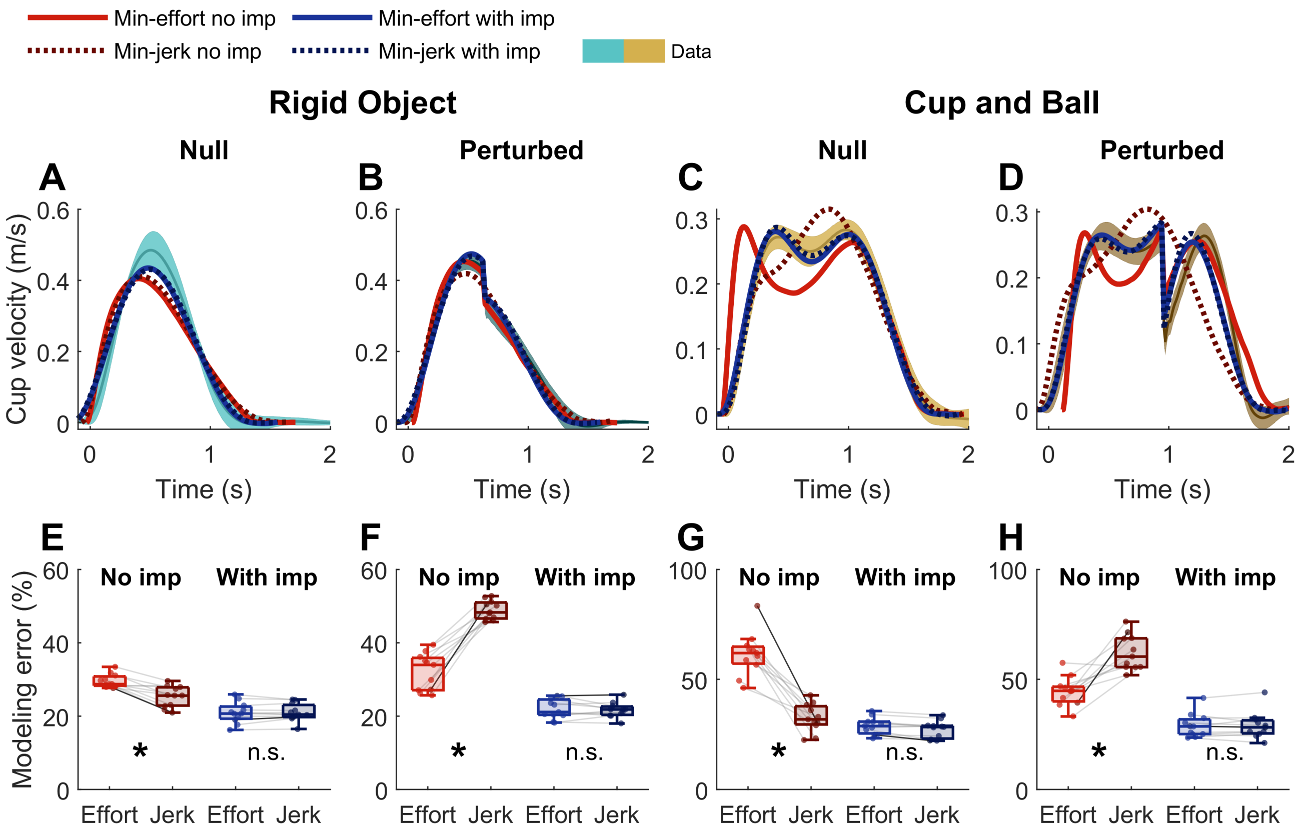

Fig S-44. Effects of the optimality criterion on the models fitted to participant #11's data

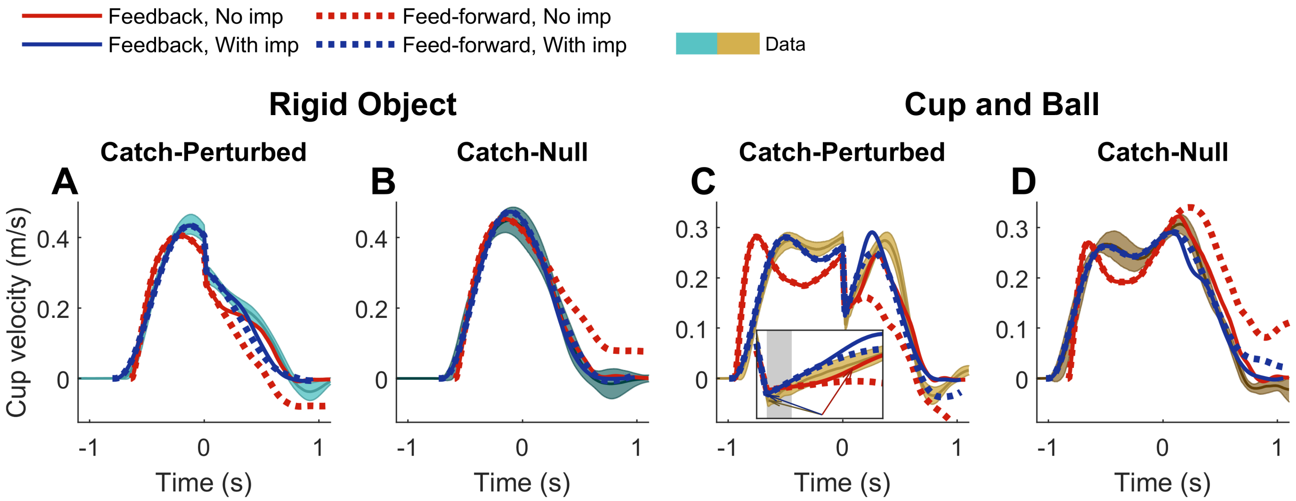

Fig S-45. Participant #11's catch trials and the the simulated behaviors.

ANOVA Results

The full ANOVA results for the three analysis on the main text is provided in **Tables S-1**, **S-2**, and **S-3**.

Table S-1. Three-way ANOVA results for the analysis of modeling error. The factors are: model type, object type, and perturbation type. Each factor has two levels.

Factors	Sum Sq	DF	F	p-value
Model type	0.617	10	309	7.56e-09
Object type	0.485	10	160	1.75e-07
Perturbation type	0.0251	10	6.63	0.0276
Model type × Object type	0.119	10	70.3	7.78e-06
Model type × Perturbation type	0.0338	10	6.37	0.0302
Object type × Perturbation type	0.0572	10	15.6	0.00273
Model type × Object type × Perturbation type	0.0479	10	29.1	0.000303

Table S-2. Four-way ANOVA results for the analysis of cost function trials. The factors are: model type, cost function, object type, and perturbation type. Each factor has two levels.

Factors	Sum Sq	DF	F	p-value
Model type	1.29	10	1070	1.69e-11
Cost function	2.11e-05	10	0.0105	0.92
Object type	0.603	10	97.4	1.8e-06
Perturbation type	0.122	10	43.6	6.03e-05
Model type × Cost function	0.000648	10	0.526	0.485
Model type × Object type	0.0902	10	37	0.000119
Cost function × Object type	0.0437	10	38	0.000106
Model type × Perturbation type	0.0859	10	36.4	0.000127
Cost function × Perturbation type	0.329	10	81.5	4.02e-06
Object type × Perturbation type	0.0119	10	3.61	0.0866
Model type × Cost function × Object type	0.0349	10	42.2	6.92e-05
Model type × Cost function × Perturbation type	0.306	10	93.7	2.14e-06
Model type × Object type × Perturbation type	0.0126	10	17.1	0.00201
Cost function × Object type × Perturbation type	0.0525	10	23.8	0.000646
Model type × Cost function × Object type × Perturbation type	0.0388	10	22.9	0.000736

Table S-3. Four-way ANOVA results for the analysis of catch trials. The factors are: model type, feedback type, object type, and perturbation type. Each factor has two levels.

Factors	Sum Sq	DF	F	p-value
Model type	4.27	10	126	5.44e-07
Feedback type	1.66	10	99.1	1.66e-06
Object type	0.00159	10	0.0949	0.764
Perturbation type	1.34	10	74.9	5.88e-06
Model type × Feedback type	0.779	10	41.6	7.32e-05
Model type × Object type	0.00843	10	0.607	0.454
Feedback type × Object type	0.19	10	41.9	7.15e-05
Model type × Perturbation type	0.0423	10	2.53	0.143
Feedback type × Perturbation type	0.00656	10	2.43	0.15
Object type × Perturbation type	0.245	10	9.52	0.0115
Model type × Feedback type × Object type	0.046	10	10.3	0.00928
Model type × Feedback type × Perturbation type	0.0358	10	46.8	4.5e-05
Model type × Object type × Perturbation type	0.00824	10	0.612	0.452
Feedback type × Object type × Perturbation type	0.0246	10	11.9	0.0062
Model type × Feedback type × Object type × Perturbation type	0.00353	10	6.97	0.0247
